# Nucleoporin foci are stress‐sensitive condensates dispensable for *C. elegans* nuclear pore assembly

**DOI:** 10.15252/embj.2022112987

**Published:** 2023-05-31

**Authors:** Laura Thomas, Basma Taleb Ismail, Peter Askjaer, Geraldine Seydoux

**Affiliations:** ^1^ HHMI and Department of Molecular Biology and Genetics Johns Hopkins University School of Medicine Baltimore MD USA; ^2^ Andalusian Center for Developmental Biology (CABD), CSIC/JA/Universidad Pablo de Olavide Seville Spain

**Keywords:** aging, *C. elegans*, condensate, nucleoporin, oocyte, Organelles, Translation & Protein Quality

## Abstract

Nucleoporins (Nups) assemble nuclear pores that form the permeability barrier between nucleoplasm and cytoplasm. Nucleoporins also localize in cytoplasmic foci proposed to function as pore pre‐assembly intermediates. Here, we characterize the composition and incidence of cytoplasmic Nup foci in an intact animal, *C. elegans*. We find that, in young non‐stressed animals, Nup foci only appear in developing sperm, oocytes and embryos, tissues that express high levels of nucleoporins. The foci are condensates of highly cohesive FG repeat‐containing nucleoporins (FG‐Nups), which are maintained near their solubility limit in the cytoplasm by posttranslational modifications and chaperone activity. Only a minor fraction of FG‐Nup molecules concentrate in Nup foci, which dissolve during M phase and are dispensable for nuclear pore assembly*.* Nucleoporin condensation is enhanced by stress and advancing age, and overexpression of a single FG‐Nup in post‐mitotic neurons is sufficient to induce ectopic condensation and organismal paralysis. We speculate that Nup foci are non‐essential and potentially toxic condensates whose assembly is actively suppressed in healthy cells.

## Graphical abstract

FG repeat‐containing nucleoporins form non‐essential, age‐ and stress‐enhanced cytoplasmic condensates in germ cells and developing embryos.

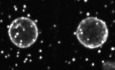

## Introduction

**Figure 1:**
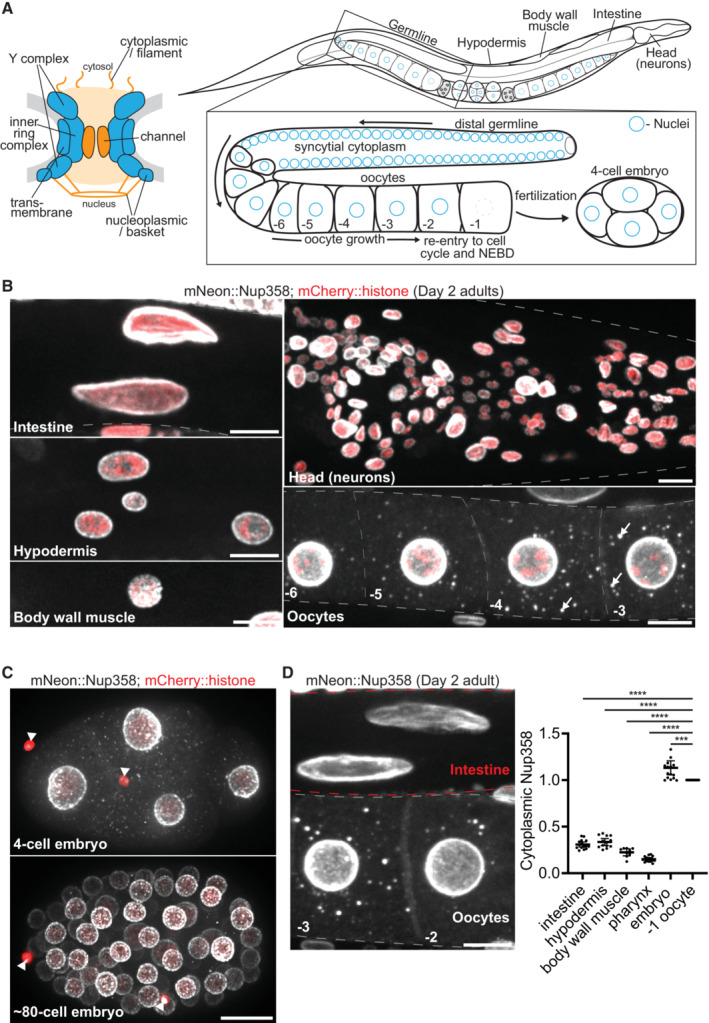
Cytoplasmic Nup foci are not present in somatic cells of young animals Left: Schematic depicting the structure of a nuclear pore complex, which consists of ~30 Nup proteins arranged in distinct subcomplexes. Blue subcomplexes are structural elements of the pore and include transmembrane Nups, the inner ring complex, and two copies of the Y complex. FG domain Nups are designated in orange and generate the permeability barrier of the central channel, and additionally localize to cytoplasmic filaments and the nuclear basket. Right: Schematic depicting the tissues and germline organization of a *C. elegans* adult hermaphrodite. Germ cell nuclei (designated in blue) proliferate in a syncytial cytoplasm before becoming enclosed by membrane to form individual oocytes. Oocytes arrest in meiosis I and grow in an assembly line‐like fashion until induced by sperm signaling to re‐enter the cell cycle in preparation for fertilization. NEBD: nuclear envelope breakdown.Representative confocal micrographs of CRISPR‐tagged mNeonGreen::Nup358 in the intestine, hypodermis, body wall muscle, head, and oocytes of Day 2 adult *C. elegans*. Nuclei are marked by a mCherry::histone transgene. White arrows denote cytoplasmic foci in oocytes.Representative confocal micrographs showing mNeonGreen::Nup358 in interphase 4‐cell versus ~80‐cell embryos. White arrowheads denote polar bodies (meiotic products).Left: Representative confocal micrograph of mNeonGreen::Nup358 in ‐2 and ‐3 oocytes and intestinal cells of a Day 2 adult. Red dashed lines denote intestinal cells, gray dashed lines outline oocytes. Right: Quantification of cytoplasmic (soluble) mNeonGreen::Nup358 signal in intestinal, hypodermal, muscle, head (pharyngeal), or early (4‐cell) embryonic cells as compared to that of the ‐1 oocyte. Values are normalized within the same animal so that the measurement for the ‐1 oocyte = 1.0. Error bars represent 95% CI for *n* > 7 animals (biological replicates). Data information: ****P < 0.0001; ***P < 0.001. Significance was determined using a one‐way ANOVA. All images in this figure are maximum intensity projections. Scale bars = 10 μm. Source data are available online for this figure.

In all eukaryotes, the double‐membraned nuclear envelope (NE) partitions the nucleoplasm from the cytoplasm and material is exchanged between the two compartments by way of nuclear pore complexes. Nuclear pore complexes are composed of at least 30 distinct nucleoporins (Nups) arranged in biochemically stable subcomplexes (Fig 1A; Cohen‐Fix & Askjaer, 2017; Hampoelz *et al*, 2019a). Approximately, two‐thirds of Nups are essential to scaffold and anchor pore complexes to the NE. The remaining one‐third contain large phenylalanine/glycine (FG) rich domains that are highly intrinsically disordered. FG‐Nups are enriched in the central channel of the pore and form multivalent interactions *in vivo* and *in vitro* (Frey *et al*, 2006; Patel *et al*, 2007; Labokha *et al*, 2012; Xu & Powers, 2013). In the ‘selective phase’ model of transport selectivity, the permeability barrier is established by cohesive interactions among FG‐Nups that form a phase separated network (Ribbeck & Görlich, 2001; Schmidt & Görlich, 2016). In support of this model, interactions among FG‐Nups are critical for the formation of the permeability barrier and FG‐Nup hydrogels recapitulate nuclear pore selectivity *in vitro* (Strawn *et al*, 2004; Frey & Görlich, 2007; Hülsmann *et al*, 2012; Schmidt & Görlich, 2015; Ng *et al*, 2021).

In addition to their localization at the NE, Nups have been observed in discrete cytoplasmic foci in yeast, oocytes, and animal cell types cultured *in vitro* (Cordes *et al*, 1996; Colombi *et al*, 2013; Raghunayakula *et al*, 2015; Ren *et al*, 2019). Cytoplasmic Nup foci have been implicated in miRNA‐mediated mRNA repression (Sahoo *et al*, 2017), nuclear pore inheritance (Colombi *et al*, 2013), and pore assembly by a condensate‐based, non‐canonical mechanism that generates annulate lamellae (Hampoelz *et al*, 2019b). Annulate lamellae are a specialized subdomain of the endoplasmic reticulum (Kessel, 1989) proposed to function as a source of ready‐made pore complexes in rapidly dividing cells (Hampoelz *et al*, 2016; Ren *et al*, 2019). Although some have argued against a stockpiling function (Stafstrom & Staehelin, 1984; Onischenko *et al*, 2004), annulate lamellae generated in oocytes have been proposed to fuel the expansion of nuclear membranes in *Drosophila* embryos (Hampoelz *et al*, 2016, 2019b).

Nups are also frequently enriched in pathological cytoplasmic inclusions that are hallmarks of neurodegenerative disease (reviewed in Fallini *et al*, 2020; Hutten & Dormann, 2020; Chandra & Lusk, 2022), leading to the proposal that Nups become sequestered and depleted from nuclear pores under disease conditions (Zhang *et al*, 2018; Gasset‐Rosa *et al*, 2019). Given the inherent propensity of FG‐Nups to form multivalent networks, it is possible that Nup condensation directly contributes to protein aggregation in disease. In support of this hypothesis, condensation of FG‐Nup fusion oncogenes drives certain cancers (Zhou & Yang, 2014; Terlecki‐Zaniewicz *et al*, 2021; Chandra *et al*, 2022), cytoplasmic Nup granules form upon loss of fragile X‐related proteins (Agote‐Aran *et al*, 2020), and cytoplasmic FG‐Nups drive aggregation of TDP‐43 in ALS/FTLD and following traumatic brain injury (Anderson *et al*, 2021; Gleixner *et al*, 2022). These observations suggest that Nups are not passive clients of cytoplasmic inclusions but rather active promoters of protein aggregation and disease progression.

Cytoplasmic Nup foci were reported previously in *C. elegans* oocytes and embryos (Pitt *et al*, 2000; Jud *et al*, 2007; Sheth *et al*, 2010; Patterson *et al*, 2011). In this study, we use the *C. elegans* model to systematically investigate the origin, regulation, and function of Nup foci. We find that, in addition to oocytes and embryos, Nup foci form in developing sperm and in the somatic tissues of aged animals. We find that the majority of Nup foci are condensates of FG‐Nups, which are maintained in a mostly soluble cytoplasmic pool by posttranslational modifications and the chaperone activity of nuclear transport receptors (NTRs). Condensation is enhanced by heat stress and FG‐Nup overexpression, which when induced in neurons can disrupt nuclear pore assembly and lead to organismal paralysis. Our findings suggest that Nup foci are incidental byproducts of the natural tendency of FG‐Nups to undergo condensation, which is required to generate the permeability barrier of nuclear pores but must be suppressed in the cytoplasm to avoid premature and potentially toxic condensation.

## Results

### In young animals Nup foci assemble only in growing oocytes, developing sperm, and early embryos

Cytoplasmic Nup foci have been observed in *C. elegans* oocytes and early embryos using the mAb414 antibody (Davis & Blobel, 1986; Pitt *et al*, 2000; Jud *et al*, 2007). To characterize the distribution of Nup foci across all *C. elegans* tissues, we used two Nups, Nup358 and Nup88, which have been reported in cytoplasmic foci in *Drosophila* oocytes, yeast, and a range of cultured cell types from different organisms (Cordes *et al*, 1996; Wu *et al*, 2001; Colombi *et al*, 2013; Raghunayakula *et al*, 2015; Sahoo *et al*, 2017; Hampoelz *et al*, 2019b). We used CRISPR genome engineering to tag Nup358 and Nup88 at their endogenous loci and examined their distribution in all tissues across hermaphrodite development (Fig 1A). As expected, both Nups localized to the NE in all cell types, including muscle, hypodermis, intestine, neurons, and germ cells (Fig 1B, Appendix Fig S1A). In the germline of hermaphrodites, germ cell nuclei proliferate in a syncytial cytoplasm before individualizing to produce sperm during the fourth larval (L4) stage and oocytes in adults (Fig 1A, Appendix Fig S1B). We detected Nups in cytoplasmic foci (“Nup foci”) in the residual body of spermatocytes, a transient structure that accumulates components discarded during spermatogenesis (Appendix Fig S1B). We also detected Nup358 and Nup88 in Nup foci in growing oocytes and in early embryos (<~80‐cell stage; Fig 1B and C, Appendix Fig S1A and C). The intensity of Nup foci in oocytes increased between days 1 and 2 of adulthood (Appendix Fig S1D). In contrast, we did not detect Nup foci in somatic cells at any stage through Day 2 of adulthood. The cytoplasmic concentration of Nups in germ cells and early embryos was ~3‐5‐fold higher than that observed in somatic cells (Fig 1D). We conclude that, in developing animals and young adults, Nup foci only form in gametes and early embryos, which accumulate higher levels of cytoplasmic Nups compared with somatic tissues.

### Nup foci in growing oocytes contain FG‐Nups and their binding partners, but not transmembrane, inner ring complex, or nucleoplasmic Nups

**Figure 2:**
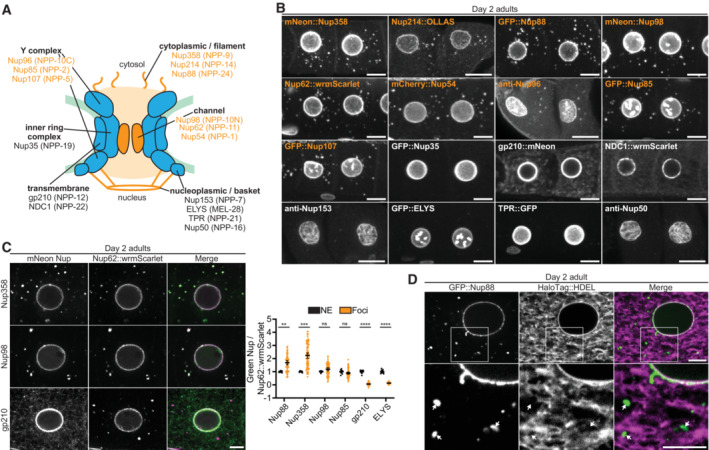
Cytoplasmic Nup foci primarily contain FG‐Nups and their binding partners Schematic depicting the nuclear pore location of the Nups examined in this study. Nups are designated using human nomenclature, followed by the *C. elegans* homolog in parentheses. Nups listed in orange localize to cytoplasmic foci in growing oocytes, whereas those denoted in black do not.Representative confocal micrographs of the ‐3 and ‐4 oocytes from Day 2 adult *C. elegans* expressing tagged versions of each indicated Nup or stained with anti‐Nup antibodies. All images are maximum intensity projections, with the exception of gp210 and NDC1 which are single imaging planes. Orange labels designate Nups enriched in cytoplasmic foci.Left: Representative confocal micrographs of Day 2 adult oocytes comparing the localization of CRISPR‐tagged Nup62::wrmScarlet to mNeonGreen‐tagged Nup358, Nup98 and gp210. Right: Quantification of the overlap between Nup62::wrmScarlet and each indicated Nup at the nuclear envelope (NE) versus cytoplasmic foci. Each point designates an individual nucleus or focus. Values are normalized so that the average ratio at the nuclear envelope = 1.0. Error bars represent 95% CI for *n* > 7 (nuclei) or *n* > 59 (foci).Representative confocal micrographs showing partial overlap of CRISPR‐tagged GFP::Nup88 with the luminal endoplasmic reticulum/nuclear envelope marker HaloTag::HDEL in a Day 2 adult oocyte. 20% of foci completely overlapped with HaloTag::HDEL, 64% partially overlapped, and 16% showed no overlap with HaloTag::HDEL (*n* = 118, see Materials and Methods). Areas indicated by white boxes are magnified below; white arrows indicate foci that do not completely overlap with the endoplasmic reticulum. Data information: ****P < 0.0001; ***P < 0.001; **P < 0.01; ns, not significant. Significance was determined using an unpaired *t*‐test. Scale bars = 10 μm (panel B) or 5 μm (panels C and D). Source data are available online for this figure.

Nup foci have been proposed to correspond to (i) condensates containing pore assembly intermediates, or (ii) mature pore complexes in membranous annulate lamellae (Raghunayakula *et al*, 2015; Hampoelz *et al*, 2016, 2019b; Ren *et al*, 2019). To systematically compare the composition and stoichiometry of Nup foci to that of mature nuclear pore complexes at the NE, we used a collection of genomically‐encoded tags, transgenes, and antibodies against 16 Nups (including representatives of each nuclear pore subcomplex) as well as the Nup358 binding partners RanGAP and NXF1 (Fig 2A, Appendix Table S1). We examined Nup distribution in growing oocytes of Day 2 adult wild‐type hermaphrodites where Nup foci are prominent.

As expected, all Nups tested localized to the NE (Fig 2B, Appendix Fig S2A). Nuclear basket and Y complex Nups additionally localized to the nucleoplasm and meiotic chromosomes, respectively, as previously described (Gómez‐Saldivar *et al*, 2016; Hattersley *et al*, 2016). Only a subset of Nups localized to cytoplasmic foci, including FG‐Nups of the central channel and cytoplasmic filaments (Nup62, Nup98, Nup214, and Nup358) and their binding partners (Y complex Nups, Nup88, RanGAP, and NXF1; Fig 2B, Appendix Fig S2A and B). The transmembrane Nups gp210 and NCD1 could be detected throughout the endoplasmic reticulum as described previously (Galy *et al*, 2008; Huelgas‐Morales *et al*, 2020; Mauro *et al*, 2022), but did not enrich in foci, nor did Nup35, an inner ring complex Nup. All nucleoplasmic‐facing Nups (Nup153, Nup50, TPR, and ELYS) were enriched in the nucleoplasm and absent from cytoplasmic foci. The assembly‐line arrangement of the *C. elegans* germline allowed us to visualize Nup distribution throughout oocyte growth and maturation. We found that nucleoplasmic Nups and the inner ring complex component Nup35 never became incorporated into Nup foci during oocyte growth (Appendix Fig S2C). We also analyzed the distribution of Nups in 4‐cell stage early embryos and obtained the same results except for Nup35, which did not localize to foci in oocytes but did in embryos (Appendix Fig S2D and E). We conclude that Nup foci in growing oocytes primarily enrich cytoplasm‐facing FG‐Nups and their binding partners (Fig 2A).

Co‐staining experiments using the mAb414 antibody, which in vertebrates recognizes Nup62, Nup153, Nup214, and Nup358, suggested that Nup foci contain multiple Nups (Appendix Fig S2A and E). To examine Nup stoichiometry in the foci, we crossed a subset of GFP‐tagged Nups pairwise with Nup62::wrmScarlet. As expected, all Nups tested colocalized with Nup62::wrmScarlet at the NE (Fig 2C). Nups that localize to cytoplasmic foci (Nup85, Nup88, Nup98, and Nup358) additionally colocalized with Nup62::wrmScarlet in all foci. Quantification of the ratio of the GFP‐tagged Nup to Nup62::wrmScarlet revealed that each Nup accumulates in fixed stoichiometry relative to Nup62 at the NE. In contrast, Nups exhibited variable stoichiometry in the cytoplasmic foci (Fig 2C). We also found that only 20% of GFP::Nup88 foci in growing oocytes fully overlapped with a marker for endoplasmic reticulum membranes (Fig 2D). This is consistent with our observation that gp210 and NDC1, which both localize to the endoplasmic reticulum (Galy *et al*, 2008; Mauro *et al*, 2022), are not enriched in Nup foci. We conclude that the majority of Nup foci in growing oocytes are unlikely to correspond to stockpiled mature pores, as they lack critical pore scaffolds, exhibit variable Nup stoichiometry, and rarely associate with endoplasmic reticulum membranes.

### Nup foci are condensates scaffolded by excess FG‐Nups

*In vitro*, FG‐Nups condense into hydrogels (Frey *et al*, 2006; Labokha *et al*, 2012; Schmidt & Görlich, 2015) raising the possibility that cytoplasmic Nup foci might form by spontaneous condensation of FG‐Nups in the saturated environment of the oocyte. Condensation is highly sensitive to concentration: proteins de‐mix into dense and dilute phases when their concentration exceeds the saturation concentration (c_sat_), the maximum concentration allowed in the soluble, dilute phase (Alberti *et al*, 2019).

**Figure 3:**
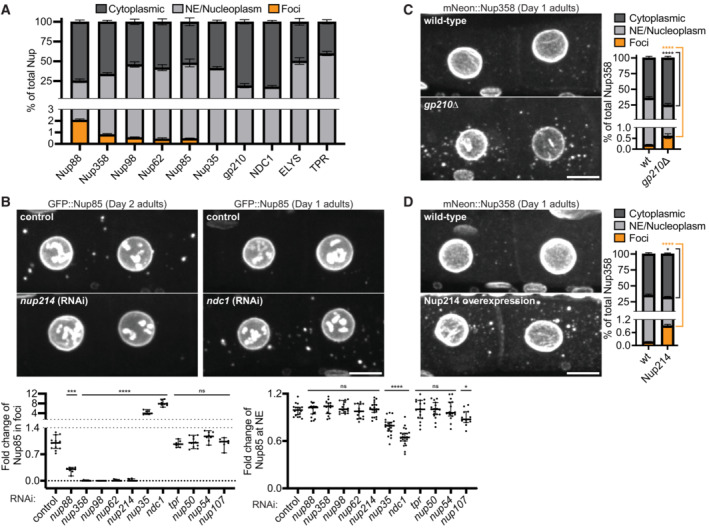
Nup foci are condensates scaffolded by cytoplasmic facing FG‐Nups Quantification of the distribution of CRISPR‐tagged Nups between the cytoplasm (soluble), nuclear envelope (NE)/nucleoplasm, and cytoplasmic foci (see Materials and Methods). Measurements were made using the ‐3 and ‐4 oocytes of Day 2 adults. Error bars represent 95% CI for *n* > 5 germlines (biological replicates).Top: Representative confocal micrographs showing ‐3 and ‐4 oocytes of Day 1 or Day 2 adults with CRISPR‐tagged GFP::Nup85. *nup214* RNAi is representative of a treatment that largely abolishes Nup foci, whereas *ndc1* RNAi enhanced Nup foci. Left graph: Quantification of the total percent of GFP::Nup85 in foci following each RNAi treatment. Values are normalized so that the average control measurement = 1.0. Error bars represent 95% CI for *n* > 7 germlines (biological replicates). Right graph: Line‐scan quantification measuring GFP::Nup85 signal at the NE following each RNAi treatment. Values are normalized so that the average control measurement = 1.0. Error bars represent 95% CI for *n* > 13 nuclei (biological replicates).Left: Representative confocal micrographs showing CRISPR‐tagged mNeonGreen::Nup358 in ‐3 and ‐4 oocytes of Day 1 wild‐type versus *gp210∆* adults. Right: Quantification of the distribution of mNeonGreen::Nup358 between the cytoplasm, NE/nucleoplasm, and cytoplasmic foci in wild‐type versus *gp210∆* oocytes. Error bars represent 95% CI for *n* > 6 germlines (biological replicates).Left: Representative confocal micrographs showing mNeonGreen::Nup358 in ‐3 and ‐4 oocytes of Day 1 adults with or without overexpression of Nup214::wrmScarlet. Right: Quantification of the distribution of mNeonGreen::Nup358 between the cytoplasm, NE/nucleoplasm, and cytoplasmic foci in wild‐type oocytes versus those with Nup214 overexpression. Error bars represent 95% CI for *n* > 9 germlines (biological replicates). Data information: ****P < 0.0001; ***P < 0.001; *P < 0.05; ns, not significant. For panel B significance was determined using a one‐way ANOVA; for all other panels significance was determined using an unpaired *t*‐test. All images in this figure are maximum intensity projections. Scale bars = 10 μm. Source data are available online for this figure.

To estimate the percent of Nup molecules that undergo condensation, we used Imaris software to quantify Nup fluorescence in nuclei, the cytoplasm, and cytoplasmic foci (Appendix Fig S3A and see Materials and Methods). Remarkably, we found that the vast majority of Nups distribute between a nuclear pool (~30–40%) and a diffuse cytoplasmic pool (~60–70%), with less than 3% of Nup molecules in foci (Fig 3A). The soluble cytoplasmic pool is the least concentrated but largest by volume and is readily visualized in sum projection micrographs (Appendix Fig S3B). These observations suggest that FG‐Nups are maintained in oocytes at concentrations just in excess of saturation, such that most molecules are soluble and only a minority condense in the foci. If so, we predicted that removal of individual FG‐Nups may be sufficient to drop below the threshold for condensation and reduce foci formation.

We used RNAi and mutagenesis to systematically deplete individual Nups and examined the effect on Nup foci formation. As expected, depletion of non‐FG or nucleoplasmic Nups, which are not present in foci, had no effect on foci formation (Fig 3B, Appendix Fig S3C–E). In contrast, depletion of individual cytoplasm‐facing FG‐Nups (Nup62, Nup98, Nup214, or Nup358) reduced the formation of Nup foci by >95% without affecting Nup levels at the NE. Depletion of Nup88, which is structured but interacts with multiple subcomplexes containing FG‐Nups (Fornerod *et al*, 1997; Griffis *et al*, 2003; Xylourgidis *et al*, 2006; Yoshida *et al*, 2011), reduced Nup foci by ~70%, suggesting that interactions among FG‐Nup subcomplexes contribute to foci formation. As expected for structural Nups (Mansfeld *et al*, 2006; Stavru *et al*, 2006; Onischenko *et al*, 2009; Ródenas *et al*, 2009; Mauro *et al*, 2022), loss of Nup35 or the transmembrane Nups NDC1 or gp210 decreased Nup levels at the NE (Fig 3B and C, Appendix Fig S3C and F), and enhanced foci formation, presumably because impaired pore assembly liberates FG‐Nups to the cytoplasm. We conclude that Nup foci assembly in oocytes depends primarily on the cumulative effect of high concentrations of the FG‐Nups Nup62, Nup98, Nup214, and Nup358 in the cytoplasm.

To directly test whether high levels of FG‐Nups are sufficient to drive foci formation, we generated a transgenic strain with an extra copy of *nup214::wrmScarlet* expressed under the control of the germline‐specific *mex‐5* promoter (Fan *et al*, 2020). We found that overexpression of Nup214::wrmScarlet was sufficient to increase the proportion of endogenous mNeonGreen::Nup358 in Nup foci by 4‐fold (Fig 3D, Appendix Fig S3G).

*In vitro*, some Nup98 FG‐domain hydrogels have been shown to be dissolved by the aliphatic alcohol 1,6‐hexanediol (Schmidt & Görlich, 2015), which disrupts hydrophobic interactions and has been reported to dissolve Nup foci in yeast, *Drosophila*, and HeLa cells (Patel *et al*, 2007; Hampoelz *et al*, 2019b; Agote‐Aran *et al*, 2020). As expected, we found that hexanediol treatment reduced the intensity of Nup foci in embryos (Appendix Fig S3H). We conclude that Nup foci are FG‐Nup condensates that arise when the cytoplasmic concentration of FG‐Nups exceeds the saturation concentration.

### Nup foci assembly is enhanced by oocyte arrest, heat stress, and aging

**Figure 4:**
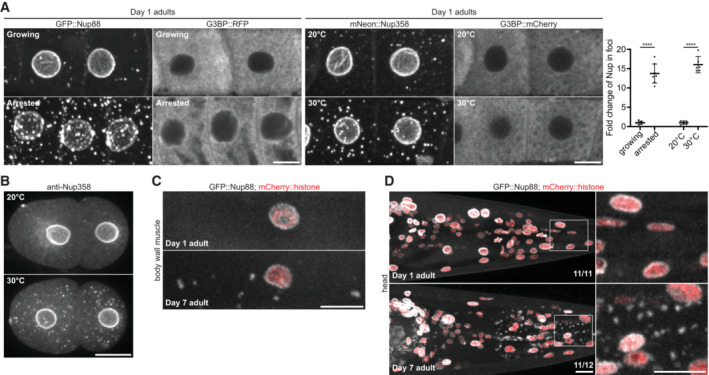
Cytoplasmic Nup foci increase with oocyte arrest, heat stress, and age Left: Representative confocal micrographs of growing ‐3 and ‐4 oocytes or ‐3, ‐4, and ‐5 arrested oocytes in Day 1 adults expressing GFP::Nup88 or the stress granule marker G3BP::RFP. GFP::Nup88 expressing oocytes are in wild‐type hermaphrodites (growing oocytes) or *fog‐2*(*q71*) unmated females (arrested oocytes). G3BP::RFP expressing oocytes are in mated (growing oocytes) or unmated (arrested oocytes) *fog‐2*(*q71*) females. Middle: Representative confocal micrographs showing CRISPR‐tagged mNeonGreen::Nup358 and G3BP::mCherry in the ‐3 and ‐4 growing oocytes of Day 1 adults maintained at 20°C or after a 20 min shift to 30°C. Right: Quantification of the percent of GFP::Nup88 in foci in growing versus arrested oocytes or mNeonGreen::Nup358 in foci at 20°C versus 30°C. Error bars represent 95% CI for *n* > 7 germlines (growing vs. arrested; biological replicates) or *n* = 6 germlines (20°C versus 30°C; biological replicates). Values are normalized so that the average control condition (growing oocytes or 20°C) measurement = 1.0. See Appendix Fig S4A for raw (non‐normalized) values of the distribution of Nup between the cytoplasm (soluble), nuclear envelope (NE)/nucleoplasm, and cytoplasmic foci for each condition.Representative confocal micrographs of endogenous Nup358 in 2‐cell interphase embryos grown at 20°C or after shifting to 30°C for 20 min.Representative confocal micrographs showing GFP::Nup88 in body wall muscle cells of Day 1 versus Day 7 adults. Nuclei are marked by a mCherry::histone transgene.Representative confocal micrographs showing GFP::Nup88 in the head of a Day 1 versus Day 7 adult. Nuclei are marked by a mCherry::histone transgene. Areas indicated by white boxes are magnified at right. 100% (*n* = 11) of Day 1 adults lacked foci in somatic cells whereas cytoplasmic foci were observed outside of the germline in 92% (*n* = 12) of Day 7 adults. Data information: ****P < 0.0001. Significance was determined using an unpaired *t*‐test. All images in this figure are maximum intensity projections, with the exception of G3BP (panel A) which are single focal planes. Scale bars = 10 μm. Source data are available online for this figure.

Oocyte production occurs continuously in young hermaphrodites such that fully grown oocytes are immediately ovulated and fertilized. In contrast, in animals lacking sperm, fully grown oocytes arrest and are stored in the oviduct. Electron microscopy studies have reported annulate lamellae in ~10% of arrested oocytes in *C. elegans* (and 42% of arrested oocytes in the related nematode *C. remanei*), but not in the growing oocytes of hermaphrodites or in embryos (Pitt *et al*, 2000; Patterson *et al*, 2011; Langerak *et al*, 2019). To examine Nup foci in arrested oocytes, we used unmated *fog‐2*(*q71*) females which do not produce sperm and accumulate fully grown arrested oocytes in the oviduct (Schedl & Kimble, 1988). We observed a 14‐fold increase in the percent of GFP::Nup88 in foci in the arrested oocytes of Day 1 adult *fog‐2*(*q71*) females compared to growing oocytes of age‐matched wild‐type hermaphrodites (Fig 4A, Appendix Fig S4A). The Nup foci in arrested oocytes enriched additional Nups at low levels including Nup35 and ELYS (Appendix Fig S4B). Furthermore, 42% of Nup foci in arrested oocytes overlapped with a marker for endoplasmic reticulum membranes (Appendix Fig S4C), raising the possibility that a subset of Nup foci in arrested oocytes could correspond to annulate lamellae. We also observed the formation of Nup‐rich ‘blebs’ at the NE of arrested oocytes (Fig 4A, Appendix Fig S4A). These findings are consistent with prior studies which found that the abundance of cytoplasmic Nup foci and nuclear blebs were significantly increased in arrested oocytes of *C. elegans* and related nematodes (Jud *et al*, 2007; Patterson *et al*, 2011).

Previous studies have reported parallels between oocyte arrest and environmental stresses in inducing the formation of condensates in *C. elegans* oocytes (Jud *et al*, 2008; Patterson *et al*, 2011; Elaswad *et al*, 2022). In agreement with these findings, we found that a 20 min shift from 20°C to 30°C was sufficient to increase the intensity of Nup foci in growing oocytes by 16‐fold (Fig 4A, Appendix Fig S4A and D) and significantly increase Nup condensation in embryos (Fig 4B). In contrast, the same conditions of oocyte arrest and mild heat stress did not change the distribution of the stress granule protein G3BP, which requires exposure to higher temperatures to condense (Abbatemarco *et al*, 2021; Fig 4A). Together these observations indicate that Nup foci assembly is readily enhanced by mild stress conditions. Consistent with this view, we also found that Nup foci accumulate in somatic cells in > 90% of Day 7 adult hermaphrodites, compared to 0% at Day 1 of adulthood (Fig 4C and D). We conclude that Nup foci are stress‐sensitive structures that accumulate with age.

### Nup foci disassemble during the oocyte‐to‐embryo transition and are not required for nuclear pore assembly in embryos

**Figure 5:**
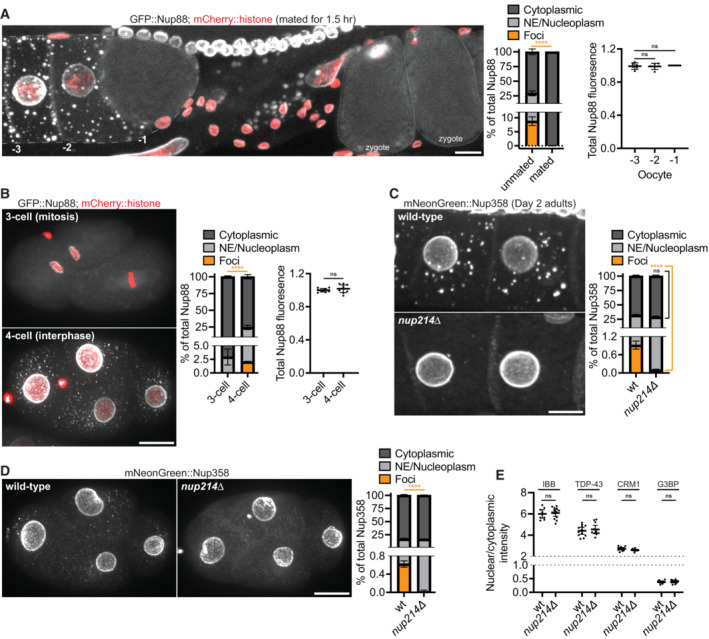
Nup foci are transient condensates that are not required for nuclear pore biogenesis Left: Representative confocal micrograph showing CRISPR‐tagged GFP::Nup88 in oocytes and newly fertilized zygotes of a *fog‐2*(*q71*) female 1.5 h post‐mating. The fluorescent foci outside of the germline and zygotes are autofluorescent intestinal gut granules. Middle: Quantification of the distribution of GFP::Nup88 between the cytoplasm (soluble), nuclear envelope (NE)/nucleoplasm, and cytoplasmic foci in ‐1 oocytes of *fog‐2*(*q71*) females unmated or mated for 1.5 h. Error bars represent 95% CI for *n* = 6 oocytes (biological replicates). Right: Total GFP::Nup88 fluorescence in ‐1, ‐2, and ‐3 position oocytes following mating for 1.5 h. Values are normalized within the same germline so that the ‐1 oocyte measurement = 1.0. Error bars represent 95% CI for *n* = 6 germlines (biological replicates).Left: Representative confocal micrographs showing GFP::Nup88 in 3‐cell (mitosis) versus 4‐cell (interphase) embryos. Middle: Quantification of the distribution of GFP::Nup88 between the cytoplasm, NE/nucleoplasm, and cytoplasmic foci. Error bars represent 95% CI for *n* > 6 embryos (biological replicates). Right: Quantification of total GFP::Nup88 fluorescence in 3‐cell (mitosis) versus 4‐cell (interphase) embryos. Values are normalized so that the average fluorescence of 3‐cell embryos = 1.0. Error bars represent 95% CI for *n* > 6 embryos (biological replicates).Left: Representative confocal micrographs showing CRISPR‐tagged mNeonGreen::Nup358 in ‐3 and ‐4 oocytes of wild‐type versus *nup214∆* Day 2 adults. Right: Quantification of the distribution of mNeonGreen::Nup358 between the cytoplasm (soluble), NE/nucleoplasm, and cytoplasmic foci in wild‐type versus *nup214∆* oocytes. Error bars represent 95% CI for *n* > 8 germlines (biological replicates).Left: Representative confocal micrographs showing mNeonGreen::Nup358 in wild‐type versus *nup214∆* interphase 4‐cell embryos. Right: Quantification of the distribution of mNeonGreen::Nup358 between the cytoplasm (soluble), NE/nucleoplasm, and cytoplasmic foci in wild‐type versus *nup214∆* embryos. Error bars represent 95% CI for *n* = 5 embryos (biological replicates).Quantification of the nuclear/cytoplasmic ratio of an IBB_domain_::mNeonGreen reporter or CRISPR‐tagged TDP‐43::wrmScarlet, CRM1::mNeonGreen, and G3BP::mCherry in 28‐cell stage embryos. Values are normalized so that the average wild‐type measurement = 1.0. Error bars represent 95% CI for *n* > 9 embryos (IBB_domain_::mNeonGreen), *n* > 11 embryos (TDP‐43::wrmScarlet), *n* > 11 embryos (CRM1::mNeonGreen), or *n* = 11 embryos (G3BP::mCherry; biological replicates). Data information: ****P < 0.0001; ns, not significant. For panel A (right graph) significance was determined using a one‐way ANOVA; for all other panels significance was determined using an unpaired *t*‐test. All images in this figure are maximum intensity projections. Scale bars = 10 μm. Source data are available online for this figure.

In the presence of sperm, oocytes at the ‐1 position in the oviduct initiate meiotic M phase in preparation for ovulation and fertilization (Huelgas‐Morales & Greenstein, 2018). We found that oocytes in M phase, whether in hermaphrodites or mated females, lack Nup foci (Fig 5A, Appendix Fig S5A). In both hermaphrodites and mated females, Nup levels did not decrease in M phase oocytes lacking foci (Fig 5A, Appendix Fig S5A), indicating that the absence of visible foci is not due to Nup degradation. Similarly, Nup foci were absent in zygotes undergoing meiosis (Fig 5A) and blastomeres undergoing mitosis, and reappeared during interphase with no change in overall Nup levels (Fig 5B, Movies EV1 and EV2). Moreover, the concentration of soluble cytoplasmic Nup increased in M phase oocytes of hermaphrodites as well as mitotic embryos (Appendix Fig S5B). These observations suggest that FG‐Nup solubility oscillates with the cell cycle, peaking during M phase, consistent with prior studies (Pitt *et al*, 2000; Onischenko *et al*, 2005). We conclude that Nup foci are transient structures that are not maintained during the oocyte‐to‐embryo transition.

To test whether Nup foci contribute to pore assembly in embryos, we used CRISPR genome engineering to generate a complete deletion of the *nup214* locus. Consistent with our RNAi results, using four independent markers (mNeonGreen::Nup358, GFP::Nup85, RanGAP::wrmScarlet, and mAb414), we found that Nup foci were greatly reduced in growing oocytes and early embryos of *nup214∆* hermaphrodites (Fig 5C and D, Appendix Fig S5C–E). We also found that GFP::Nup88 was largely localized to the cytoplasm in the *nup214∆* mutant (Appendix Fig S5F), supporting a role for Nup214 in stabilizing and targeting Nup88 to pore complexes (Xylourgidis *et al*, 2006). Despite lacking robust Nup foci, *nup214∆* embryos were viable (Appendix Fig S5G). Furthermore, nuclear pore formation was not disrupted in *nup214∆* mutant embryos (Appendix Fig S5H), as evidenced by normal partitioning of cargos between the nucleus and cytoplasm, including the nuclear RNA‐binding protein TDP‐43 and an importin β binding (IBB) domain reporter (Lott & Cingolani, 2011; Fig 5E). We obtained similar results in a *nup88∆* mutant, which also reduces the incidence of Nup foci: *nup88∆* mutants were viable and had normal partitioning of cargo between the nucleus and cytoplasm (Appendix Fig S5C and I–K). We conclude that robust Nup foci are not essential for viability or nuclear pore assembly during embryogenesis.

As the number and size of Nup foci increase significantly during oocyte arrest (Fig 4), we considered whether Nup foci might serve an essential function specifically in arrested oocytes. *nup214∆* mutant females exhibited a ~40% reduction in Nup foci in arrested oocytes compared with wild‐type (Appendix Fig S5L). Remarkably, we also observed an ~26% increase in Nup levels at the NE, presumably because reduced condensation in foci liberates Nups to associate with the NE (Appendix Fig S5L). Using timed matings of *nup214∆* and wild‐type females (see Materials and Methods), we found that embryos produced from 1‐day old arrested *nup214∆* or wild‐type oocytes were equally viable (Appendix Fig S5M). We conclude that robust assembly of Nup foci in arrested oocytes is not essential to support embryonic development.

### Multiple mechanisms enhance Nup solubility in the cytoplasm

**Figure 6:**
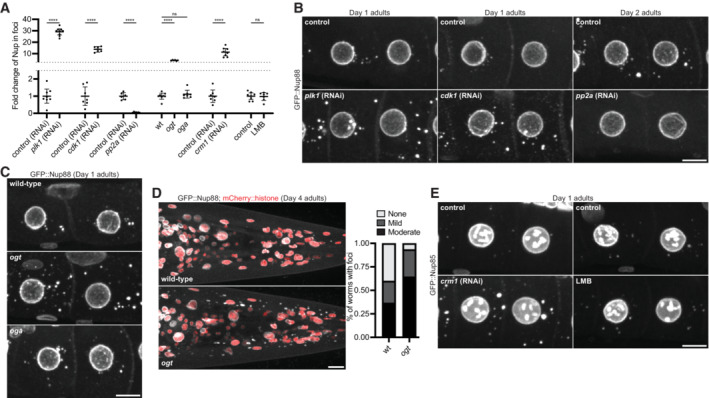
Phosphorylation, GlcNAcylation, and CRM1 promote Nup solubility Quantification of the relative Nup intensity in foci in each indicated condition compared to control. Values are normalized so that the average control condition measurement = 1.0. Error bars represent 95% CI (biological replicates) and data correspond to micrographs in Fig 6B (*plk1* RNAi, *n* > 8 germlines; *cdk1* RNAi, *n* > 6 germlines; *pp2A* RNAi, *n* > 6 germlines), Fig 6C (*ogt* and *oga* mutants, *n* > 6 germlines), and Fig 6E (*crm1* RNAi, *n* > 7 germlines; LMB treatment, *n* > 8 germlines). See Appendix Fig S6B for raw (non‐normalized) values for the distribution of each Nup between the cytoplasm (soluble), nuclear envelope (NE)/nucleoplasm, and cytoplasmic foci for each condition.Representative confocal micrographs showing CRISPR‐tagged GFP::Nup88 in ‐3 and ‐4 oocytes depleted of PLK1, CDK1, or the PP2A scaffolding subunit PAA‐1. Day 1 adults were used for kinase depletion, and Day 2 adults were used for phosphatase depletion.Representative confocal micrographs showing GFP::Nup88 in ‐3 and ‐4 oocytes of wild‐type, *ogt*, or *oga* mutant Day 1 adults.Left: Representative confocal micrographs showing GFP::Nup88 in the head of wild‐type versus *ogt* mutant Day 4 adults. Right: Quantification of the number of wild‐type versus *ogt* mutant Day 4 adults lacking foci (none), or with mild or moderate cytoplasmic foci in somatic tissues. *n* > 30 animals for each genotype.Left: Representative confocal micrographs showing CRISPR‐tagged GFP::Nup85 in ‐3 and ‐4 control oocytes or oocytes depleted of CRM1. Right: Representative confocal micrographs showing GFP::Nup85 in ‐3 and ‐4 oocytes of control animals or following treatment with the CRM1 inhibitor leptomycin B (LMB). All images are from Day 1 adults. Data information: ****P < 0.0001; ns, not significant. For the *ogt* and *oga* mutants significance was determined using a one‐way ANOVA; for all other conditions significance was determined using an unpaired *t*‐test. All the images in this figure are maximum intensity projections. Scale bars = 10 μm. Source data are available online for this figure.

The observation that Nup foci disassemble during M phase suggests that cell cycle regulators modulate Nup solubility. PLK1 and CDK1 are two kinases that are active in oocytes and known to drive nuclear pore disassembly during NE breakdown in M phase (Chase *et al*, 2000; De Souza *et al*, 2004; Onischenko *et al*, 2005; Laurell *et al*, 2011; Rahman *et al*, 2015; Linder *et al*, 2017; Martino *et al*, 2017; Huelgas‐Morales & Greenstein, 2018; Kutay *et al*, 2021). CDK1 enriches in Nup foci in *C. elegans* oocytes (Appendix Fig S6A) consistent with prior observations in *Xenopus* oocytes (Beckhelling *et al*, 2003). We found that RNAi depletion of PLK1 and CDK1 increased the proportion of GFP::Nup88 in foci and at the NE in growing oocytes (Fig 6A and B, Appendix Fig S6B and C). Inhibition of the phosphatase PP2A blocks nuclear pore complex and Nup foci assembly in *Drosophila* embryos (Onischenko *et al*, 2005). Consistently, RNAi depletion of the scaffolding subunit of PP2A led to a striking loss of GFP::Nup88 from foci and the NE (Fig 6A and B, Appendix Fig S6B and C). These observations suggest that, in addition to regulating nuclear pore assembly, Nup phosphorylation by cell cycle kinases increases the solubility of Nups in the cytoplasm and the phosphatase PP2A counteracts this effect.

FG domains are heavily modified by O‐GlcNAcylation, a modification proposed to limit FG domain interactions within the nuclear pore central channel (Ruba & Yang, 2016; Yoo & Mitchison, 2021). O‐GlcNAcylation is catalyzed by the enzyme O‐GlcNAc transferase (OGT), which enriches in Nup foci in oocytes (Appendix Fig S6D). *ogt* mutant animals lack Nup O‐GlcNAcylation as previously described (Appendix Fig S6E and F; Hanover *et al*, 2005) and, remarkably, exhibit enhanced Nup foci (Fig 6A and C, Appendix Fig S6B and G). We also visualized Nup foci in a loss of function allele of the *C. elegans* O‐GlcNAcase (OGA) reported to exhibit higher levels of Nup O‐GlcNAcylation in embryos (Forsythe *et al*, 2006). We did not detect a significant change in Nup foci in the *oga* mutant, suggesting that, in oocytes, Nups may be sufficiently O‐GlcNAcylated such that loss of OGA activity does not affect Nup solubility.

To test whether O‐GlcNAcylation contributes to Nup solubility outside of the germline, we examined *ogt* mutant animals for Nup foci in somatic tissues. We found that, by Day 4 of adulthood, *ogt* mutant animals had a higher incidence of Nup foci in somatic cells compared to wild‐type (Fig 6D), suggesting a role for O‐GlcNAcylation in promoting FG‐Nup solubility in both soma and germline tissues.

Recent studies have suggested that NTRs function as “chaperones” to prevent aggregation of intrinsically disordered proteins, including Nups (Milles *et al*, 2013; Guo *et al*, 2018; Hofweber *et al*, 2018; Hutten *et al*, 2020; Khalil *et al*, 2022). We found that two endogenously tagged NTRs (CRM1 and transportin) are enriched in cytoplasmic Nup foci in *C. elegans* oocytes (Appendix Fig S7A and B). The exportin CRM1 makes high affinity interactions with the FG‐Nups Nup214 and Nup358 (Port *et al*, 2015; Ritterhoff *et al*, 2016; Tan *et al*, 2018). As we found Nup214 and Nup358 to be key scaffolds for Nup foci (see Fig 3B), we next tested whether CRM1‐binding promotes the solubility of cytoplasmic Nups. Consistent with a solubilizing effect for CRM1 interaction, RNAi depletion of CRM1 led to an increase in Nup foci formation (Fig 6A and E, Appendix Figs S6B and S7C and D). This effect is unlikely to be due to impaired nuclear export, as Nup foci were not altered in worms treated for 4 h with the CRM1 inhibitor leptomycin B (LMB; Fig 6A and E, Appendix Figs S6B and S7D and E). Despite enrichment of transportin at Nup foci, RNAi depletion of transportin did not affect Nup solubility (Appendix Fig S7F and G). In summary, these results suggest that Nup solubility is enhanced by multiple mechanisms including phosphorylation, O‐GlcNAcylation, and CRM1 binding.

### Ectopic Nup condensation in neurons is toxic

**Figure 7:**
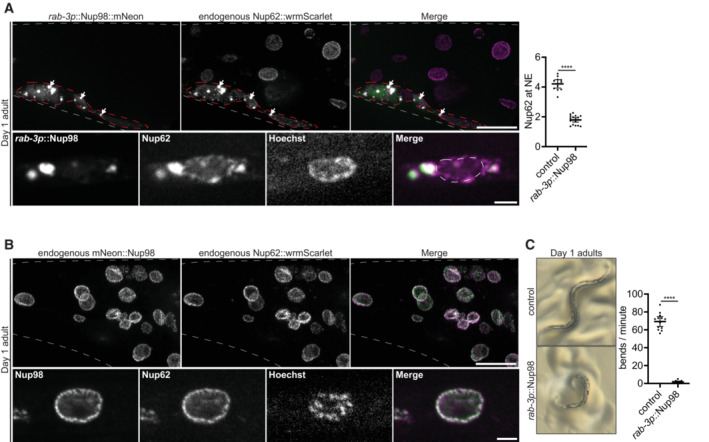
Ectopic Nup98 foci in neurons deplete an endogenous Nup from the nuclear envelope and cause paralysis Top left: Representative confocal micrographs showing localization of endogenous CRISPR‐tagged Nup62::wrmScarlet relative to transgenic *rab‐3p*::Nup98::mNeonGreen in the tail of a Day 1 adult. Gray dashed lines indicate the boundary of the tail. Transgenic *rab‐3p*::Nup98::mNeonGreen is only expressed in neurons, which are designated by the red dashed outline. White arrows indicate enrichment of endogenous Nup62 in ectopic Nup98 foci. Bottom left: Representative confocal micrographs showing endogenous Nup62 depletion from the nuclear envelope. White dashed lines indicate the boundary of the nucleus. Right: Line‐scan quantification of the nuclear envelope (NE) to nucleoplasm ratio of endogenous Nup62 in control (non‐neuronal) cells, versus neurons with ectopically expressed *rab‐*3p::Nup98::mNeonGreen. Error bars represent 95% CI for *n* > 12 nuclei (biological replicates).Top: Representative confocal micrographs showing localization of Nup62::wrmScarlet versus CRISPR‐tagged endogenous mNeonGreen::Nup98 in the tail of a Day 1 adult. Gray dashed lines indicate the boundary of the tail. Bottom: Representative confocal micrographs showing localization of Nup62 versus Nup98 at a single nucleus.Left: Representative images of Day 1 adults expressing no transgene (control) or the Nup98::mNeonGreen transgene driven by the pan‐neuronal *rab‐3* promoter. The control animal shows the wild‐type sinusoidal posture, whereas the transgenic animal exhibits an uncoordinated posture. Right: Graph showing the number of bends/minute during a swim test for Day 1 adults expressing no transgene (control) or the *rab‐3p*::Nup98::mNeonGreen transgene. Error bars represent 95% CI for *n* > 11 worms (biological replicates). Data information: ****P < 0.0001. Significance was determined using an unpaired *t*‐test. All the images in this figure are maximum intensity projections, with the exception of panels A and B (bottom) which are single focal planes. Scale bars = 10 μm (panels A and B, top) or 2 μm (panels A and B, bottom). Source data are available online for this figure.

We only detected Nup foci in somatic cells of aged hermaphrodites and *ogt* mutants (see Figs 4C and D, and 6D). To determine whether Nup condensation might be detrimental in somatic cells, we used the neuron‐specific *rab‐3* promoter to overexpress Nup98::mNeonGreen, a highly cohesive FG‐Nup that interacts with multiple structured Nups (Schmidt & Görlich, 2016; Onischenko *et al*, 2017). Unlike Nup98 expressed from its endogenous locus (Appendix Fig S8A), overexpressed Nup98 readily formed abundant cytoplasmic foci and localized to the NE at low levels (Appendix Fig S8B). Remarkably, the ectopic Nup98 foci recruited endogenous Nup62, resulting in partial depletion of Nup62 from the NE (Fig 7A). In control, non‐neuronal cells that did not express the Nup98 transgene, Nup62 localized to the NE as in wild‐type animals (Fig 7B). Consistent with disrupted nuclear transport in the Nup98 overexpressing neurons, the nuclear protein TDP‐43 was mislocalized to the cytoplasm (Appendix Fig S8C). Strikingly, *rab‐3p*::Nup98 animals had shorter lifespans (Appendix Fig S8D) and appeared uncoordinated (barely moving) on plates or in liquid media (Fig 7C, Movies EV3 and EV4), consistent with neuronal dysfunction and paralysis (Dimitriadi & Hart, 2010). We obtained similar results with an independently generated transgenic animal with *N*‐terminally tagged mNeonGreen::Nup98 expressed using the *rab‐3* promoter (Appendix Fig S8E–G, Movies EV5 and EV6). We conclude that uncontrolled Nup condensation in post‐mitotic neurons is toxic and leads to cellular dysfunction.

## Discussion

Cytoplasmic Nup foci have been observed in oocytes, yeast, and in many cell types in culture (Cordes *et al*, 1996; Colombi *et al*, 2013; Raghunayakula *et al*, 2015; Ren *et al*, 2019). In this study, we report the systematic examination of the incidence of Nup foci across all tissues in an intact animal. We find that Nup foci are rare in healthy animals and arise only in cells where cytoplasmic Nup concentration is highest: gametes and early embryos. Although Nup condensates appear prominent when observed by fluorescence microscopy, in growing oocytes and embryos they account for less than 3% of total cellular Nups and consist primarily of highly cohesive FG‐Nups. The vast majority of FG‐Nups are stored as soluble molecules in the cytoplasm whose condensation is actively suppressed by multiple mechanisms. Stress and aging promote FG‐Nup condensation which can be toxic in post‐mitotic cells if uncontrolled. Our findings do not support an essential role for Nup foci in pore assembly in *C. elegans* and instead we propose that Nup foci are non‐functional byproducts of the natural tendency of FG‐Nups to condense.

### Cytoplasmic Nup foci arise by condensation of FG‐Nups and their binding partners

Several lines of evidence suggest that condensation of FG‐Nups underlies Nup foci assembly. First, Nup foci in growing oocytes primarily consist of FG‐Nups and their binding partners and lack nucleoplasmic Nups as well as Nups essential for pore assembly (inner ring complex and transmembrane Nups). Second, Nup foci display heterogeneous Nup stoichiometry and rarely colocalize with membranes. Finally, consistent with condensation, a concentration‐dependent assembly mechanism, depletion and overexpression of individual FG‐Nups eliminate and enhance, respectively, foci formation. Together these observations suggest that Nup foci are not structured pre‐pore assemblies, but are condensates scaffolded by cohesive FG‐Nups, including Nup62, Nup98, Nup214, and Nup358, and their binding partner Nup88.

Consistent with our findings, a recent systematic survey in HEK293T cells revealed that cytoplasm‐facing FG‐Nups and their binding partners accumulate in cytoplasmic foci, but Nup153, which faces the nucleoplasm, does not (Cho *et al*, 2022). Other studies in HeLa and Cos7 cells have also documented that most Nup foci do not colocalize with membranes (Ren *et al*, 2019; Agote‐Aran *et al*, 2020). Similarly, Nup foci in yeast cells contain multiple FG‐Nups but lack transmembrane or inner ring complex Nups (Colombi *et al*, 2013). In agreement with this study, we found that the FG‐Nup Nup214 forms hexanediol‐sensitive foci in yeast cells, but the nucleoplasm‐facing Nups Nup50 and Nup153 do not (Appendix Fig S8H). Together, these studies suggest that, as we propose here for *C. elegans*, many reported Nup foci likely correspond to FG‐Nup condensates rather than pre‐assembled pore complexes.

We suggest that Nup condensates arise whenever the concentration of FG‐Nups exceeds the solubility threshold in the cytoplasm. Consistent with this hypothesis, depletion of scaffold nucleoporins that liberate FG‐Nups enhance foci formation in *C. elegans* oocytes (Fig 3, Appendix Fig S3), yeast (Makio *et al*, 2009), and HeLa cells (Raghunayakula *et al*, 2015). Similarly, intranuclear Nup assemblies termed GLFG bodies were reported in HeLa cell lines with elevated levels of Nup98, a highly cohesive FG‐Nup (Griffis *et al*, 2002; Morchoisne‐Bolhy *et al*, 2015). Our findings indicate that, even under conditions where FG‐Nups exceed their solubility limit by a small margin, they from bright foci easily visible by standard microscopy techniques.

### Phosphorylation, GlcNAcylation, and CRM1‐mediated chaperoning activity suppress Nup condensation

What keeps most FG‐Nups soluble in the cytoplasm? Our findings suggest that the solubility limit of FG‐Nups in the cytoplasm depends on several factors and oscillates with the cell cycle, peaking during M phase. The same kinases that drive nuclear pore complex disassembly during M phase (PLK1 and CDK1) appear to also promote Nup solubility in the cytoplasm during interphase. Although we did not directly monitor Nup phosphorylation, PLK1 and CDK1 have been well‐characterized as kinases that directly phosphorylate Nups to drive nuclear pore disassembly (Chase *et al*, 2000; De Souza *et al*, 2004; Onischenko *et al*, 2005; Laurell *et al*, 2011; Rahman *et al*, 2015; Linder *et al*, 2017; Martino *et al*, 2017). Consistent with phosphorylation promoting Nup solubility, cellular fractionation experiments have shown that soluble Nups are more highly phosphorylated than Nups in pore complexes (Onischenko *et al*, 2004). Other kinases implicated in Nup phosphorylation and pore complex disassembly, including NIMA and DYRK kinases (De Souza *et al*, 2004; Laurell *et al*, 2011; Wippich *et al*, 2013), could also contribute to Nup solubility.

Consistent with prior findings showing that O‐GlcNAcylated FG‐Nups are less prone to condensation *in vitro* (Labokha *et al*, 2012; Schmidt & Görlich, 2015), our observations also suggest that O‐GlcNAcylation contributes to Nup solubility in oocytes, embryos, and somatic cells. Numerous studies have reported a protective role for O‐GlcNAcylation in neurodegenerative disease (reviewed in Lee *et al*, 2021), raising the possibility that this modification plays a key role in solubilizing certain aggregation‐prone proteins. A separate study found that O‐GlcNAcylation promotes condensation of stress granules and P‐bodies (Ohn *et al*, 2008), indicating that the solubilizing effect of O‐GlcNAcylation is likely protein‐ and context‐dependent.

Finally, we find that the nuclear export factor CRM1 also contributes to Nup solubility. Structural analyses of CRM1/Nup214 complexes reveal that hydrophobic patches on the surface of CRM1 make extensive contacts with Nup214 FG domains (Port *et al*, 2015). CRM1 generates high‐affinity interactions with both Nup214 and Nup358 that are significantly stronger than the weak, transient interactions characteristic of most Nup/NTR pairs (Port *et al*, 2015; Ritterhoff *et al*, 2016; Tan *et al*, 2018). Both Nup214 and Nup358 are required for Nup foci formation and therefore neutralization of these proteins by CRM1 is predicted to reduce Nup foci formation. Interestingly, loss of another transport factor, transportin, did not affect Nup solubility in *C. elegans* oocytes, suggesting that not all NTRs play a significant role in promoting Nup solubility.

### Nup foci are not required for nuclear pore function or viability in *C. elegans*

During *Drosophila* oogenesis, Nup condensates mature into annulate lamellae (Hampoelz *et al*, 2019b), endoplasmic reticulum‐derived membranous structures that contain pore‐like complexes (Cordes *et al*, 1996; Miller & Forbes, 2000). Annulate lamellae have been observed in arrested oocytes of unmated *C. elegans* females (Patterson *et al*, 2011), where we find that Nup foci associate with the endoplasmic reticulum at a higher frequency, accumulate a greater proportion of FG‐Nups, and recruit additional Nups not present in foci of growing oocytes. It is possible, therefore, that, as reported for *Drosophila*, Nup foci have the potential to evolve into annulate lamellae in *C. elegans*, but this possibility will require further investigation.

In *Drosophila*, annulate lamellae assembled in oocytes have been proposed to fuel the rapid expansion of nuclear membranes in embryos by directly inserting into nuclear membranes during interphase (Hampoelz *et al*, 2016). Annulate lamellae have not been observed in *C. elegans* embryos (Pitt *et al*, 2000) and we find that all Nup assemblies dissolve during the oocyte‐to‐embryo transition. In embryos, Nup foci re‐appear and dissolve again with every M phase, in synchrony with the disassembly of nuclear pores at the NE. Annulate lamellae assembled in oocytes, therefore, are unlikely to be a source of pre‐assembled pores for embryos in *C. elegans*. Furthermore, we have identified two mutants, *nup214∆* and *nup88∆*, that severely reduce the incidence of Nup foci in oocytes and embryos yet assemble functional nuclear pores during embryogenesis and are viable. We consider it unlikely, therefore, that Nup foci contribute significantly to nuclear pore assembly in *C. elegans*.

If so, why do Nup foci assemble in *C. elegans* oocytes? We considered the possibility that Nup foci sequester damaged Nups that must be removed from the soluble pool before embryogenesis, which may be particularly important during oocyte arrest (Bohnert & Kenyon, 2017). This possibility, however, appears unlikely as the ~10% of Nups in foci in arrested oocytes return to the soluble pool without any loss upon oocyte maturation. Additionally, *nup214∆* arrested oocytes, which have reduced Nup foci, have higher Nup levels at the nuclear rim (compared with wild‐type) and produce fully viable embryos, suggesting that, when not induced to form foci, excess Nups can assemble into nuclear pores at the nuclear periphery. Another possibility is that Nup foci serve a role unrelated to nuclear pore formation that becomes essential under conditions not tested in this study. Prior studies have noted that Nup foci assemble near RNP granules (Pitt *et al*, 2000; Jud *et al*, 2007; Sheth *et al*, 2010; Patterson *et al*, 2011; Sahoo *et al*, 2017), suggesting that Nup foci may contribute to RNA homeostasis, but this possibility remains to be tested. A final possibility is that Nup foci serve no function and arise simply as the inevitable consequence of the high concentration of FG‐Nups needed for embryogenesis, which transiently saturates the cytoplasm of oocytes and early embryos.

### Uncontrolled Nup condensation can be toxic in post‐mitotic cells

Overexpression of Nup98 in neurons was sufficient to assemble ectopic Nup foci and cause paralysis, suggesting that uncontrolled Nup condensation in somatic cells is potentially toxic. We speculate that neuronal dysfunction arose as a consequence of impaired nucleocytoplasmic transport due to recruitment of endogenous Nups to the ectopic foci. Our findings are consistent with recent studies reporting that cytoplasmic FG‐Nups drive aggregation of TDP‐43 in both ALS/FTLD and following traumatic brain injury (Anderson *et al*, 2021; Gleixner *et al*, 2022). Several other studies have linked Nup condensation to stress and disease, including: (i) Nup accumulation in stress granules (Zhang *et al*, 2018; Agote‐Aran *et al*, 2020), (ii) aberrant condensation of Nup98 and Nup214 fusion proteins driving oncogenic transformation in certain leukemias (Zhou & Yang, 2014; Terlecki‐Zaniewicz *et al*, 2021; Chandra *et al*, 2022), (iii) the formation of NE associated Nup condensates in models of DYT1 dystonia (Prophet *et al*, 2022), and (iv) the presence of Nups in pathological inclusions in primary patient samples and models of neurodegenerative disease (reviewed in Fallini *et al*, 2020; Hutten & Dormann, 2020; Chandra & Lusk, 2022).

The deleterious effects of Nup condensation are likely context dependent. In arrested oocytes, Nup condensation increases ~14‐fold over growing oocytes, yet is not damaging as the majority of arrested oocytes go on to form viable embryos when fertilized (Jud *et al*, 2008; Patterson *et al*, 2011). Pore complexes and Nup condensates in oocytes and embryos fully disassemble during M phase, allowing for a cycle of “renewal” with each cell division. Nup condensation may only be dangerous in post‐mitotic cells that lack M phase‐specific Nup solubilizers and where certain Nups are naturally long‐lived (D'Angelo *et al*, 2009; Toyama *et al*, 2013). We suggest that post‐mitotic cells avoid Nup condensation by maintaining low levels of cytoplasmic Nups and high levels of solubilizing modifications. Indeed, we observed that Nup foci in the somatic tissues of aged adults become more prominent in *ogt* mutants lacking O‐GlcNAcylation. We do not know the origin of Nup foci in aged cells, but they may be linked to the progressive decline in proteostasis and nuclear ‘leakiness’ that initiates during *C. elegans* adulthood (Herndon *et al*, 2002; Ben‐Zvi *et al*, 2009; D'Angelo *et al*, 2009). *C. elegans* oocytes and embryos, which naturally accumulate and clear Nup condensates, offer a powerful model system to explore possible mechanisms to prevent or reverse Nup condensation during aging.

## Materials and Methods

### *C. elegans* and yeast strains and culture

*C. elegans* were cultured using standard methods (Brenner, 1974). Briefly, worms were maintained at 20°C on normal nematode growth media (NNGM) plates (IPM Scientific Inc. cat # 11006‐548) seeded with OP50 bacteria. We have found that Nup solubility is highly influenced by multiple factors including animal age: for all Nups tested the number and size of foci in growing oocytes increased significantly between Days 1 and 2 of adulthood (see Appendix Fig S1D). Therefore, for all experiments worms were synchronized as Day 1 or 2 adults using vulval morphology to stage L4 larvae. The age of animals used for each experiment is indicated in figures and legends.

Endogenous *npp‐21* (TPR) was tagged with GFP using CRISPR/Cas9‐mediated genome editing as previously described (Arribere *et al*, 2014). Endogenous *npp‐24* (Nup88) and *npp‐2* (Nup85) were tagged with G>F>P using SapTrap CRISPR/Cas9 gene modification as previously described (Schwartz & Jorgensen, 2016). G>F>P contains Frt sites in introns 1 and 2 of GFP that enable FLP‐mediated, conditional knockout; in the absence of FLP, the construct behaves as normal GFP. To generate a permanent *npp‐24* knockout, recombination was induced in the germline (Macías‐León & Askjaer, 2018) followed by selection of progeny in which the second GFP exon was excised from both alleles. This strategy phenocopies complete gene removal (Fragoso‐Luna *et al*, 2023). Endogenous *npp‐19* (Nup35) was tagged with G>F>P based on protocols for nested CRISPR (Vicencio *et al*, 2019) and ‘hybrid’ partially single‐stranded DNA donors (Dokshin *et al*, 2018). All other endogenous edits were performed using CRISPR/Cas9‐mediated genome editing as described previously (Paix *et al*, 2017). Transgenic Nup214 and Nup98 strains (JH4119, JH4204, JH4205, and JH4395) were generated using SapTrap cloned vectors as previously described (Fan *et al*, 2020). Standard crosses were used to generate strains with multiple genomic edits. All strains used or generated in this study are described in Appendix Table S1.

Yeast strains were generated using homologous recombination of PCR‐amplified cassettes (Longtine *et al*, 1998). Endogenous *NUP159* (Nup214), *NUP60* (Nup153), and *NUP2* (Nup50) were tagged by amplifying the *mNeonGreen::HIS3* cassette from pFA6a‐mNeonGreen::HIS3 (Thomas *et al*, 2019) using primers with homology to the C‐termini (without the stop codon) and downstream regions of the genes. Yeast strains generated in this study are described in Appendix Table S1.

### RNAi

RNAi was performed by feeding (Timmons & Fire, 1998). RNAi vectors were obtained from the Ahringer or Open Biosystems libraries and sequence verified, or alternatively cloned from *C. elegans* cDNA and inserted into the T777T enhanced RNAi vector (Addgene cat # 113082). RNAi feeding vectors were freshly transformed into HT115 bacteria, grown to log phase in LB + 100 μg/ml ampicillin at 37°C, induced with 5 mM IPTG for 45 min, and plated on RNAi plates (50 μg/ml Carb, 1 mM IPTG; IPM Scientific Inc. cat # 11006‐529). Seeded plates were allowed to dry overnight at RT before adding L4 larvae or Day 1 adults. For depletion of Nup98 (Fig 3B, Appendix Fig S3C), RNAi feeding was performed for 6 h at 25°C; partial depletion was used to minimize cytological defects caused by loss of Nup98. For all other experiments, RNAi feeding was performed for 18–24 h at 25°C. For all experiments, control worms were fed HT115 bacteria transformed with the corresponding L4440 or T777T empty vector.

### Immunofluorescence

For immunostaining of embryos, gravid adults were placed into 7 μl of M9 media on a poly‐L‐lysine coated slide and compressed with a coverslip to extrude embryos. For immunostaining of oocytes, staged adults were dissected on poly‐L‐lysine slides to extrude the germline, and a coverslip was placed gently on top. In both cases, slides were immediately frozen on aluminum blocks pre‐chilled with dry ice. After > 5 min, coverslips were removed to permeabilize embryos (freeze‐cracking), and slides were fixed > 24 h in pre‐chilled MeOH at −20°C. Slides were then incubated in pre‐chilled acetone for 10 min at −20°C, and blocked in PBS–T (PBS, 0.1% Triton X‐100, 0.1% BSA) for > 30 min at RT. Slides were then incubated overnight in primary antibody in a humid chamber at 4°C. Slides were washed 3 × 10 min in PBS‐T at RT, incubated in secondary antibody for 2 h in a humid chamber at RT, and washed 3 × 10 min in PBS–T at RT. Slides were then washed 1x in PBS before being mounted using Prolong Glass Antifade Mountant with NucBlue (Thermo Fisher cat # P36981). Primary antibodies were diluted as follows: mAb414 (1:1,000; Biolegend cat # 902907), anti‐Nup358 (1:250; Novus Biologicals cat # 48610002), anti‐Nup50 (1:250, Novus Biologicals cat # 48590002), anti‐GlcNAc RL2 (1:100; Invitrogen cat # MA1‐072), anti‐Nup96 (1:250, Ródenas *et al*, 2012), anti‐Nup153 (1:250, Galy *et al*, 2003), anti‐OLLAS‐L2 (1:50, Novus Biologicals cat # NBP1‐06713). Secondary antibodies were diluted as follows: Cy3 Donkey anti‐Mouse IgG (1:200; Jackson cat # 715‐165‐151), AlexaFluor 488 Goat anti‐Rabbit IgG (1:200; Invitrogen cat # A‐11034), AlexaFluor 568 Goat anti‐Rabbit IgG (1:200; Invitrogen cat # A‐11011), Alexa Fluor 488 Goat anti‐Rat IgG (1:200; Invitrogen cat # A‐11006), AlexaFluor 488 anti‐GFP (1:500; Invitrogen cat # A‐21311).

### LMB and HXD treatment, HaloTag and Hoechst labeling, and heat stress

For CRM1 inhibition, leptomycin B (LMB; Sigma cat # L2913) was diluted in OP50 bacteria to a final concentration of 500 ng/ml and seeded on NNGM plates. 10‐20 Day 1 adults were transferred to LMB or control vehicle plates and incubated at 20°C for 4 h prior to imaging. For treatment of embryos with 1,6‐hexanediol (HXD, Acros Organics cat # 629‐11‐8), L4 larvae were fed *ptr‐2* RNAi for 18–24 h at 20°C. Embryos depleted of PTR‐2, which permeabilizes the eggshell to allow for HXD treatment, were dissected into L‐15 media (Thermo Fisher cat # 21083027) containing 2% HXD and immediately imaged. For HXD treatment of yeast, log‐phase yeast were pelleted, re‐suspended in media containing 5% HXD, and allowed to grow for 10 min at 30°C prior to imaging.

For HaloTag labeling, Janelia Fluor 646 HaloTag Ligand (Promega cat # GA1121) was diluted in OP50 bacteria to a final concentration of 25 μg/ml and seeded on NNGM plates. 10–20 L4 larvae or Day 1 adults were added and incubated without light at 20°C for 16–20 h prior to imaging. For Hoechst staining, Hoechst 33342 dye (Thermo Fisher cat # 62249) was diluted in OP50 bacteria to a final concentration of 200 μM and seeded on NNGM plates. 10–20 L4 larvae were added and incubated without light at 20°C for 16–20 h prior to imaging.

To induce heat stress, animals were grown at 20°C then transferred to pre‐warmed NNGM plates at 30°C for 20 min prior to imaging at room temperature or processing for immunofluorescence.

### Embryonic viability and lifespan analysis

To measure embryonic viability of the *nup214Δ* mutant (Appendix Fig S5G), six Day 1 adults were transferred to six NNGM plates (36 animals total) and allowed to lay embryos for 1 h at 20°C. To measure embryonic viability of the *nup88Δ* mutant (Appendix Fig S5J), two Day 1 adults were transferred to six plates (12 animals total) and allowed to lay embryos for 5 h at 20°C. Adults were then removed and the number of embryos on each plate was counted. For the *nup214Δ* mutant, embryos were allowed to hatch and the number of adults on each plate was counted after 3 days at 20°C. For the *nup88Δ* mutant, the number of unhatched embryos was counted after 1 day at 20°C. Viability counts were repeated in at least two independent experiments, and embryonic viability was measured as the number of surviving adults or hatched larvae divided by the original number of embryos counted in each experiment.

To measure embryonic viability of arrested oocytes following mating (Appendix Fig S5M), *fog‐2*(*q71*) female L4 larvae were incubated overnight at 20°C in the absence of males. Individual Day 1 adult females with arrested oocytes were then mated with 5–7 males on a small patch of bacteria. Matings were monitored closely, and the adults removed once 3–10 embryos were laid. The number of embryos on each plate was counted, embryos were allowed to hatch, and the number of adults on each plate was counted after 3 days at 20°C. Viability counts were repeated in three independent experiments, and embryonic viability was measured as the number of surviving adults divided by the original number of embryos laid. Prior studies have reported that > 90% of arrested oocytes produce viable embryos (Jud *et al*, 2008; Patterson *et al*, 2011), whereas we found that ~70% of arrested oocytes gave rise to viable embryos (Appendix Fig S5M). These prior studies measured the viability of all oocytes accumulated prior to mating (~20 per gonad arm), whereas we measured the viability of the first few oocytes ovulated following mating (~2–5 per gonad arm). This difference may explain the comparatively lower viability observed in our experiments.

To measure adult lifespan, 75 Day 1 adults were transferred to five NNGM plates (15 animals per plate) and incubated at 20°C. Worms were scored daily and considered to be dead if they failed to move when prodded. Worms were transferred every 2 days to avoid progeny, and any worms that crawled off the plates were censored from analysis.

### Swimming assay

To measure swimming behavior, 5–10 Day 1 adults were transferred to a 33 mm culture dish (MatTek cat # P35G‐1.5‐14‐C) containing 400 μl M9 media and immediately filmed using an Axiocam 208 color camera (Zeiss) mounted on a Stemi 508 Stereo Microscope (Zeiss). Swimming assays were performed at RT (~22°C). Movies were exported to ImageJ, and the number of body bends per minute was counted manually.

### Imaging

For live imaging of germlines and somatic tissues, 5 staged adults were transferred to the middle well of a 3‐chambered slide (Thermo Fisher cat # 30‐2066A) in 10 μl of L‐15 media with 1 mM levamisole. 20 μm polystyrene beads (Bangs Laboratories Inc. cat # PS07003) were then added to support a coverslip (Marienfeld cat # 0107052). Germlines were imaged using an inverted Zeiss Axio Observer with CSU‐W1 SoRa spinning disk scan head (Yokogawa), 1×/2.8×/4× relay lens (Yokogawa), and an iXon Life 888 EMCCD camera (Andor) controlled by Slidebook 6.0 software (Intelligent Imaging Innovations). To image germlines or somatic cells, a 20 μm Z stack (1 μm step size) was captured using a 63× objective (Zeiss) with the 1× relay lens. For high resolution images of oocytes, 3 μm Z stacks (0.1 μm step size) were acquired using the 63× objective with the 2.8× relay lens. As germline condensates are highly sensitive to imaging‐induced stress (Elaswad *et al*, 2022), care was taken to avoid compression of germlines, and all animals were imaged only once and maintained on the slide for <5 min. To image entire worms (Appendix Fig S8A, B, and E), an 80 μm Z stack (1 μm step size) was captured using a 10× objective (Zeiss) with the 1x relay lens.

For imaging arrested oocytes and newly fertilized zygotes following mating (Fig 5A), *fog‐2*(*q71*) female L4 larvae were incubated overnight at 20°C in the absence of males. Day 1 adult females with arrested oocytes were then mated or not with an abundance of males at 20°C for 1.5 h prior to imaging.

For live imaging of embryos, five young adults were transferred to 10 μl of L‐15 media on a coverslip and dissected to release embryos. 20 μm polystyrene beads were then added to prevent compression, and the coverslip was inverted onto a microscope slide (Thermo Fisher cat # 12‐550‐403). Embryos were imaged as 15 μm Z stacks (1 μm step size), captured using the 63× objective with the 2.8× relay lens. For imaging fixed germlines and embryos, prepared slides were imaged as 15 μm Z stacks (0.5 μm step size), captured using the 63× objective with the 2.8× relay lens.

For live imaging of yeast, cells were grown overnight in synthetic dropout media (Thermo Fisher cat # DF0919‐15‐3) at 30°C and imaged in log‐phase (OD_600_ of ~0.5) at room temperature. Yeast were imaged as 6 μm Z stacks (0.5 μm step size), captured using the 63× objective with the 2.8× relay lens.

Images were exported from Slidebook software and further analyzed using ImageJ or Imaris image analysis software. For presentation in figures, images were processed using ImageJ, adjusting only the minimum/maximum brightness levels for clarity with identical leveling between all images in a figure panel. Images presented in figures are maximum intensity projections (10 μm for germlines, 15 μm for embryos, 6 μm for yeast) or single focal planes as indicated in the legends.

### Image quantification

The overlap of GFP or mNeonGreen‐tagged Nups with Nup62::wrmScarlet (Fig 2C) was measured using single focal planes exported to ImageJ. The Nup62::wrmScarlet micrograph was used to create a mask defining the NE as well as cytoplasmic foci as individual regions of interest (ROIs). This mask was then applied to both the GFP/mNeonGreen Nup micrograph as well as the Nup62::wrmScarlet micrograph and the integrated density was measured within each ROI. To control for cytoplasmic background, the average cytoplasmic signal for the GFP/mNeonGreen Nup was multiplied by the area of each ROI, and the resulting value subtracted from integrated density for the GFP/mNeonGreen Nup. Background normalized GFP/mNeonGreen Nup values were divided by Nup62::wrmScarlet values to obtain the ratio of GFP/mNeonGreen Nup to Nup62::wrmScalet at each ROI.

To quantify the overlap of GFP::Nup88 with membranes (Fig 2D, Appendix Fig S4C), Z stacks of oocytes expressing GFP::Nup88 and the HaloTag::HDEL reporter were manually scored into three categories: 1. Complete overlap (the entire Nup88 focus overlapped with HaloTag::HDEL); 2. Partial overlap (the Nup88 focus partially overlapped or was directly adjacent to HaloTag::HDEL); 3. No overlap (the Nup88 focus did not directly contract membranes marked by HaloTag::HDEL).

To quantify the distribution of Nups in oocytes as well as total expression, Z stacks were exported to Imaris image analysis software. The ‘Surface’ tool was first used to isolate the ‐3 and ‐4 oocytes from each germline (Appendix Fig S3A). For each pair of ‐3 and ‐4 oocytes, the Surface tool was then used to isolate both nuclei and the “Spot” tool was used to isolate cytoplasmic foci. The percent of Nup present at the NE/nucleoplasm was measured as the intensity sum for both nuclei divided by the total intensity sum of the oocytes. Similarly, the percent of Nup present in foci was measured as the intensity sum for all foci divided by the total intensity sum of the oocytes. Finally, the percent soluble Nup was defined as 100% minus the percentage of Nup in both nuclei and foci. Total Nup expression was measured as the intensity sum of the ‐3 and ‐4 oocytes normalized to volume. To control for autofluorescent background in all measurements, staged animals lacking fluorescent tags were imaged using identical imaging settings. The average intensity sum per volume was calculated for the ‐3 and ‐4 oocytes of germlines lacking fluorescent tags and subtracted from the intensity sum measured for oocytes with tagged Nups. To measure the intensity of GFP::Nup35 per nuclear volume (Appendix Fig S5H), the intensity value for the ‐3 and ‐4 oocyte nuclei or 3 embryonic nuclei were divided by nuclear volume and the resulting values were averaged for each germline or embryo.

To quantify the distribution of Nups in embryos, Z stacks were exported to Imaris software. The Surface tool was used to isolate the entire embryo as well as all nuclei, and the Spot tool was used to isolate cytoplasmic foci. The percent of Nup at the NE/nucleoplasm or foci was measured as the intensity sum of all nuclei or foci divided by the total intensity sum of the embryo, respectively. The percent soluble Nup was defined as 100% minus the percentage of Nup in nuclei and foci. For all measurements, embryos lacking fluorescent tags were used to control for autofluorescent background as described for oocytes.

The Y complex component Nup85 localizes to meiotic chromosomes and a high percentage of Nup85 is present in the nucleoplasm. Therefore, line‐scan analysis was used to measure the amount of GFP::Nup85 at the NE (Fig 3B). Z stacks were exported to ImageJ and line traces were drawn to pass through the central plane of ‐3 and ‐4 oocyte nuclei as well as the image background. For each nucleus, the two peak values of the NE rim were averaged and normalized to the image background. Line‐scan analysis was also used to quantify depletion of endogenous Nup62::wrmScarlet from the NE in neurons expressing *rab‐3p*::Nup98::mNeonGreen (Fig 7A). Line traces were drawn to pass through the central plane of nuclei identified by Hoechst staining. For each nucleus, the two peak values for the NE rim were averaged and normalized to the average value of Nup62 in the nucleoplasm. To quantify the partitioning of TFEB::GFP, IBB_domain_::mNeonGreen, TDP‐43::wrmScarlet, CRM1::mNeonGreen, and G3BP::mCherry between the nucleus and cytoplasm (Fig 5E, Appendix Figs S5K and S7E) line traces were drawn to pass through the cytoplasm as well as the nucleoplasm and image background. Average intensity values for the nucleus and cytoplasm were background subtracted, then the value for the nucleus was divided by that of the cytoplasm.

To quantify cytoplasmic levels of mNeonGreen::Nup358 in oocytes versus somatic cells and embryos (Fig 1D), single focal planes captured from the same animal were exported to ImageJ. For each animal 3 ROIs in the ‐1 oocyte, somatic cell type of interest, or 4‐cell embryo cytoplasm were measured, averaged, and normalized to the image background. Values for the somatic cell or embryo cytoplasm were then normalized to that of the oocyte within the same animal.

To quantify foci formation in somatic tissues of aged animals (Fig 6D), Z stacks of Day 4 adult heads expressing GFP::Nup88 were manually scored into three categories: 1. None (no ectopic GFP::Nup88 foci were present); 2. Mild (several small GFP::Nup88 foci were observed); 3. Moderate (many large GFP::Nup88 foci were present).

### Statistical analysis

All the statistical tests were performed using GraphPad Prism 9.2.0 software. For comparison of three or more groups, significance was determined using a one‐way ANOVA. For comparison of two groups, significance was determined using an unpaired *t*‐test. In all figures, error bars represent 95% confidence intervals (CIs). For all figures, ns indicates not significant; *P < 0.05; **P < 0.01; ***P < 0.001; ****P < 0.0001.

## Author contributions

**Laura Thomas:** Conceptualization; formal analysis; validation; investigation; visualization; writing – original draft. **Basma Taleb Ismail:** Formal analysis; validation; investigation; visualization. **Peter Askjaer:** Supervision; funding acquisition; writing – review and editing. **Geraldine Seydoux:** Conceptualization; supervision; funding acquisition; writing – original draft.

In addition to the CRediT author contributions listed above, the contributions in detail are:

Investigation: LT, BTI; Formal analysis: LT, BTI; Validation: LT, BTI; Visualization: LT, BTI; Conceptualization: LT, GS; Writing – original draft: LT, GS; Writing – review and editing: PA; Funding acquisition: GS, PA; Supervision: GS, PA.

## Disclosure and competing interests statement

The authors declare that they have no conflict of interest.

## Supporting information



Appendix S1

Movie EV1

Movie EV2

Movie EV3

Movie EV4

Movie EV5

Movie EV6

Source Data for Appendix

Source Data for Figure 1

Source Data for Figure 2

Source Data for Figure 3

Source Data for Figure 4

Source Data for Figure 5

Source Data for Figure 6

Source Data for Figure 7

## Data Availability

Original high resolution Z stacks for all images used in figures have been deposited in the BioImage Archive: accession number S‐BIAD651 (https://www.ebi.ac.uk/biostudies/BioImages/studies/S‐BIAD651?query=S‐BIAD651).
