# Supplementary material for: Nucleoporin foci are stress‐sensitive condensates dispensable for C. elegans nuclear pore assembly
Source: EMBO J. 2023 May 31;42(13):e112987. doi: 10.15252/embj.2022112987 (PMC10308366; doi:10.15252/embj.2022112987)
Supplement: Supplementary file 1 — Appendix S1 [file EMBJ-42-e112987-s009.pdf]

TABLE OF CONTENTS

Appendix Figures and Figure legends

Appendix Figure S1. Cytoplasmic Nup foci are unique to germ cells and early embryos……………………………………………………………………………………….2
Appendix Figure S2. FG-Nups and their binding partners form cytoplasmic foci in early embryos……………………………………………………………………………………….4
Appendix Figure S3. Nup foci are scaffolded by hydrophobic interactions……………….6
Appendix Figure S4. Nup foci in arrested oocytes enrich additional Nups and overlap more frequently with the endoplasmic reticulum…………………………………………….9
Appendix Figure S5. Robust Nup foci are not required for embryonic viability…………11
Appendix Figure S6. CDK1 and OGT are enriched at foci and promote Nup solubility..14
Appendix Figure S7. CRM1 and transportin are enriched in cytoplasmic Nup foci…….16
Appendix Figure S8. Ectopic Nup98 condensation in post-mitotic neurons is toxic……18
Appendix Table S1. *C. elegans* and yeast strains generated and used in this study….21
Appendix References………..……………………………………………………………..25

Appendix Figure S1

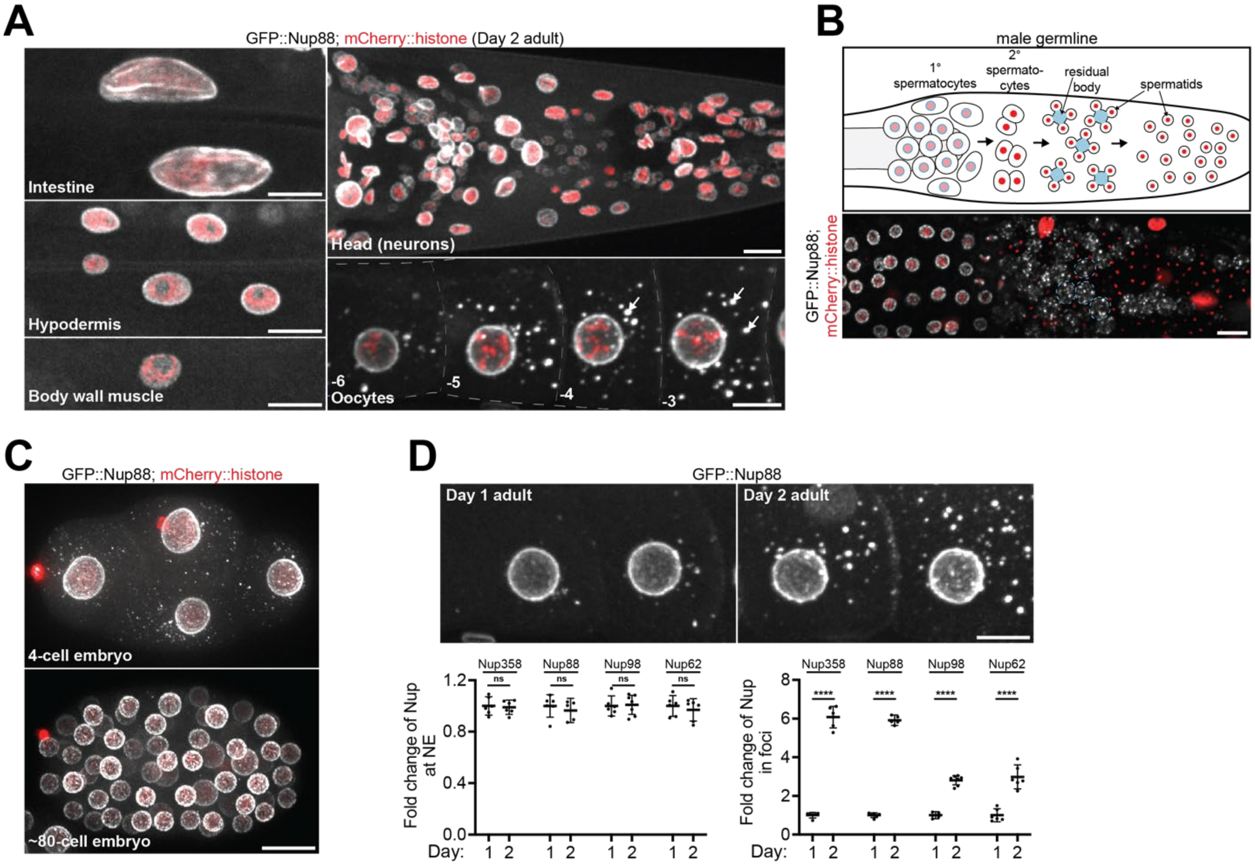

Appendix Figure S1. Cytoplasmic Nup foci are unique to germ cells and early embryos. A. Representative confocal micrographs of CRISPR-tagged GFP::Nup88 in the intestine, hypodermis, body wall muscle, head, and oocytes of Day 2 adult *C. elegans*. Nuclei are marked by a mCherry::histone transgene. White arrows denote cytoplasmic foci in oocytes. B. Top: Schematic depicting spermatogenesis in the *C. elegans* male germline. Following meiotic divisions spermatids bud from an anucleate residual body (denoted in blue); material within the residual body, including most Nups, is discarded. Bottom: Representative confocal micrograph showing GFP::Nup88 in the *C. elegans* male germline. Four residual bodies are outlined with a blue dashed line. C. Representative confocal micrographs showing GFP::Nup88 in interphase 4-cell versus ~80-cell embryos. D. Top: Representative confocal micrographs showing -3 and -4 oocytes of Day 1 versus Day 2 adults expressing GFP::Nup88. Left graph: Quantification of the total percent of each indicated Nup at the nuclear envelope

(NE)/nucleoplasm in Day 1 versus Day 2 adults. Values are normalized so that the average value for Day 1 adults = 1.0. Error bars represent 95% CI for n > 5 germlines (biological replicates). Right graph: Quantification of the total percent of each indicated Nup in foci in Day 1 versus Day 2 adults. Values are normalized so that the average value for Day 1 adults = 1.0. Error bars represent 95% CI for n > 5 germlines (biological replicates). Day 2 adult data is repeated from Figure 3A.

Data information: ****P<0.0001; ns, not significant. Significance was determined using an unpaired t-test. All images in this figure are maximum intensity projections. Scale bars = 10 µm.

Appendix Figure S2

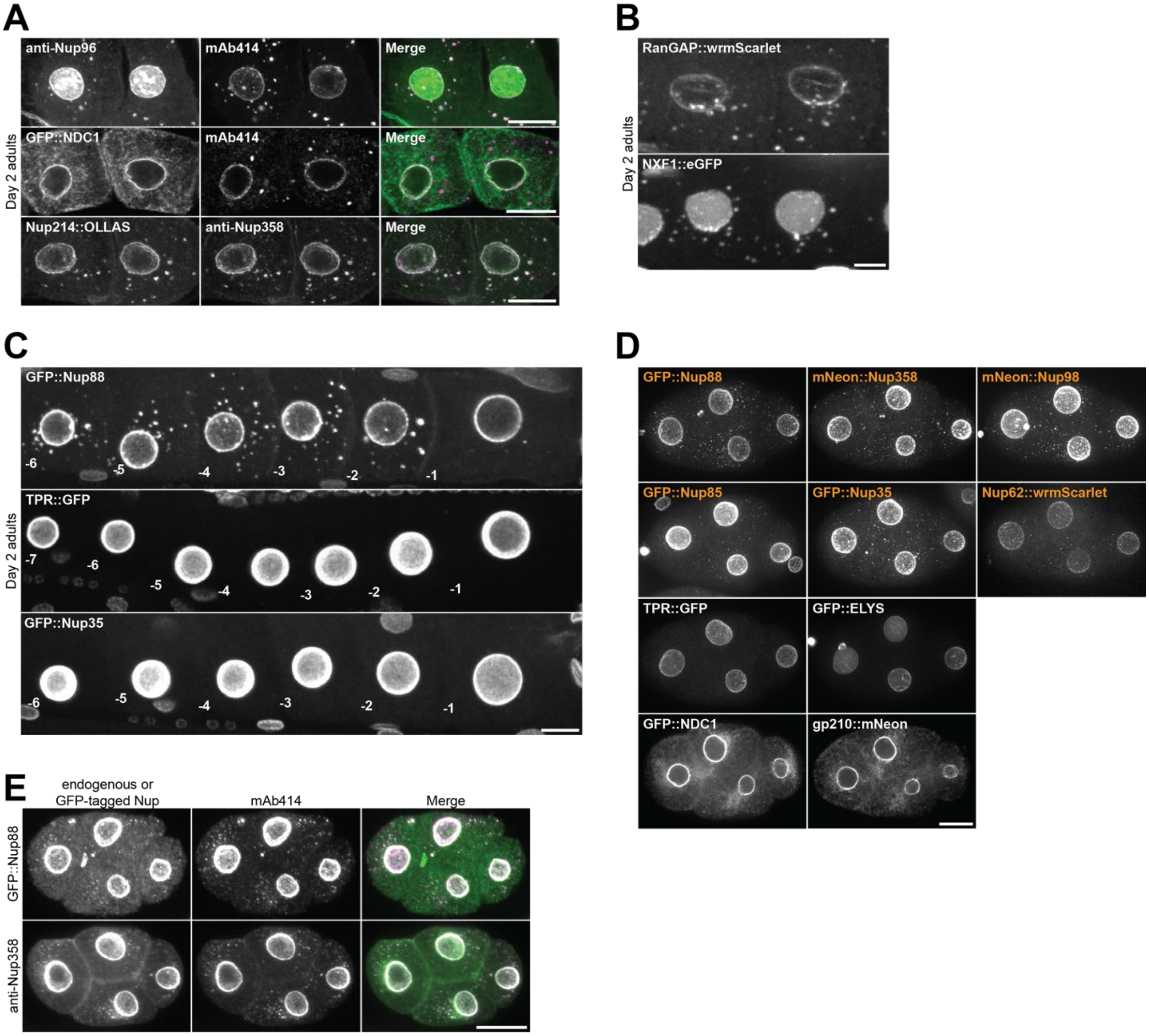

Appendix Figure S2. FG-Nups and their binding partners form cytoplasmic foci in early embryos. A. Top: Representative confocal micrographs depicting localization of endogenous Nup96 versus mAb414 in Day 2 adult oocytes. Middle: Representative confocal micrographs depicting localization of CRISPR-tagged GFP::NDC1 versus mAb414 in Day 2 adult oocytes. Bottom: Representative confocal micrographs showing localization of CRISPR-tagged Nup214::OLLAS versus endogenous Nup358 in Day 2 adult oocytes. B. Representative confocal micrographs of -3 and -4 oocytes from Day 2 adults expressing CRISPR-tagged RanGAP::wrmScarlet or transgenic NXF1::eGFP. C.

Representative confocal micrographs showing localization of CRISPR-tagged GFP::Nup88, TPR::GFP, and GFP::Nup35 in germlines (-1 to -6/-7 position oocytes) in Day 2 adults. D. Representative confocal micrographs showing interphase 4-cell embryos with each designated CRISPR-tagged Nup. Orange labels denote Nups that localize to cytoplasmic foci. E. Representative confocal micrographs depicting localization of GFP::Nup88 or endogenous Nup358 relative to mAb414 in 4-cell embryos.

Data information: All images in this figure are maximum intensity projections, with the exception of NDC1 with mAb414 (panel A) and NDC1 and gp210 (panel D) which are single focal planes. Scale bars = 10 µm.

Appendix Figure S3

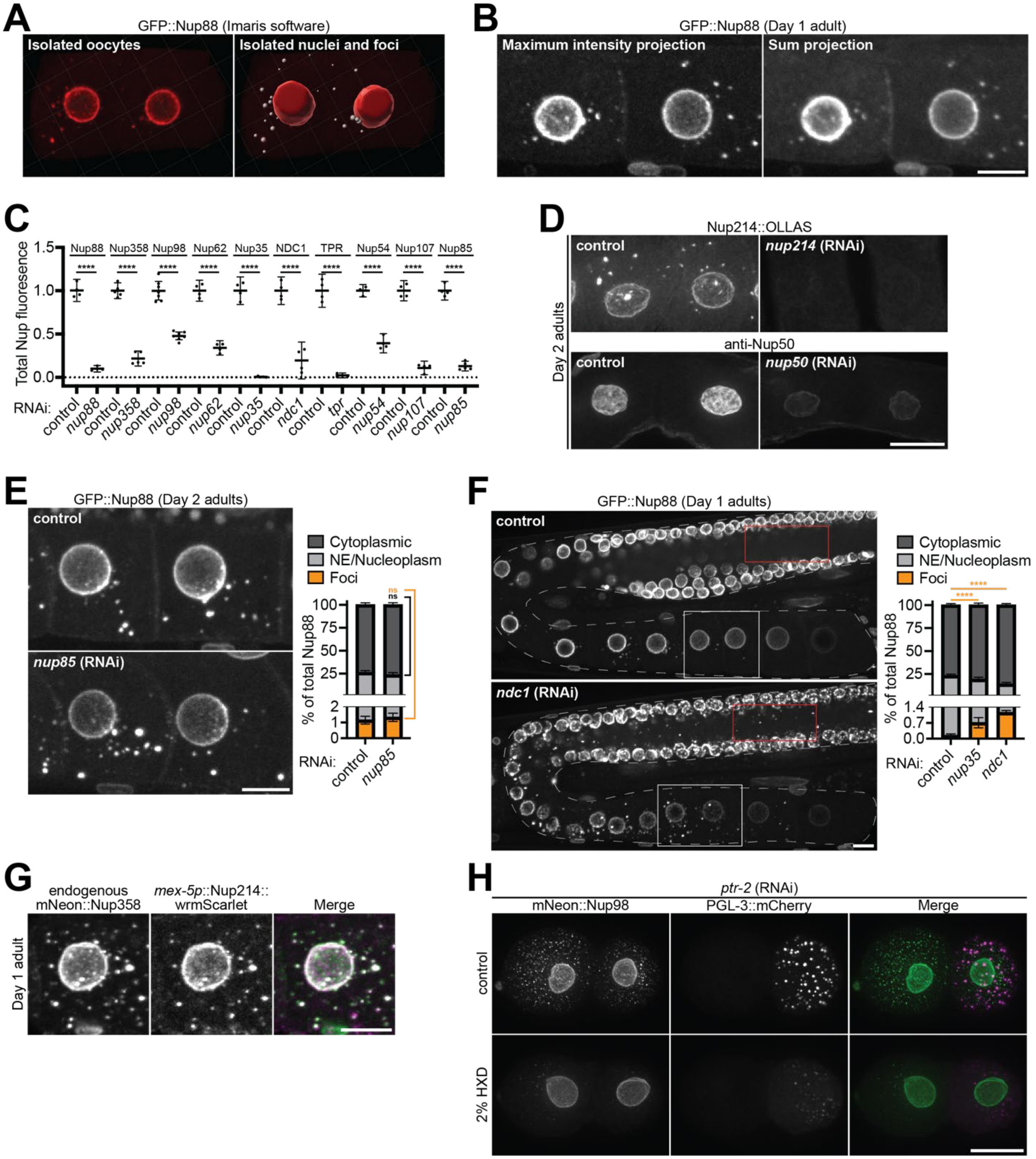

Appendix Figure S3. Nup foci are scaffolded by hydrophobic interactions. A. Images depicting the workflow used to measure the distribution of individual Nups in oocytes. Left: 3D reconstructions of -3 and -4 position oocytes were first isolated using

Imaris software and total Nup fluorescence was measured. Right: Nuclei (red surfaces) and foci (grey spots) were then isolated, and the amount of Nup fluorescence for each group was measured. Also see materials and methods. B. Representative confocal micrographs showing CRISPR-tagged GFP::Nup88 in the -3 and -4 oocytes of a Day 1 adult. The 10 um Z stack is shown as a maximum intensity projection (left) or a sum projection (right). C. Quantification of the extent of RNAi-mediated depletion of each indicated Nup. Values are normalized so that the average control measurement = 1.0. Error bars represent 95% CI for n > 4 germlines (biological replicates). D. Representative confocal micrographs showing depletion of CRISPR-tagged Nup214::OLLAS or endogenous Nup50 following *nup214* or *nup50* RNAi, respectively. E. Left: Representative confocal micrographs showing -3 and -4 oocytes of Day 2 adults with GFP::Nup88 as a marker for foci formation, with control RNAi or following depletion of Nup85. Right: Quantification of the distribution of GFP::Nup88 between the cytoplasm (soluble), nuclear envelope (NE)/nucleoplasm, and cytoplasmic foci in control oocytes versus those depleted of Nup85. Error bars represent 95% CI for n = 6 germlines (biological replicates). F. Left: Representative confocal micrographs of Day 1 adult germlines expressing GFP::Nup88 with control RNAi or following depletion of NDC1. Red boxes denote the syncytial cytoplasm; foci are absent in control germlines but accumulate following depletion of NDC1. White boxes indicate the -3 and -4 oocytes used for quantification. Right: Quantification of the distribution of GFP::Nup88 between the cytoplasm, NE/nucleoplasm, and cytoplasmic foci in control oocytes versus those depleted of Nup35 or NDC1. Error bars represent 95% CI for n > 5 germlines (biological replicates). G. Representative confocal micrographs showing localization of CRISPR-tagged endogenous mNeonGreen::Nup358 versus Nup214::wrmScarlet overexpressed using the *mex-5* promoter. H. Representative confocal micrographs showing CRISPR-tagged mNeonGreen::Nup98 and the P granule marker PGL-3::mCherry in 2-cell embryos treated or not with 2% 1,6-hexandiol (HXD) immediately prior to imaging. *ptr-2* RNAi was used to permeabilize the embryo eggshell to allow for HXD treatment.

Data information: ****P<0.0001; ns, not significant. For panel F significance was determined using a one-way ANOVA; for all other panels significance was determined

using an unpaired *t*-test. All images in this figure are maximum intensity projections. Scale bars = 10 µm.

Appendix Figure S4

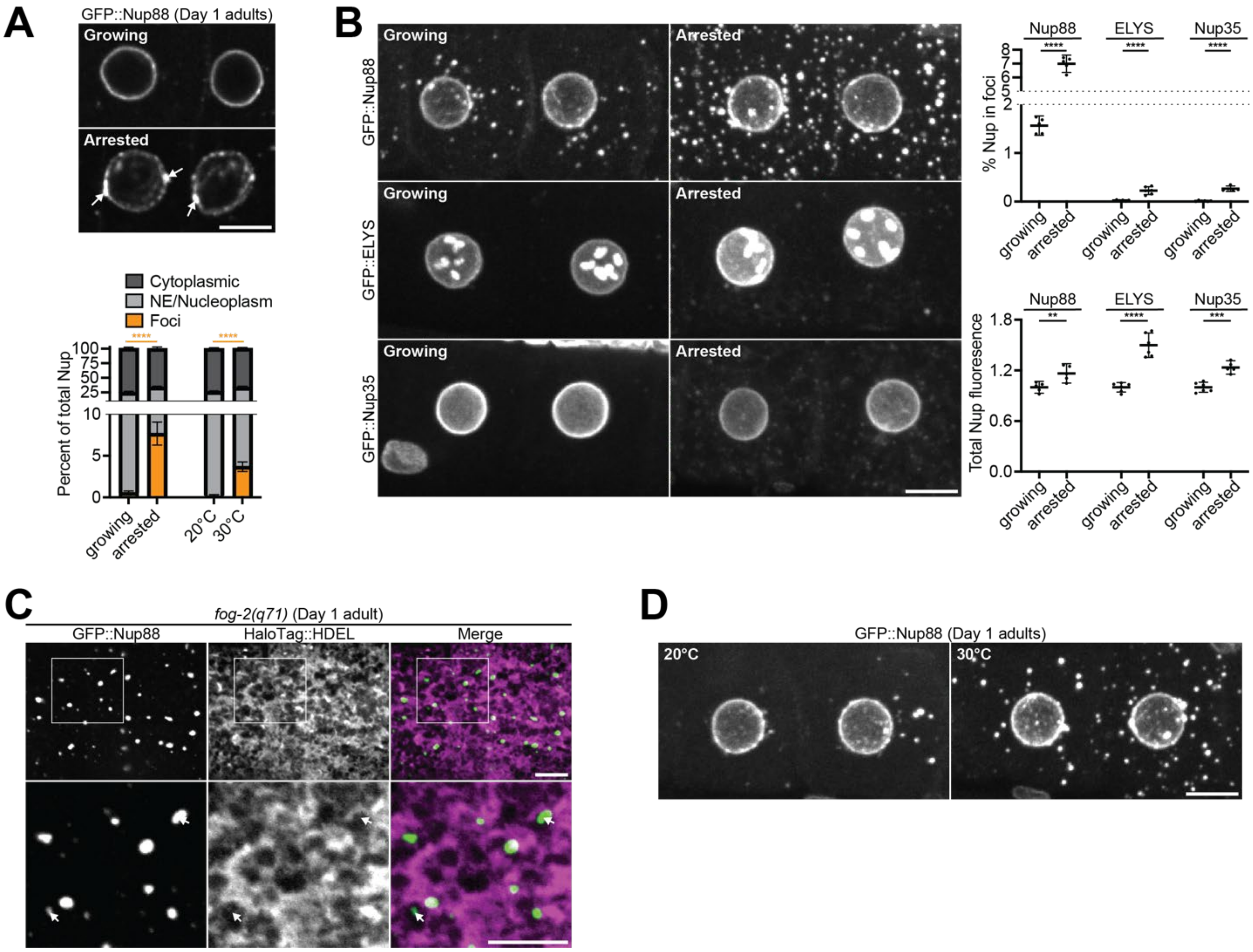

Appendix Figure S4. Nup foci in arrested oocytes enrich additional Nups and overlap more frequently with the endoplasmic reticulum. A. Top: Representative confocal micrographs showing CRISPR-tagged GFP::Nup88 in the -3 and -4 oocytes of Day 1 adults with growing (wild-type) versus arrested (*fog-2(q71)*) oocytes. White arrows denote Nup blebs at the nuclear envelope. Bottom: Compiled quantification of the distribution of Nup between the cytoplasm (soluble), nuclear envelope (NE)/nucleoplasm, and cytoplasmic foci in each indicated condition. Data correspond to micrographs in Figure 4A. Error bars represent 95% CI for n > 7 germlines (growing versus arrested; biological replicates) or n = 6 germlines (20°C versus 30°C; biological replicates). B. Left: Representative confocal micrographs showing the -3 and -4 oocytes of Day 2 adults (growing oocytes) versus Day 4 adults (arrested oocytes) with CRISPR-tagged GFP::Nup88, GFP::ELYS, or GFP::Nup35. Top graph: Quantification of the total percent of each designated Nup in foci in growing versus arrested oocytes. Error bars

represent 95% CI for n > 5 germlines (biological replicates). Bottom graph: Quantification of the total fluorescence of each designated Nup in growing versus arrested oocytes. Values are normalized so that the average fluorescence of growing oocytes = 1.0. Error bars represent 95% CI for n > 5 germlines (biological replicates). C. Representative confocal micrographs showing overlap of GFP::Nup88 with the luminal endoplasmic reticulum/nuclear envelope marker HaloTag::HDEL in a Day 1 adult *fog-2(q71)* arrested oocyte. 42% of foci completely overlapped with HaloTag::HDEL, 55% partially overlapped, and 3% showed no overlap with HaloTag::HDEL (n = 198, see materials and methods). Areas indicated by white boxes are magnified below; white arrows indicate foci that do not completely overlap with the endoplasmic reticulum. D. Representative images showing GFP::Nup88 in the -3 and -4 oocytes of Day 1 adults grown at 20°C or after shifting to 30°C for 20 min.

Data information: ****P<0.0001; ***P<0.001; **P<0.01. Significance was determined using an unpaired *t*-test. All images in this figure are maximum intensity projections, with the exception of panels A and C which are single focal planes. Scale bars = 10 µm.

Appendix Figure S5

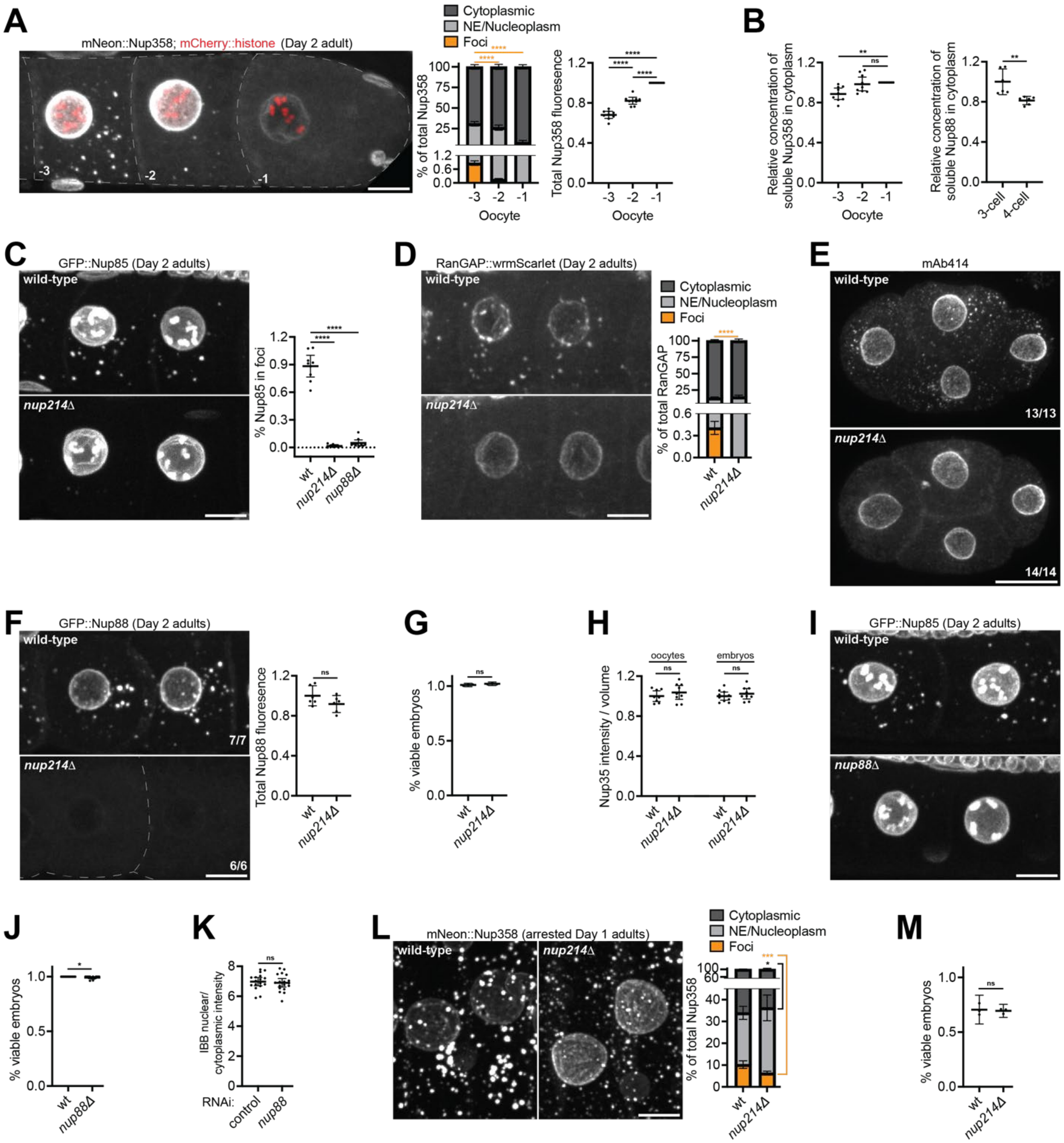

Appendix Figure S5. Robust Nup foci are not required for embryonic viability. A. Left: Representative confocal micrograph showing CRISPR-tagged mNeonGreen::Nup358 in Day 2 adult oocytes. Foci begin to disassemble in the -2 position oocyte, and are absent from the -1 oocyte coincident with nuclear envelope breakdown. Middle: Quantification of the distribution of mNeonGreen::Nup358 between the cytoplasm (soluble), nuclear envelope (NE)/nucleoplasm, and cytoplasmic foci.

Error bars represent 95% CI for n = 8 germlines (biological replicates). Right: Quantification of total mNeonGreen::Nup358 fluorescence in -3, -2, and -1 oocytes. Values are normalized within the same germline so that the value of the -1 oocyte = 1.0. Error bars represent 95% CI for n = 9 germlines (biological replicates). B. Left: Cytoplasmic (soluble) mNeonGreen::Nup358 fluorescence normalized to volume in -3, -2, and -1 oocytes from Day 2 adults. Values are normalized within the same germline so that the -1 oocyte measurement = 1.0. Error bars represent 95% CI for n = 9 germlines (biological replicates). Right: Cytoplasmic (soluble) GFP::Nup88 fluorescence normalized to volume in 3-cell mitotic embryos versus 4-cell interphase embryos. Values are normalized so that the average 3-cell embryo measurement = 1.0. Error bars represent 95% CI for n > 6 embryos (biological replicates). C. Left: Representative confocal micrographs showing CRISPR-tagged GFP::Nup85 in -3 and -4 oocytes of wild-type versus *nup214*Δ Day 2 adults. Right: Quantification of the percent of GFP::Nup85 in foci in wild-type versus *nup214*Δ and *nup88*Δ mutants. Error bars represent 95% CI for n = 9 germlines (biological replicates). See Appendix Figure S5I for representative images of GFP::Nup85 in the *nup88*Δ mutant. D. Left: Representative confocal micrographs showing CRISPR-tagged RanGAP::wrmScarlet in -3 and -4 oocytes of wild-type versus *nup214*Δ Day 2 adults. Right: Quantification of the distribution of RanGAP::wrmScarlet between the cytoplasm (soluble), the NE/nucleoplasm, and cytoplasmic foci in wild-type versus *nup214*Δ oocytes. Error bars represent 95% CI for n = 4 germlines (biological replicates). E. Representative confocal micrographs showing endogenous Nups, visualized by mAb414, in wild-type versus *nup214*Δ 4-cell embryos. 13/13 and 14/14 embryos imaged displayed the represented phenotypes for wild-type and *nup214*Δ mutants, respectively. F. Left: Representative confocal micrographs showing CRISPR-tagged GFP::Nup88 in -3 and -4 oocytes of wild-type versus *nup214*Δ Day 2 adults. 7/7 and 6/6 germlines imaged displayed the represented phenotypes for wild-type and *nup214*Δ mutants, respectively. Right: Quantification of total GFP::Nup88 fluorescence in wild-type versus *nup214*Δ oocytes. Values are normalized so that the average wild-type measurement = 1.0. Error bars represent 95% CI for n = 6 germlines (biological replicates). G. Embryonic viability of wild-type *C. elegans* versus the *nup214*Δ mutant. Error bars represent 95% CI for N = 3

independent experiments with n = 907 (wild-type) or n = 892 (*nup214Δ*) embryos. H. Quantification of the intensity of GFP::Nup35 per nuclear volume in the -3 and -4 oocytes of Day 1 adults or 28-cell stage embryos. Values are normalized so that the average wild-type measurement = 1.0. Error bars represent 95% CI for n > 8 germlines and n > 9 embryos (biological replicates). I. Representative confocal micrographs showing GFP::Nup85 in -3 and -4 oocytes of wild-type versus *nup88Δ* Day 2 adults. See Appendix Figure S5C for quantification. J. Embryonic viability of wild-type *C. elegans* versus the *nup88Δ* mutant. Error bars represent 95% CI for N = 2 independent experiments with n = 525 (wild-type) or n = 520 (*nup88Δ*) embryos. K. Quantification of the nuclear/cytoplasmic ratio of the IBB_{domain}::mNeonGreen reporter in 28-cell stage embryos following control or *nup88* RNAi. Values are normalized so that the average control measurement = 1.0. Error bars represent 95% CI for n > 17 embryos (biological replicates). L. Left: Representative confocal micrographs showing CRISPR-tagged mNeonGreen::Nup358 in arrested -3 and -4 oocytes of wild-type versus *nup214Δ* Day 1 *fog-2(q71)* females. Right: Quantification of the distribution of mNeonGreen::Nup358 between the cytoplasm (soluble), the NE/nucleoplasm, and cytoplasmic foci in arrested oocytes of wild-type versus *nup214Δ* mutants. Error bars represent 95% CI for n = 6 germlines (biological replicates). M. Embryonic viability of arrested wild-type versus *nup214Δ* oocytes following mating. Error bars represent 95% CI for N = 3 independent experiments with n = 180 embryos from 24 mothers (wild-type) or n = 155 embryos from 23 mothers (*nup214Δ*) animals.

Data information: ****P<0.0001; ***P<0.001; **P<0.01; *P<0.05; ns, not significant. For panels A, B (left), and C significance was determined using a one-way ANOVA; for all other panels significance was determined using an unpaired *t*-test. All images in this figure are maximum intensity projections. Scale bars = 10 μm.

Appendix Figure S6

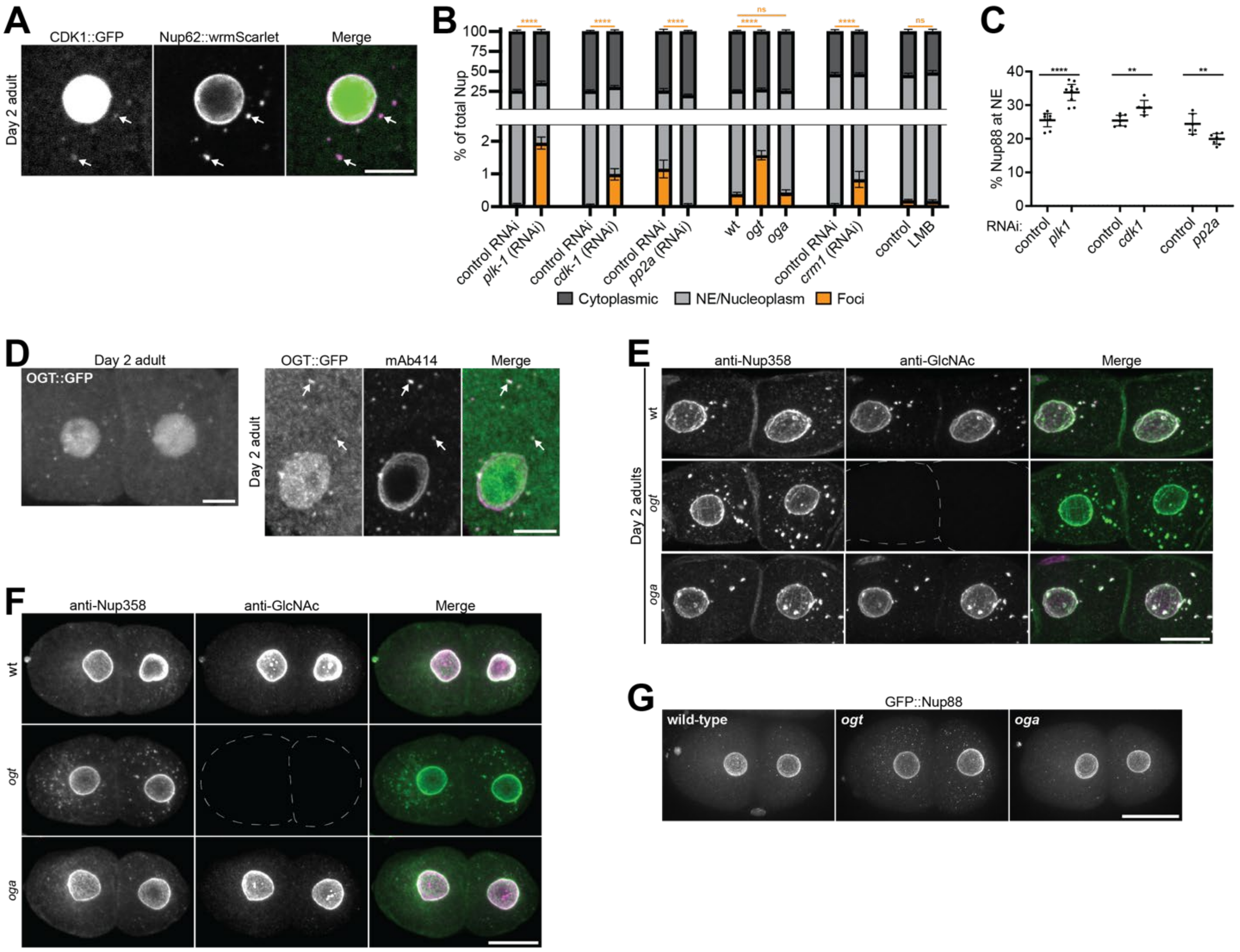

Appendix Figure S6. CDK1 and OGT are enriched at foci and promote Nup solubility. A. Representative confocal micrographs showing localization of CRISPR-tagged Nup62::wrmScarlet relative to CDK1::GFP in a Day 2 adult oocyte. White arrows indicate enrichment of CDK1 in cytoplasmic Nup62 foci. B. Compiled quantification of the distribution of Nup between the cytoplasm (soluble), nuclear envelope (NE)/nucleoplasm, and cytoplasmic foci in each indicated condition. Error bars represent 95% CI (biological replicates) and data correspond to micrographs in Figure 6B (*plk1* RNAi, n > 8 germlines; *cdk1* RNAi, n > 6 germlines; *pp2a* RNAi, n > 6 germlines), Figure 6C (*ogt* and *oga* mutants, n > 6 germlines), and Figure 6E (*crm1* RNAi, n > 7 germlines; LMB treatment, n > 8 germlines). C. Compiled quantification of the percent of GFP::Nup88 at the nuclear envelope (NE) under each indicated condition. Error bars represent 95% CI (biological replicates) and data correspond to

micrographs in Figure 6B (*plk1* RNAi, n > 8 germlines; *cdk1* RNAi, n > 6 germlines; *pp2a* RNAi, n > 6 germlines). D. Left: Representative confocal micrograph showing CRISPR-tagged OGT::GFP in -3 and -4 oocytes of a Day 2 adult. Right: Localization of OGT::GFP versus mAb414 in a Day 2 adult oocyte. White arrows indicate enrichment of OGT in cytoplasmic mAb414 foci. E. Representative confocal micrographs showing localization of endogenous Nup358 versus the RL2 GlcNAc antibody in wild-type, *ogt*, or *oga* Day 2 adult oocytes. F. Representative confocal micrographs showing localization of endogenous Nup358 versus the RL2 GlcNAc antibody in wild-type, *ogt*, or *oga* interphase 2-cell embryos. G. Representative confocal micrographs showing CRISPR-tagged GFP::Nup88 in wild-type, *ogt*, or *oga* 2-cell embryos.

Data information: ****P<0.0001; **P<0.01; ns, not significant. For the *ogt* and *oga* mutants significance was determined using a one-way ANOVA; for all other conditions significance was determined using an unpaired *t*-test. All images in this figure are maximum intensity projections. Scale bars = 10 µm.

Appendix Figure S7

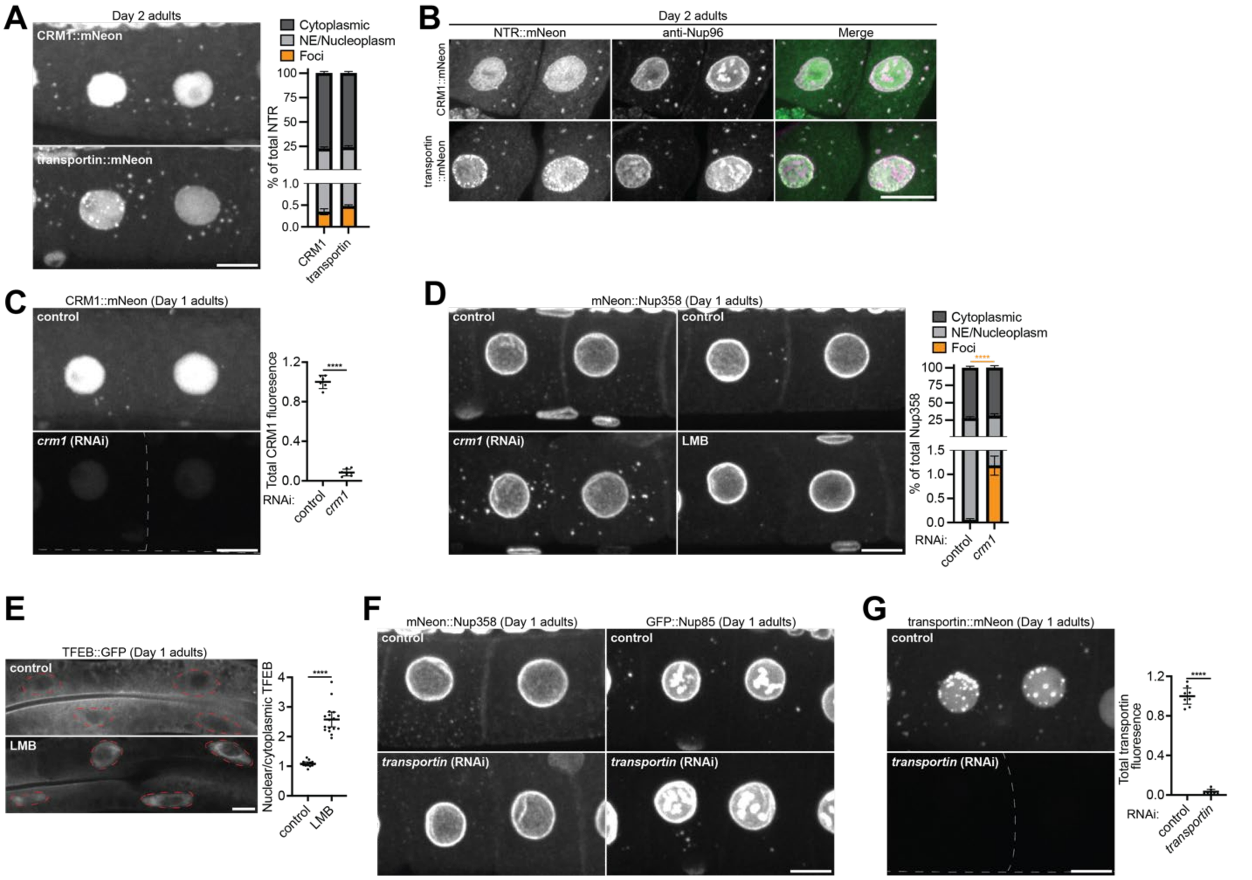

Appendix Figure S7. CRM1 and transportin are enriched in cytoplasmic Nup foci. A. Left: Representative confocal micrographs showing CRISPR-tagged CRM1::mNeonGreen or transportin::mNeonGreen in -3 and -4 oocytes of Day 2 adults. Right: Quantification of the distribution of CRM1::mNeonGreen and transportin::mNeonGreen between the cytoplasm (soluble), nuclear envelope (NE)/nucleoplasm, and cytoplasmic foci. Error bars represent 95% CI for n = 8 germlines (biological replicates). B. Representative confocal micrographs showing localization of CRM1::mNeonGreen and transportin::mNeonGreen relative to endogenous Nup96 in Day 2 adult oocytes. C. Left: Representative confocal micrographs showing CRM1::mNeonGreen in control -3 and -4 oocytes of Day 1 adults or oocytes targeted by *crm1* RNAi. Right: Quantification of total CRM1::mNeonGreen fluorescence in control oocytes or oocytes targeted by *crm1* RNAi. Values are normalized so that the average control measurement = 1.0. Error bars represent 95% CI for n > 6 germlines (biological replicates). D. Left: Representative confocal micrographs

showing CRISPR-tagged mNeonGreen::Nup358 in control -3 and -4 oocytes of Day 1 adults or oocytes depleted of CRM1. Middle: Representative confocal micrographs showing mNeonGreen::Nup358 in control oocytes or following treatment with the CRM1 inhibitor leptomycin B (LMB). Right: Quantification of the distribution of mNeonGreen::Nup358 between the cytoplasm (soluble), NE/nucleoplasm, and cytoplasmic foci in control oocytes or following CRM1 depletion. Error bars represent 95% CI for n > 7 germlines (biological replicates). E. Left: Representative confocal micrographs showing TFEB::GFP in control Day 1 adults or following LMB treatment. Red dashed lines denote nuclei. Right: Quantification of the nuclear/cytoplasmic ratio of TFEB::GFP in control cells or following LMB treatment; note that nuclear export of TFEB is mediated by CRM1 (Silvestrini *et al*, 2018). Error bars represent 95% CI for n > 13 nuclei (biological replicates). F. Representative confocal micrographs showing CRISPR-tagged GFP::Nup85 and mNeonGreen::Nup358 in -3 and -4 oocytes of control Day 1 adults or oocytes depleted of transportin. G. Left: Representative confocal micrographs showing transportin::mNeonGreen in control -3 and -4 oocytes of Day 1 adults or oocytes targeted by *transportin* RNAi. Right: Quantification of total transportin::mNeonGreen fluorescence in control oocytes or oocytes targeted by *transportin* RNAi. Values are normalized so that the average control measurement = 1.0. Error bars represent 95% CI for n > 7 germlines (biological replicates).

Data information: ****P<0.0001. Significance was determined using an unpaired t-test. All images in this figure are maximum intensity projections, with the exception of panel E which are single imaging planes. Scale bars = 10 μm.

Appendix Figure S8

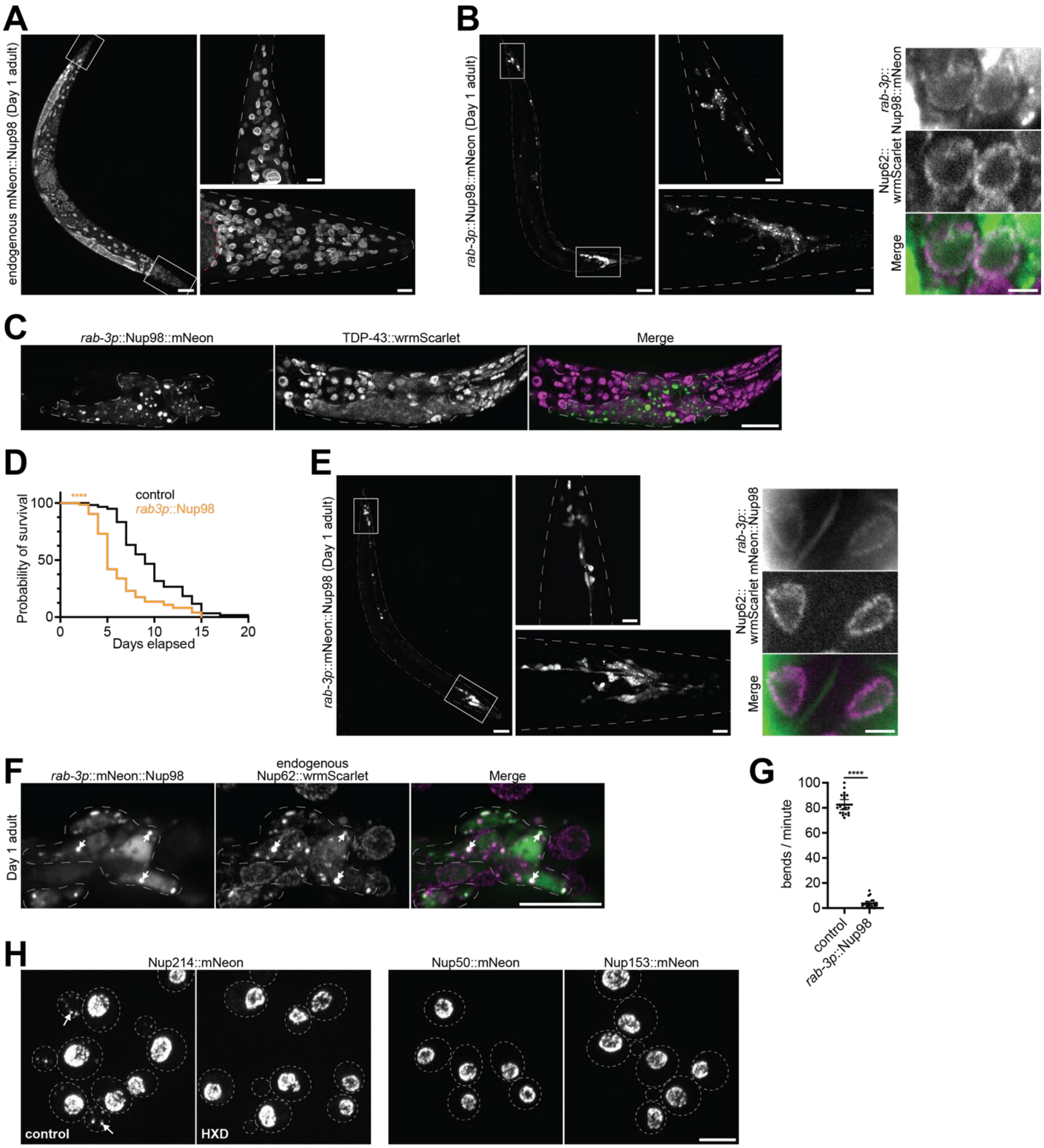

Appendix Figure S8. Ectopic Nup98 condensation in post-mitotic neurons is toxic. A. Representative confocal micrographs showing endogenous CRISPR-tagged mNeonGreen::Nup98 in a Day 1 adult *C. elegans*; images were acquired using 40% laser power. The head and tail indicated by white boxes are magnified in the panels to the right. Red dashes outline part of the germline. B. Left: Representative confocal

micrographs showing transgenic *rab-3p*::Nup98::mNeonGreen in a Day 1 adult *C. elegans*; images were acquired using 3% laser power. The head and tail ganglia indicated by white boxes are magnified in the panels to the right. Right: Representative confocal micrographs showing localization of *rab-3p*::Nup98::mNeonGreen relative to endogenous CRISPR-tagged Nup62::wrmScarlet in neuronal nuclei of a Day 1 adult; images were acquired using 70% laser power. C. Representative confocal micrographs showing localization of CRISPR-tagged TDP-43::wrmScarlet and transgenic *rab-3p*::Nup98::mNeonGreen in the head of an L1 larva. Note that transgenic Nup98::mNeonGreen is only expressed in neurons; dashed line indicates cells with Nup98::mNeonGreen expression. D. Survival curve of control *C. elegans* versus those with ectopically expressed *rab-3p*::Nup98::mNeonGreen. n = 61 (control) or n = 74 (*rab-3p*::Nup98::mNeonGreen) animals (biological replicates). E. Left: Representative confocal micrographs showing transgenic *rab-3p*::mNeonGreen::Nup98 in a Day 1 adult *C. elegans*; images were acquired using 5% laser power. The head and tail ganglia indicated by white boxes are magnified in the panels to the right. Right: Representative confocal micrographs showing localization of *rab-3p*::mNeonGreen::Nup98 relative to endogenous Nup62::wrmScarlet in neuronal nuclei of a Day 1 adult; images were acquired using 70% laser power. F. Representative confocal micrographs showing localization of *rab-3p*::mNeonGreen::Nup98 relative to endogenous Nup62::wrmScarlet in the head of a Day 1 adult. Neurons expressing *rab-3p*::mNeonGreen::Nup98 are designated by grey dashed outlines; white arrows indicate enrichment of endogenous Nup62 at ectopic Nup98 foci. G. Quantification of the swimming behavior of control Day 1 adults versus those with ectopically expressed *rab-3p*::mNeonGreen::Nup98. Error bars represent 95% CI for n > 19 worms (biological replicates). H. Representative confocal micrographs showing endogenous Nup214, Nup50, and Nup153 tagged with mNeonGreen in yeast cells. Yeast expressing Nup214::mNeonGreen were treated with 5% 1,6-hexandiol (HXD) for 10 min prior to imaging. White arrows denote cytoplasmic Nup foci.

Data information: ****P<0.0001. Significance was determined using an unpaired *t*-test. All images in this figure are maximum intensity projections, with the exception of

magnified nuclei in panels B and E which are single focal planes. Scale bars = 50 µm (panels A, B, and E, whole worms), 2 µm (panels B and E, nuclei), 5 µm (panel H), or 10 µm (all other panels).

Name	Description	Genotype	Additional information	Source
BN189	mCherry::histone	bqSi189 [lmn-1p::mCherry::his-58::pie-1utr] II		(Gómez-Saldivar *et al*, 2016)
BN740	GFP::Nup88; mCherry::histone	npp-24(bq15[G>F>P::npp-24]) bqSi189 [lmn-1p::mCherry::his-58::pie-1utr] II		This study
BN1018	GFP::Nup35; mCherry::histone	npp-19(bq29[G>F>P::npp-19]) bqSi189 [lmn-1p::mCherry::his-58::pie-1utr] II		This study
BN1062	TPR::GFP; mCherry::histone	npp-21(bq1[npp-21::GFP]) bqSi189 [lmn-1p::mCherry::his-58::pie-1utr] II		This study
BN452	GFP::ELYS; mCherry::histone	bqSi189 [lmn-1p::mCherry::his-58::pie-1utr] II; mel-28(bq5[GFP::mel-28]) III		Gómez-Saldivar *et al*, 2016
BN69	GFP::Nup107; mCherry::histone	npp-5(tm3039)/mIn1 [mIs14 dpy-10(e128)] II; bqIs51 [pie-1p::GFP::npp-5] ltIs37 [pie-1p::mCherry::his-58] IV		Ródenas *et al*, 2012
JH3850	GFP::Nup85; mCherry::histone	npp-2(bq38[G>F>P::npp-2]) I; bqSi189 [lmn-1p::mCherry::his-58::pie-1utr] II		This study
JH3867	mNeonGreen::Nup98; mCherry::histone	bqSi189 [lmn-1p::mCherry::his-58::pie-1utr] II; npp-10(ax4538[mNeonGreen::npp-10]) III		This study
JH3938	mNeonGreen::Nup358; mCherry::histone	bqSi189 [lmn-1p::mCherry::his-58::pie-1utr] II; npp-9(ax4540[mNeonGreen::npp-9]) III		This study
JH3908	gp210::mNeonGreen; mCherry::histone	npp-12(ax4539[npp-12::mNeonGreen]) I; bqSi189 [lmn-1p::mCherry::his-58::pie-1utr] II		This study
JH4201	Nup62::wrmScarlet	npp-11(ax4547[npp-11::wrmScarlet]) I		This study
JH3906	RanGAP::wrmScarlet	ran-2(ax4545[ran-2::wrmScarlet]) III		This study
JH4202	NDC1::wrmScarlet	npp-22(ax4549[npp-22::wrmScarlet]) V		This study
DG4557	GFP::NDC1	npp-22(tn1794[GFP::3xFLAG::npp-22]) V		Huelgas-Morales *et al*, 2020
OCF22	mCherry::Nup54	ocfIs5 [pie-1p::mCherry::npp-1::pie-1utr]		Joseph-Strauss *et al*, 2012
JH3872	Nup214::OLLAS	npp-14(ax4548[npp-14::OLLAS]) I		This study
JCP519	NXF1::eGFP	nxf-1(t2160) V; jcpEx6 [nxf-1p::nxf-1::3xFLAG::eGFP::nxf-1utr]		Zheleva *et al*, 2019
JH4115	GFP::Nup88; Nup62::wrmScarlet	npp-11(ax4547[npp-11::wrmScarlet]) I; npp-24(bq15[G>F>P::npp-24]) II	cross between BN740 and JH4201	This study
JH4128	GFP::ELYS; Nup62::wrmScarlet	npp-11(ax4547[npp-11::wrmScarlet]) I; mel-28(bq5[GFP::mel-28]) III	cross between BN452 and JH4201	This study
JH4083	GFP::Nup85; Nup62::wrmScarlet	npp-2(bq38[G>F>P::npp-2]) npp-11(ax4547[npp-11::wrmScarlet]) I	cross between JH3850 and JH4201	This study
JH3995	mNeonGreen::Nup98; Nup62::wrmScarlet	npp-11(ax4547[npp-11::wrmScarlet]) I; npp-10(ax4538[mNeonGreen::npp-10]) III	cross between JH3867 and JH4201	This study
JH3986	mNeonGreen::Nup358; Nup62::wrmScarlet	npp-11(ax4547[npp-11::wrmScarlet]) I; npp-9(ax4540[mNeonGreen::npp-9]) III	cross between JH3938 and JH4201	This study
JH4203	gp210::mNeonGreen; Nup62::wrmScarlet	npp-12(ax4539[npp-12::mNeonGreen]) npp-11(ax4547[npp-11::wrmScarlet]) I	cross between JH3908 and JH4201	This study

JH3849	GFP::Nup88; mCherry::histone; HaloTag::HDEL	egxSi126 [mex-5p::hsp-3(1-19)::HaloTag::HDEL::pie-1utr] I; npp-24(bq15[G>F>P::npp-24]) bqSi189 [lmn-1p::mCherry::his-58::pie-1utr] II	HaloTag::HDEL from Fan *et al*, 2020	This study
JK574	fog-2(q71)	fog-2(q71) V		J. Kimble Lab (via the CGC)
JH4384	nup214Δ; fog-2(q71)	npp-14(ax4543) I; fog-2(q71) V	cross between JH3886 and JK574	This study
JH4151	GFP::Nup88; mCherry::histone; HaloTag::HDEL; fog-2(q71)	egxSi126 [mex-5p::hsp-3(1-19)::HaloTag::HDEL::pie-1utr] I; npp-24(bq15[G>F>P::npp-24]) bqSi189 [lmn-1p::mCherry::his-58::pie-1utr] II; fog-2(q71) V	cross between JH3849 and JK574	This study
JH4143	GFP::Nup88; mCherry::histone; fog-2(q71)	npp-24(bq15[G>F>P::npp-24]) bqSi189 [lmn-1p::mCherry::his-58::pie-1utr] II; fog-2(q71) V	cross between BN740 and JK574	This study
JH4392	mNeonGreen::Nup358; mCherry::histone; fog-2(q71)	bqSi189 [lmn-1p::mCherry::his-58::pie-1utr] II; npp-9(ax4540[mNeonGreen::npp-9]) III; fog-2(q71) V	cross between JH3938 and JK574	This study
JH4393	mNeonGreen::Nup358; mCherry::histone; nup214Δ; fog-2(q71)	npp-14(ax4543) I; bqSi189 [lmn-1p::mCherry::his-58::pie-1utr] II; npp-9(ax4540[mNeonGreen::npp-9]) III; fog-2(q71) V	cross between JH3937 and JK574	This study
JH4133	mNeonGreen::Nup358; G3BP::mCherry	npp-9(ax4540[mNeonGreen::npp-9]) III; gtbp-1(ax4560[gtbp-1::mCherry]) IV	cross between JH3323 and JH3938	This study
JH3958	mNeonGreen::Nup98; PGL-3::mCherry	npp-10(ax4538[mNeonGreen::npp-10]) III; pgl-3(ax4300[pgl-3::mCherry]) V		This study
JH3656	G3BP::RFP; fog-2(q71)	gtbp-1(ax5000[gtbp-1::tagRFP]) IV; fog-2(q71) V; meg-3(ax3054[meg-3::GFP]) X		This study
N2	C. elegans wild isolate			
RB653	OGT mutant	ogt-1(ok430) III		The C. elegans Deletion Mutant Consortium, 2012
RB1169	OGA mutant	oga-1(1207) X		The C. elegans Deletion Mutant Consortium, 2012
JH3859	GFP::Nup88; mCherry::histone; OGT mutant	npp-24(bq15[G>F>P::npp-24]) bqSi189 [lmn-1p::mCherry::his-58::pie-1utr] II; ogt-1(ok430) III	cross between BN740 and RB653	This study
JH3869	GFP::Nup88; mCherry::histone; OGA mutant	npp-24(bq15[G>F>P::npp-24]) bqSi189 [lmn-1p::mCherry::his-58::pie-1utr] II; oga-1(1207) X	cross between BN740 and RB1169	This study
OG1124	OGT::GFP	ogt-1(dr84[ogt-1::GFP]) III		Urso *et al*, 2020
JH4076	Nup62::wrmScarlet; CDK1::GFP	npp-11(ax4547[npp-11::wrmScarlet]) I; neSi12 [cdk-1::GFP] II	CDK1::GFP from Shirayama *et al*, 2012	This study
JH3955	CRM1::mNeonGreen; mCherry::histone	bqSi189 [lmn-1p::mCherry::his-58::pie-1utr] II; xpo-1(ax4542[xpo-1::mNeonGreen]) V		This study

IX4676	transportin::mNeonGreen	imb-2(vy280[imb-2::mNeonGreen::3xFLAG]) II		Alqadah *et al*, 2019
MAH240	TFEB::GFP	sqIs17 [hlh-30p::hlh-30::GFP]		Lapierre *et al*, 2013
JH3886	nup214Δ	npp-14(ax4543) I		This study
JH3937	mNeonGreen::Nup358; mCherry::histone; nup214Δ	npp-14(ax4543) I; bqSi189 [lmn-1p::mCherry::his-58::pie-1utr] II; npp-9(ax4540[mNeonGreen::npp-9]) III	cross between JH3938 and JH3886	This study
JH3912	GFP::Nup88; mCherry::histone; nup214Δ	npp-14(ax4543) I; npp-24(bq15[G>F>P::npp-24]) bqSi189 [lmn-1p::mCherry::his-58::pie-1utr] II	cross between BN740 and JH3886	This study
JH4427	RanGAP::wrmScarlet; nup214Δ	ran-2(ax4545[ran-2::wrmScarlet]) III; npp-14(ax4543) I	cross between JH3906 and JH3886	This study
JH3895	GFP::Nup35; mCherry::histone; nup214Δ	npp-14(ax4543) I; npp-19(bq29[G>F>P::npp-19]) bqSi189 [lmn-1p::mCherry::his-58::pie-1utr] II	cross between BN1018 and JH3886	This study
JH4375	GFP::Nup85; mCherry::histone; nup214Δ	npp-2(bq38[G>F>P::npp-2]) npp-14(ax4543) I; bqSi189 [lmn-1p::mCherry::his-58::pie-1utr] II	cross between JH3850 and JH3886	This study
JH4012	IBB_{domain}::mNeonGreen	gtbp-1(ax4561[gtbp-1p::IBB_{domain}::mNeonGreen::gtbp-1utr]) IV		This study
JH3936	IBB_{domain}::mNeonGreen; nup214Δ	npp-14(ax4543) I; gtbp-1(ax4561[gtbp-1p::IBB_{domain}::mNeonGreen::gtbp-1utr]) IV	cross between JH4012 and JH3886	This study
JH3904	TDP-43::wrmScarlet	tdp-1(ax4546[tdp-1::wrmScarlet]) II		This study
JH4235	TDP-43::wrmScarlet; nup214Δ	npp-14(ax4543) I; tdp-1(ax4546[tdp-1::wrmScarlet]) II	cross between JH3904 and JH3886	This study
JH3985	CRM1::mNeonGreen; mCherry::histone; nup214Δ	npp-14(ax4543) I; bqSi189 [lmn-1p::mCherry::his-58::pie-1utr] II; xpo-1(ax4542[xpo-1::mNeonGreen]) V	cross between JH3955 and JH3886	This study
JH3323	G3BP::mCherry	gtbp-1(ax4560[gtbp-1::mCherry]) IV		Paix *et al*, 2015
JH3902	G3BP::mCherry; nup214Δ	npp-14(ax4543) I; gtbp-1(ax4560[gtbp-1::mCherry]) IV	cross between JH3323 and JH3886	This study
BN901	nup88Δ; mCherry::histone	npp-24(bq22[G>P::npp-24]) bqSi189 [lmn-1p::mCherry::his-58::pie-1utr] II	Cross between BN740 and BN711 (Macías-León & Askjaer, 2018)	This study
BN1436	GFP::Nup85; mCherry::histone; nup88Δ	npp-2(bq38[G>F>P::npp-2]) I; npp-24(bq22[G>P::npp-24]) bqSi189 [lmn-1p::mCherry::his-58::pie-1utr] II	cross between BN901 and BN1044 (npp-2(bq38[G>F>P::npp-2]))	This study
JH3959	mNeonGreen::Nup358; gp210Δ	npp-12(ax4544) I; npp-9(ax4540[mNeonGreen::npp-9]) III		This study
JH4119	mex-5p::Nup214::wrmScarlet; mNeonGreen::Nup358	ax4541 [mex-5p::npp-14::wrmScarlet::ollas::tbb-2utr] I; npp-9(ax4540[mNeonGreen::npp-9]) III		This study
JH4204	rab-3p::Nup98::mNeonGreen; Nup62::wrmScarlet	npp-11(ax4547[npp-11::wrmScarlet]) I; ax4550 [rab-3p::npp-10(1-919)::mNeonGreen::tbb-2utr]		This study
JH4395	rab-3p::mNeonGreen::Nup98; Nup62::wrmScarlet	npp-11(ax4547[npp-11::wrmScarlet]) I; ax4574 [rab-3p::mNeonGreen::npp-10(1-919)::tbb-2utr]		This study

JH4205	rab-3p::Nup98::mNeonGreen; TDP-43::wrmScarlet	tdp-1::wrmScarlet (ax4546) II; ax4550 [rab-3p::npp-10(1-919)::mNeonGreen::tbb-2utr]		This study
SEY6210.1		*MATa ura3-52 his3-Δ200 leu2-3,112 lys2-801 trp1-Δ901 suc2-Δ9*		Robinson *et al*, 1988
LTY3	Nup214::mNeonGreen	*MATa ura3-52 his3-Δ200 leu2-3,112 lys2-801 trp1-Δ901 suc2-Δ9 nup159::mNeonGreen::HIS3*	Integration into SEY6210.1	This study
LTY6	Nup153::mNeonGreen	*MATa ura3-52 his3-Δ200 leu2-3,112 lys2-801 trp1-Δ901 suc2-Δ9 nup60::mNeonGreen::HIS3*	Integration into SEY6210.1	This study
LTY8	Nup50::mNeonGreen	*MATa ura3-52 his3-Δ200 leu2-3,112 lys2-801 trp1-Δ901 suc2-Δ9 nup2::mNeonGreen::HIS3*	Integration into SEY6210.1	This study

Appendix Table S1. *C. elegans* and yeast strains generated and used in this study.

Appendix References

Alqadah A, Hsieh Y-W, Xiong R, Lesch BJ, Chang C, Chuang C-F (2019) A universal transportin protein drives stochastic choice of olfactory neurons via specific nuclear import of a sox-2-activating factor. *Proc Natl Acad Sci U S A* 116: 25137 – 25146

Fan X, De Henau S, Feinstein J, Miller SI, Han B, Frøkjær-Jensen C, Griffin EE (2020) SapTrap assembly of *Caenorhabditis elegans* MosSCI transgene vectors. *G3 (Bethesda)* 10: 635 – 644

Gómez-Saldivar G, Fernandez A, Hirano Y, Mauro M, Lai A, Ayuso C, Haraguchi T, Hiraoka Y, Piano F, Askjaer P (2016) Identification of conserved MEL-28/ELYS domains with essential roles in nuclear assembly and chromosome segregation. *PLoS Genet* 12: e1006131

Huelgas-Morales G, Sanders M, Mekonnen G, Tsukamoto T, Greenstein D (2020) Decreased mechanotransduction prevents nuclear collapse in a *Caenorhabditis elegans* laminopathy. *Proc Natl Acad Sci* 117: 31301 – 31308

Joseph-Strauss D, Gorjánácz M, Santarella-Mellwig R, Voronina E, Audhya A, Cohen-Fix O (2012) Sm protein down-regulation leads to defects in nuclear pore complex disassembly and distribution in *C. elegans* embryos. *Dev Biol* 365: 445 – 457

Lapierre LR, Magalhaes Filho CD, McQuary PR, Chu C, Visvikis O, Chang JT, Gelino S, Ong B, Davis AE, Irazoqui JE *et al* (2013) The TFEB orthologue HLH-30 regulates autophagy and modulates longevity in *Caenorhabditis elegans*. *Nat Commun* 4: 2267

Paix A, Folkmann A, Rasoloson D, Seydoux G (2015) High efficiency, homology-directed genome editing in *Caenorhabditis elegans* using CRISPR-Cas9 ribonucleoprotein complexes. *Genetics* 201: 47 – 54

Robinson JS, Klionsky DJ, Banta LM, Emr SD (1988) Protein sorting in *Saccharomyces cerevisiae*: isolation of mutants defective in the delivery and processing of multiple vacuolar hydrolases. *Mol Cell Biol* 8: 4936 – 4948

Ródenas E, González-Aguilera C, Ayuso C, Askjaer P (2012) Dissection of the NUP107 nuclear pore subcomplex reveals a novel interaction with spindle assembly checkpoint protein MAD1 in *Caenorhabditis elegans*. *Mol Biol Cell* 23: 930 – 944

Shirayama M, Seth M, Lee H-C, Gu W, Ishidate T, Conte D, Mello CC (2012) piRNAs initiate an epigenetic memory of nonself RNA in the *C. elegans* germline. *Cell* 150: 65 – 77

Silvestrini MJ, Johnson JR, Kumar AV, Thakurta TG, Blais K, Neill ZA, Marion SW, St. Amand V, Reenan RA, Lapierre LR (2018) Nuclear export inhibition enhances HLH-30/TFEB activity, autophagy, and lifespan. *Cell Rep* 23: 1915 – 1921

The *C. elegans* Deletion Mutant Consortium (2012) Large-scale screening for targeted knockouts in the *Caenorhabditis elegans* genome. *G3 (Bethesda)* 2: 1415 – 1425

Urso SJ, Comly M, Hanover JA, Lamitina T (2020) The O-GlcNAc transferase OGT is a conserved and essential regulator of the cellular and organismal response to hypertonic stress. *PLoS Genet* 16: e1008821

Zheleva A, Gómez-Orte E, Sáenz-Narciso B, Ezcurra B, Kassahun H, de Toro M, Miranda-Vizuete A, Schnabel R, Nilsen H, Cabello J (2019) Reduction of mRNA export unmasks different tissue sensitivities to low mRNA levels during *Caenorhabditis elegans* development. *PLoS Genet* 15: e1008338
