# Supplementary material for: Nucleoporin foci are stress‐sensitive condensates dispensable for C. elegans nuclear pore assembly
Source: EMBO J. 2023 May 31;42(13):e112987. doi: 10.15252/embj.2022112987 (PMC10308366; doi:10.15252/embj.2022112987)
Supplement: Supplementary file 3 — Movie EV2 [file EMBJ-42-e112987-s015.zip › Movie EV2 legend.docx]

**Movie EV2. Nup foci, marked by mNeonGreen::Nup98, fully disassemble during mitosis.** Representative time-lapse images of CRISPR-tagged mNeonGreen::Nup98 (grey) with a mCherry::histone transgene (red) in a *C. elegans* embryo. Images are 15 μm maximum intensity projections at 2 min intervals.
