# Supplementary material for: Nucleoporin foci are stress‐sensitive condensates dispensable for C. elegans nuclear pore assembly
Source: EMBO J. 2023 May 31;42(13):e112987. doi: 10.15252/embj.2022112987 (PMC10308366; doi:10.15252/embj.2022112987)
Supplement: Supplementary file 4 — Movie EV3 [file EMBJ-42-e112987-s004.zip › Movie EV3 legend.docx]

**Movie EV3.** **Swimming assay with control *C. elegans*.** Day 1 adults were placed in M9 media at room temperature and immediately imaged. This movie is related to Figure 7C as well as Movie EV4 (Day 1 adults expressing *rab-3p*::Nup98::mNeonGreen). Movie speed is real time.
