# Supplementary material for: Nucleoporin foci are stress‐sensitive condensates dispensable for C. elegans nuclear pore assembly
Source: EMBO J. 2023 May 31;42(13):e112987. doi: 10.15252/embj.2022112987 (PMC10308366; doi:10.15252/embj.2022112987)
Supplement: Supplementary file 5 — Movie EV4 [file EMBJ-42-e112987-s016.zip › Movie EV4 legend.docx]

**Movie EV4. Swimming assay with *C. elegans* expressing *rab-3p*::Nup98::mNeonGreen.** Day 1 adults were placed in M9 media at room temperature and immediately imaged. This movie is related to Figure 7C as well as Movie EV3 (control Day 1 adults lacking *rab-3p*::Nup98::mNeonGreen). Movie speed is real time.
