# Supplementary material for: Nucleoporin foci are stress‐sensitive condensates dispensable for C. elegans nuclear pore assembly
Source: EMBO J. 2023 May 31;42(13):e112987. doi: 10.15252/embj.2022112987 (PMC10308366; doi:10.15252/embj.2022112987)
Supplement: Supplementary file 6 — Movie EV5 [file EMBJ-42-e112987-s008.zip › Movie EV5 legend.docx]

**Movie EV5.** **Swimming assay with control *C. elegans*.** Day 1 adults were placed in M9 media at room temperature and immediately imaged. This movie is related to Appendix Figure S8G as well as Movie EV6 (Day 1 adults expressing *rab-3p*::mNeonGreen::Nup98). Movie speed is real time.
