# Supplementary material for: Nucleoporin foci are stress‐sensitive condensates dispensable for C. elegans nuclear pore assembly
Source: EMBO J. 2023 May 31;42(13):e112987. doi: 10.15252/embj.2022112987 (PMC10308366; doi:10.15252/embj.2022112987)
Supplement: Supplementary file 16 [file EMBJ-42-e112987-s003.pdf]

THE EMBO JOURNAL

Nucleoporin foci are stress-sensitive condensates dispensable for C. elegans nuclear pore assembly

Laura Thomas, Basma Taleb Ismail, Peter Askjaer, and Geraldine Seydoux
DOI: 10.15252/embj.2022112987

Corresponding author(s): Geraldine Seydoux (gseydoux@jhmi.edu)

Review Timeline:

Submission Date:	2nd Nov 22
Editorial Decision:	8th Dec 22
Revision Received:	10th Mar 23
Editorial Decision:	20th Apr 23
Revision Received:	2nd May 23
Accepted:	10th May 23

Editor: Ieva Gailite

Transaction Report:

(Note: With the exception of the correction of typographical or spelling errors that could be a source of ambiguity, letters and reports are not edited. Depending on transfer agreements, referee reports obtained elsewhere may or may not be included in this compilation. Referee reports are anonymous unless the Referee chooses to sign their reports.)

Dear Dr Seydoux,

Thank you for submitting your manuscript for consideration by the EMBO Journal. I have now taken over the handling of your submission from my colleague Stefanie Böhm. We have now received comments from three reviewers, which are included below for your information.

As you will see from the reports, the reviewers appreciate the work, while also indicating a number of aspects that would need to be strengthened in the revised manuscript before they can recommend acceptance here. In particular, they find that a broader set of Nups need to be included in the analysis, the data regarding the functional relevance of Nup foci need further testing in stressed or arrested oocytes, and they request inclusion of electron microscopy analysis for quantification of the annulate lamellae formation. If electron microscopy analysis is not possible, the reviewers have indicated in the cross-commenting session that this aspect then needs toning down or further description of the approach taken for the quantification and an explanation why only a full overlap of the markers was used for quantification needs to be added. Finally, they indicate that the manuscript needs better integration within the existing literature and some conclusions need toning down.

From my side, I find the reviewer comments generally reasonable. Therefore, based on these broadly positive assessments, I would like to invite you to address the issues raised by the reviewers in a revised manuscript. I should add that it is The EMBO Journal policy to allow only a single major round of revision and that it is therefore important to resolve the main concerns at this stage. I think it would be useful to discuss the revision in more detail via email or phone/videoconferencing - please let me know which option you prefer.

We generally allow three months as standard revision time, which can be extended to six months in case of more extensive revisions. As a matter of policy, competing manuscripts published during this period will not negatively impact on our assessment of the conceptual advance presented by your study. However, please contact me as soon as possible upon publication of any related work to discuss the appropriate course of action. Should you foresee a problem in meeting this three-month deadline, please contact us to arrange an extension.

When preparing your letter of response to the referees' comments, please bear in mind that this will form part of the Review Process File and will therefore be available online to the community. For more details on our Transparent Editorial Process, please visit our website: https://www.embopress.org/page/journal/14602075/authorguide#transparentprocess. Please also see the attached instructions for further guidelines on preparation of the revised manuscript.

Please feel free to contact me if you have any further questions regarding the revision. Thank you for the opportunity to consider your work for publication, and I look forward to your revision.

With best regards,

Ieva

Ieva Gailite, PhD
Senior Scientific Editor
The EMBO Journal
Meyerhofstrasse 1
D-69117 Heidelberg
Tel: +4962218891309
i.gailite@embojournal.org

Instructions for preparing your revised manuscript:

Please make sure you upload a letter of response to the referees' comments together with the revised manuscript.

Please also check that the title and abstract of the manuscript are brief, yet explicit, even to non-specialists.

When assembling figures, please refer to our figure preparation guideline in order to ensure proper formatting and readability in print as well as on screen:
https://bit.ly/EMBOPressFigurePreparationGuideline
See also guidelines for figure legends: https://www.embopress.org/page/journal/14602075/authorguide#figureformat

At EMBO Press we ask authors to provide source data for the main manuscript figures. Our source data coordinator will contact

you to discuss which figure panels we would need source data for and will also provide you with helpful tips on how to upload and organize the files.

IMPORTANT: When you send the revision we will require
- a point-by-point response to the referees' comments, with a detailed description of the changes made (as a word file).
- a word file of the manuscript text.
- individual production quality figure files (one file per figure)
- a complete author checklist, which you can download from our author guidelines (https://www.embopress.org/page/journal/14602075/authorguide).
- Expanded View files (replacing Supplementary Information)
Please see out instructions to authors
https://www.embopress.org/page/journal/14602075/authorguide#expandedview

Please remember: Digital image enhancement is acceptable practice, as long as it accurately represents the original data and conforms to community standards. If a figure has been subjected to significant electronic manipulation, this must be noted in the figure legend or in the 'Materials and Methods' section. The editors reserve the right to request original versions of figures and the original images that were used to assemble the figure.

Further information is available in our Guide For Authors: https://www.embopress.org/page/journal/14602075/authorguide

We realize that it is difficult to revise to a specific deadline. In the interest of protecting the conceptual advance provided by the work, we recommend a revision within 3 months (8th Mar 2023). Please discuss the revision progress ahead of this time with the editor if you require more time to complete the revisions.

Referee #1:

In this paper, the authors observe and systematically study cytoplasmic nucleoporin (Nup) foci in C. elegans.
In young C. elegans, cytoplasmic foci containing Nup358 and Nup88 are observed in gametes and early embryos, but not in somatic cells. Stress conditions such as oocyte arrest, heat stress, or aging also increased Nup foci assembly.
Foci contain some types of Nups (ex, FG-Nups and cytoplasmic filament Nups) and their interactors (Y complex Nups, Nup88, RanGAP, NXF1) but not others (ex, transmembrane or nuclear Nups, Tpr...). The stoichiometry of the Nups in these foci was not fixed. The foci did not always associate with ER membranes. The authors concluded that the foci are unlikely to be annulate lamellae or nuclear pore complex assembly intermediates.
Upon Nup214 depletion, foci number was greatly reduced, but the embryos were still viable and cargoes partitioned correctly, suggesting the formation of nuclear pore complexes. The authors conclude that since NPCs still form in the absence of foci, the foci do not contribute significantly to NPC assembly.
When looking at the distributions of different Nups in the cell, the authors found that the majority show the following distribution: 30-40% nucleoplasm + NE, 60-70% cytoplasm, and around 3% in the foci. RNAi-mediated depletion of any foci-related Nups caused a general decrease in foci formation. On the other hand, depletion of non-foci Nups (such as transmembrane or nuclear) had no effect on the foci. Meanwhile, overexpression of Nup214 increased the amount of Nup358 in the foci. The authors conclude that Nup foci assembly depends on the cumulative effect of high concentrations of the cytoplasmic and inner channel FG-Nups in the cytoplasm, and the foci arise whenever the cytoplasmic concentration of FG-Nups exceeds their saturation concentration.
Nup foci disassembled during mitosis, suggesting possible mechanisms to make Nups more soluble in the cytoplasm. The authors tested different proteins for their effect on reducing foci (thus increasing Nup solubility). Depletion of kinases PLK1 and CDK1, decrease in Nup O-GlcNAcylation, and depletion of transport receptor Crm1 all increased the proportion of Nups in foci (suggesting these increase Nup solubility in the cytoplasm) while depletion of PP2A decreased the proportion of Nups in the foci. The authors concluded that Nups can get solubilized by phosphorylation, O-GlcNAcylation, or transport receptors such as Crm1. Finally, over-expression of Nup98 in neurons resulted in its accumulation in cytoplasmic foci, partial depletion of endogeneous Nup62 from the NPC, transport defects, and neuronal dysfunction. The authors concluded that Nup cytoplasmic foci can be toxic for the cells.
In conclusion, the authors show that these foci are formed in certain cell types and under stress conditions, that they are probably formed due to the propensity of Nups to form foci after exceeding the saturation concentration in the cytoplasm.

I think it is very important that this study raises awareness that cytoplasmic FG foci might not be of physiological relevance in all cases. However, I also do not feel that their negativism about other people's work is sufficiently justified. E.g., while I really like the data and how it is presented in this work, I also find the work from e.g. Hampoelz et al (which they seem to criticize directly

and indirectly) which was obtained in different species and different experimental settings very convincing.
I think the authors put too much emphasis on the non-essentiality of the foci (it is also in the title). Their arguments for this are:
1. The Nup foci seem to form whenever the cumulative concentration of foci Nup in the cytoplasm increases beyond the saturation concentration
2. Multiple mechanisms in the cell serve to 'dissolve' these foci by 'solubilizing' Nups in the cytoplasm
3. The Nup foci are unlikely to function in NPC biogenesis or Annulate Lamellae
Conclusion: The Nup foci have no essential function.
In my opinion, the conclusion does not completely follow. The Nup foci may well have functions outside of what the authors have probed. For example, they could have beneficial functions under specific circumstances. It is always difficult to prove that something has no function whatsoever. I also think that such a conclusion does not necessarily add much to the paper. It is enough to state that the foci form from Nups exceeding a certain concentration collectively and that not taking this into account can lead to misinterpretation in some (BUT NOT ALL) cases.

After addressing this important point and the points below (most of them do not request new experiemnts), I feel the paper would be in good shape to publish.
Major comments
1.
In Figure 7A, S8 etc, the authors use C-terminally tagged Nup98. At which position in Nup98 did the authors insert the fluorescent protein? Could they provide a reference to show its functionality, or data to support this? See for example PMC2150585, Nup98 is produced either as a fusion with Nup96 or with a shorter C-terminal region which is proteolytically cleaved. This could be important for Nup98's localization to the NPC. In Figure 7A, the rab-3p::Nup98-mNeon does not seem to localize to the NE. Is it because of the image brightness settings, or did the authors not observe any localization to the NE at all? Ectopically expressed Nup98 should also localize to the NPC. Usually, N-terminally tagged Nup98 is preferred. Moreover, is the effect of depleting other Nups from the NE/NPC only for Nup98 or is it general? For example, did the authors also observe a partial depletion in Nup62 (or other Nups) from the NE following Nup214 overexpression (mentioned in Figure 4)?

2. I expand here on the topic on Nup foci in C. elegans vs a role in NPC biogenesis or annulate lamellae (AL). It would be good in terms of readability if the authors expand on this by giving specific examples from literature or more detailed reasoning on how AL change from their foci. For example, here are the premises from the Discussion, as to why their foci are probably not AL's:
a. "1) lack Nups essential for pore assembly including transmembrane Nups" - authors could shortly explain that AL in (conditions, organisms, systems, etc) contain also transmembrane Nups
b. "2) account for less than 3% of total Nup molecules," - How does this premise make it unlikely that the foci are AL? How much Nup should the AL contain?
c. "3) display heterogeneous Nup stoichiometry," - what kind of stoichiometry do AL's show?
d. "4) do not colocalize with membranes" - again, some short explanation on findings regarding AL's could be good.
3. On page 8: the authors write: ""In vitro, FG-Nups readily condense into hydrogels (Labokha et al, 2012) raising the possibility that cytoplasmic Nup foci might form by spontaneous condensation of FG-Nups in the saturated environment of the oocyte." I feel strongly that this gives the wrong impression. FG Nups where shown to phase separate after pH shift or from lyophilized powder or by diluting from highly concnetraetd denaturant. None of this I feel fit the description of "readily condense".
4. On page 9: ""FG-Nup hydrogels assembled in vitro are readily dissolved by the aliphatic alcohol 1,6-hexanediol (Schmidt & Görlich, 2015), which disrupts hydrophobic interactions and has been reported to dissolve Nup foci in yeast, Drosophila, and HeLa cells (Hampoelz et al, 2019b; Patel et al, 2007; Agote-Aran et al, 2020)." This statement also gives a wrong impression. The cited paper (Schmidt & Görlich, 2015) studied many Nups, but in fact shows only hexandiol solubilization for two selected Nups from specific species (S. cerevisiae Nup116 and Tetrahymena Mac98A FG). For other hydrogels it is has not been experimentally reported, and in fact, many FG Nup hdyrogels are probably not hexanediol sensitive.
Minor comments
1. Figure 2 - % Nup in foci, how is that calculated? I did not see this.
2. Figure S4C - maybe lower the x-axis so that the data for Nup35 depletion is more visible (it looks like it is zero but not seen)
3. Figure 5A - Caption says "Nup foci accumulate progressively throughout oocyte growth and peak in the - 3 and -4 oocytes" but figure doesn't show that. No need to be in the figure caption
4. Figure 5C has quantification, 5D, S5C and S5D could also benefit from quantification.
5. Most figures which claim co-localization lack quantification. Sometimes it is obvious from the positioning of the puncta/foci, however an analysis could make it better.
6. Figure S7E, it would be good to show the nuclei with an outline, especially in the control image.
7. Similarly in Figure S8A, it is unclear from the figure that those are cytoplasmic foci, the cell and nuclei outline in an inset would make it clearer.
8. Some figures show the bar graph with cytoplasmic, NE/nucleoplasm and foci % of total Nup (ex, Figure 4C). Some figures show Fold change of Nup in foci onl;y (ex, Figure 6A). I assume also Figure 6 is calculated from similar data, i.e. the nuclear, cytoplasmic and foci Nup was calculated. In that case, also showing the cytoplasmic and NE/nucleoplasm Nup amounts for the other figures could be beneficial. Especially in the parts where the authors make claims about Nup solubility, it would be good to confirm that cytoplasmic Nup amounts have increased while foci Nup amounts decreased.
10. Rephrase "Nup foci in oocytes only contain FG-Nups and their direct binding partners". It reads as if no other protein might be present in the foci. "... contain (...)-Nups and their partners, but not (...)-Nups" could read more accurately
11. Rephrase "As expected, we found that hexanediol treatment dissolved Nup foci in C. elegans, although it had no effect on

Nups at the nuclear envelope (Figure S4H)" - as "NE stain remained", because from the images it cannot be seen that there is "no effect"; no quantification is provided.
12. The authors could rewrite the abstract to put more focus on their findings and less on the interpretations. "Our results suggest that Nup foci are non-essential, "accidental", and potentially toxic condensates whose assembly is actively suppressed in healthy cells" - this sentence is highly speculative. Over-expressed Nup98::mNeon was toxic but its functionality in the cell is not clear, (see major comment 1). Over-expression is non-physiological anyway. "Non-essential" is too generalizing, (see major comment 2). Active suppression of foci in healthy cells has also not been shown. The authors show that there are mechanisms which reduce the foci formation, potentially by increasing their solubility in the cytoplasm (although this has been deduced due to the apparent decrease in foci). However, if the authors claim that this is an "active suppression" they should provide more evidence for this.

Referee #2:

This study by Thomas et al. provides a phenotypic description of Nucleoporin (Nup) foci in C. elegans cells. Combining genetic perturbations and light microcopy, the authors observe that Nups form condensates in tissues that express high levels of Nups, including oocytes and embryos. The study furthermore shows that Nup foci consist mainly of FG-Nups and that reduction of certain Nups leads to reduction in Nup foci. Moreover, reduction of Nup214 results in viable embryos, while overexpression of Nup98 results in neuronal toxicity. Overall, this is an interesting study in an organismal context that takes a closer look at NUP condensation in relation to various stress conditions. However, the impact of this study is presently somewhat limited as only a few and variable NUPs are scrutinized in some of the key experiments. Here, a broader experimental footing is necessary to support the author's key conclusions. Specifically, the inclusion of a consistent set of Nups both as readouts for condensates and their more widespread depletion throughout various assays is recommended to allow meaningful comparisons. In addition, the reliance on fluorescence microscopy as singlular readout seems insufficient and additional techniques (especially EM which is the standard in the field) seem essential for clarifying the relationship (or the lack thereof) between the ER membranes, condensates, and NPCs/annulate lamellae (ALs). Lastly, (too) firm conclusions are made connecting NUP solubility and PTMs, but without monitoring NUP modifications directly. Overall, a significant number of additional experiments are required to support the present conclusions and more moderate, less generalizing statements are asked for.

Major points:

1) The authors tend to overinterpret their conclusions on several occasions (on a side note, page/line numbers would have been helpful to point these out). For example, the authors cannot rule out that NUP foci are intermediates of assembly in other organisms or in a distinct physiological context in the same organism. This limitation should be specifically stated. Note also that Hampoelz et al. showed recently in Drosophila that certain NPC components incorporate only later when the condensates transition into full NPCs. Therefore, a more time-resolved analysis monitoring several representative NUPs would be useful. Lastly, NUP accumulation in foci as part of a normal physiological function/assembly intermediates vs. foci representing stress-induced structures is not mutually exclusive. Statements to this effect should be removed or scaled down.

2) Reliance on only one ER/IF marker is insufficient for deriving firm conclusions regarding ALs. EM is essential here and would allow the authors to discern condensates from aggregates or ALs.

3) Figure 5: here a broader experimental basis is needed and the data should be recapitulated with additional depletions. Also, imaging should be performed with additional Nups independent of NUP358 as condensation of other NUPs might be completely missed. EM analysis and use of additional NUP markers (e.g. Mb414) is recommended. How would a channel NUP (e.g. NUP62) behave here as this NUP does not interact with NUP358? Lastly, would a NUP98 knockdown be lethal?

4) Related to the previous point, the rationale for cherry-picking specific NUPs for some but not all experiments (readouts+depletions) is unclear to this reviewer. While not all NUPs need to be compared throughout each and every experiment, It is recommended to use Nup98/62/214 throughout the ms both for depletions and as readouts to allow for more meaningful/direct comparisons.

5) In the context of depletions of enzymatic activities, the authors cannot exclude indirect effects, and no data are provided to monitor the phosphorylation or glycosylation state of any Nup. It is suggested to tone down conclusions and formally acknowledge the possibility of indirect effects or to remove this section altogether.

Minor points:

1) Fig 1C: can the authors comment of the extremely large extranuclear, red foci? Is this an artifact?

2) In Figure 2, why does G3BP not localize to stress granules at some basal level or upon heat stress?

3) In the abstract and introduction, the authors mention that FG-Nups are maintained by chaperones. The term chaperone might not be ideal as it implies that proteins of the protein quality control system are involved. However, only two NTRs are investigated here. Even though NTRs serve as "chaperones" for NPC components, it is suggested to rephrase to "chaperone activity of NTRs" or something similar.

4) Specific statements are made for the solubility limit of NUPs, but this is not directly shown anywhere. What is the solubility limit of these Nups, at which saturation point do they start to form foci, and are they really at their limit in these cells? Presumably this depends not only on absolute concentrations/state diagram considerations but also on chaperone capacity, availability of interactions partners anchoring certain NUPs to the NPC, etc. These considerations could be discussed more comprehensively e.g. in the discussion.

5) In aged cells the mislocalization of Nups could also have other causes. Perhaps in aged cells other (long-lived) NPC components get damaged or are not properly turned over, leading to a loss of FG-Nup incorporation.

6) Related to major point #5: Figure 7: what would be the effect of overexpression of Nup214 (as used in Fig. 4) on toxicity? Is toxicity a NUP98-specific property or do several NUPs exhibit toxicity in this context?

Referee #3:

Major findings
The manuscript 'Cytoplasmic nucleoporin foci are stress-sensitive, non-essential condensates in C. elegans' characterizes the composition and distribution of cytoplasmic Nup foci, and explores their regulation and role in nuclear pore assembly at the nuclear envelope. The main claims of the manuscript are that cytoplasmic Nup foci condense only in the cytoplasm of cells with high levels of Nups; a combination of phosphorylation, GlcNAcylation, and CRM-1 inhibit the formation of ectopic cytoplasmic Nup foci which are toxic in neurons, and the cytoplasmic foci do not act as intermediates to supply pre-made nuclear pores for use in embryogenesis, as has been proposed in Drosophila. The authors also validate and extend prior studies that show cytoplasmic Nup foci increase in oocytes during stress and extended meiotic arrest.

Overall impressions
This study builds significantly on prior foundational work examining cytoplasmic nucleoporin foci in the C. elegans germline. The experiments are well-designed with appropriate controls. A strength of the study is the systematic approach to cataloging 16 endogenous Nups which revealed a subset of Nups that localize to cytoplasmic foci. In addition, the paper is clearly written and has a logical, overall flow. Another strength of this manuscript is the inclusion of somatic tissues with the germline, in particular the novel finding that while cytoplasmic Nup foci do not have any deleterious roles in oocytes, ectopic Nup foci in neurons are toxic at a cellular and physiological level. This result is particularly intriguing alongside the finding of ectopic Nup foci in old-aged somatic cells. As the paper is currently framed, the emphasis is more on what cytoplasmic Nup foci are not doing, rather than a mechanism or function, and therefore it seems to be of moderately high significance.

Major concerns
1. To bolster the conclusion that cytoplasmic Nup foci are non-essential, additional experiments with arrested or heat-stressed oocytes would be very helpful to better understand how similar or different the Nup foci in those contexts are to those in growing oocytes. Because in arrested and heat-stressed oocytes, there are increased numbers of Nup foci, a few straightforward experiments would address if Nup foci are more generally non-essential, or if that is specific to growing oocytes: 1) Deplete cytoplasm-facing FG-Nups in arrested or heat-stressed oocytes; is this sufficient to abolish cytoplasmic Nup foci as it is growing oocytes? 2) Does hexanediol treatment dissolve the larger Nup foci in arrested or heat-stressed foci? 3) are there are any effects of the nup214 deletion strain on arrested oocytes, heat-stressed oocytes, or after fertilization of those oocytes/ during embryogenesis? Is it possible that cytoplasmic Nup foci have a role independent from nuclear pore assembly at the nuclear envelope? Given the current experiments, it seems more accurate to specifically state the Nup foci are non-essential in growing oocytes in regards to nuclear pore assembly, rather than the more general statement that Nup foci are non-essential structures.

2. In three sub-sections, relevant findings from the earlier literature are not included as part of the background /rationale for experiments. A small number of experiments here are validating prior work, and it seems important to include that context.
a. Fig. 1B. Nups were reported in cytoplasmic foci in C. elegans oocytes and P2 blastomeres of 4-cell embryos in 2000 (Pitt et al); therefore, Fig. 1B validates prior findings. It is worth noting a key advance of this study is the use of individually tagged Nups, in contrast to the sole use of the mAb414 antibody in prior studies. The thorough and careful quantitation of the distribution of Nups within oocytes is also a significant advance.
b. Fig. 2A. An increase in the number and size of cytoplasmic Nup foci were reported in the meiotically-arrested oocytes of C. elegans and three related nematodes in 2007, which seems to have been overlooked (Jud et al., 2007). A heat stress-induced increase in nuclear blebbing, resulting in more cytoplasmic Nup foci, was shown using mAb414 and TEM in Patterson et al., 2011 (as well as increases in nuclear blebbing in arrested oocytes). Therefore the Nup88 and Nup358 panels in Fig 2A validate prior findings with the mAb414 Ab.

c. Given the prior work that demonstrate Nup foci are stress-sensitive, I recommend modifying the title of the manuscript to instead emphasize the novel findings of this work, and replace that part of the title, perhaps with "germ cell-specific" or 'FG-specific Nups localize to cytoplasmic foci', or the novel finding that 'ectopic Nup foci are toxic in neurons'.
d. Fig 5B. Cytoplasmic Nup foci (stained by mAb414) were shown to disassemble in a cell ccyle dependent manner, in early embryos at mitosis in Pitt et al., 2000.

3. To assist readers, where you describe co-staining experiments for Figs S3A and D, I suggest including the information that mAb414 recognizes Nup 358, 214, 153, and 62. Or this information could be included in the description of Fig. 1.

4. Fig. 3. Annulate lamellae (AL) are unambiguously detected using TEM; however, due to the limitations of examining random thin sections, any determination of the % of oocytes containing AL using this approach will be an undercount. In prior analyses of random thin sections by Patterson et al, it is true that unambiguous AL were found in only ~10% of arrested oocytes in C. elegans, but they were also detected in 42% of arrested oocytes in the closely related nematode C. remanei. They were also detected in 20% of heat-stressed C. elegans oocytes. To be clear, I do not disagree with the authors' conclusion that the majority of Nup foci are very unlikely to be AL. Rather, I think it's important to clarify that AL have been detected in a fairly significant % of arrested/stressed oocytes (with the additional caveat that the sample sizes in TEM experiments the Patterson study was very low). Moreover, in the legend for Fig. S3, 42% of Nup foci overlap with ER/HDEL in arrested oocytes, which aligns quite well with the finding of 42% using TEM in C. remanei arrested oocytes and should be discussed. In the text, instead of stating the % of Nup foci that did not fully overlap with ER, it would seem more straightforward to state the % that do overlap. I am unsure what is meant by 'partial overlap' or how to interpret partial overlap. It seems the authors interpret it as inconsistent with AL, but I am not sure why? In the Discussion of these data, it would seem important to also interpret the 42% of Nup foci that do localize to ER membranes in arrested oocytes; do the authors consider this to be validation of AL in arrested oocytes? The focus on the majority of foci seems reasonable, but there appear to be two pools of cytoplasmic Nup foci, and possible differences in growing vs. arrested oocytes.

5. The data in Fig. 4 in support of the idea that cytoplasmic Nup foci form solely due to high concentration appear compelling at first glance. However, there is no discussion of the equally compelling data showing increased nuclear blebbing in arrested and heat-stressed oocytes (Patterson et al, 2011; Hetzer et al., 2005). Is it possible that in Day 1 adults with growing oocytes, Nup foci condense largely due to high concentrations (although there are low levels of blebbing in growing oocytes), and in arrested/heat-stressed oocytes a combination of: 1) Nup trafficking via increased nuclear blebbing, and 2) high concentrations promoting condensation, contribute to additional Nup foci in arrested/stressed oocytes?

6. I was surprised the current discussion currently lacks any mention of a robust connection between cytoplasmic Nup foci and RNP granules. In growing oocytes, cytoplasmic mAb414 foci are closely associated with PGL-1 germ granules (Pitt et al., 2000). In arrested oocytes, cytoplasmic mAb414 foci are adjacent to MEX-3 granules, and assembly of MEX-3 granules requires Nup 358 (Patterson et al., 2011). Given these reports of close protein associations in the cytoplasm, how do the authors reconcile the idea that cytoplasmic Nup foci form spontaneously, accidentally, and have no function? Because it is difficult/impossible to prove a lack of any function, it seems important to consider alternative models and soften some conclusions (including the title of Fig. 5), and distinguish between possible functions (or lack thereof) of cytoplasmic Nup foci in growing oocytes compared to arrested/stressed oocytes.

7. Fig 6. How many nuclear transport receptors were tested as regulators of cytoplasmic Nup foci? How many are there in C. elegans? If CRM1 and transportin were the only two tested, what was the rationale to select these two? In the Discussion of these data, it seems an overstatement to say Nup solubility depends primarily on CRM1 unless many other candidates were tested?

8. As mentioned above, in the first paragraph of the Discussion, and in the section titled "Must Nup foci are unlikely to serve an essential biological role and are potentially toxic" it would be helpful to clarify that you mean aging-induced Nup foci can be toxic in somatic or post-mitotic cells. This is eventually clarified where you state the deleterious effects are likely context dependent.

Minor concerns
1. I did not find any discussion as to why depletion of Nup 35, ndc-1, or gp210 might result in increased cytoplasmic Nup foci. This is an interesting finding, and some discussion seems warranted.
2. Could title of Fig. 7 include 'Ectopic Nup98 foci in neurons...' for clarity?
3. Videos S1 and S2 seem to show the same point, that Nup foci disassemble at mitosis; one could be omitted.
4. The rationale or logic for linking low abundance as evidence against the possibility of Nup foci as AL or pore precursors would be helpful. In the Discussion, contrasts are made between fly oogenesis and worm oogenesis; what is the abundance of Nup foci in fly oocytes? If it's higher than 3%, that would be helpful to include.
5. GlcNAcylation promotes SG and PB condensation which seems to argue against the idea that this modification plays a general solubilizing role for proteins (Ohn et al., 2008). Many post-translational modifications can either promote or inhibit condensation in a protein- and context-dependent manner, and GlcNAcylation appears to act similarly.

Referee #1:

In this paper, the authors observe and systematically study cytoplasmic nucleoporin (Nup) foci in C. elegans. In young C. elegans, cytoplasmic foci containing Nup358 and Nup88 are observed in gametes and early embryos, but not in somatic cells. Stress conditions such as oocyte arrest, heat stress, or aging also increased Nup foci assembly. Foci contain some types of Nups (ex, FG-Nups and cytoplasmic filament Nups) and their interactors (Y complex Nups, Nup88, RanGAP, NXF1) but not others (ex, transmembrane or nuclear Nups, Tpr...). The stoichiometry of the Nups in these foci was not fixed. The foci did not always associate with ER membranes. The authors concluded that the foci are unlikely to be annulate lamellae or nuclear pore complex assembly intermediates. Upon Nup214 depletion, foci number was greatly reduced, but the embryos were still viable and cargoes partitioned correctly, suggesting the formation of nuclear pore complexes. The authors conclude that since NPCs still form in the absence of foci, the foci do not contribute significantly to NPC assembly. When looking at the distributions of different Nups in the cell, the authors found that the majority show the following distribution: 30-40% nucleoplasm + NE, 60-70% cytoplasm, and around 3% in the foci. RNAi-mediated depletion of any foci-related Nups caused a general decrease in foci formation. On the other hand, depletion of non-foci Nups (such as transmembrane or nuclear) had no effect on the foci. Meanwhile, overexpression of Nup214 increased the amount of Nup358 in the foci. The authors conclude that Nup foci assembly depends on the cumulative effect of high concentrations of the cytoplasmic and inner channel FG-Nups in the cytoplasm, and the foci arise whenever the cytoplasmic concentration of FG-Nups exceeds their saturation concentration. Nup foci disassembled during mitosis, suggesting possible mechanisms to make Nups more soluble in the cytoplasm. The authors tested different proteins for their effect on reducing foci (thus increasing Nup solubility). Depletion of kinases PLK1 and CDK1, decrease in Nup O-GlcNAcylation, and depletion of transport receptor Crm1 all increased the proportion of Nups in foci (suggesting these increase Nup solubility in the cytoplasm) while depletion of PP2A decreased the proportion of Nups in the foci. The authors concluded that Nups can get solubilized by phosphorylation, O-GlcNAcylation, or transport receptors such as Crm1.
Finally, over-expression of Nup98 in neurons resulted in its accumulation in cytoplasmic foci, partial depletion of endogeneous Nup62 from the NPC, transport defects, and neuronal dysfunction. The authors concluded that Nup cytoplasmic foci can be toxic for the cells.
In conclusion, the authors show that these foci are formed in certain cell types and under stress conditions, that they are probably formed due to the propensity of Nups to form foci after exceeding the saturation concentration in the cytoplasm.

I think it is very important that this study raises awareness that cytoplasmic FG foci might not be of physiological relevance in all cases. However, I also do not feel that their negativism about other people's work is sufficiently justified. E.g., while I really like the data and how it is presented in this work, I also find the work from e.g. Hampoelz et al (which they seem to criticize directly and indirectly) which was obtained in different species and different experimental settings very convincing.
I think the authors put too much emphasis on the non-essentiality of the foci (it is also in the title). Their arguments for this are:

1. The Nup foci seem to form whenever the cumulative concentration of foci Nup in the cytoplasm increases beyond the saturation concentration
2. Multiple mechanisms in the cell serve to 'dissolve' these foci by 'solubilizing' Nups in the cytoplasm
3. The Nup foci are unlikely to function in NPC biogenesis or Annulate Lamellae
Conclusion: The Nup foci have no essential function.

In my opinion, the conclusion does not completely follow. The Nup foci may well have functions outside of what the authors have probed. For example, they could have beneficial functions under specific circumstances. It is always difficult to prove that something has no function whatsoever. I also think that such a conclusion does not necessarily add much to the paper. It is enough to state that the foci form from Nups exceeding a certain concentration collectively and that not taking this into account can lead to misinterpretation in some (BUT NOT ALL) cases.

We agree with the reviewer that we have not – and cannot – discount that Nup foci provide some benefit under conditions that we have not yet tested. Our data, however, do suggest that the foci do not play an essential role in nuclear pore assembly and function during wild-type development. To better reflect this nuance, we have:

1. Changed the title (and the text) to specify that the Nup foci are not essential for nuclear pore biogenesis or viability
2. Performed new experiments to test whether robust Nup foci become essential in arrested oocytes (they do not).
3. Modified our discussion of the Hampoelz *et al*, 2019 results to make it clear that we do not dispute the finding that, in *Drosophila*, the Nup foci are used to concentrate nucleoporins in the oocyte (via transport from nurse cells) leading to the formation of annulate lamellae in oocytes. We do not dispute that in *Drosophila* annulate lamellae may contribute to nuclear pore assembly in embryos as suggested by an earlier study from the same group (Hampoelz *et al*, 2016). However, this function does not appear conserved in *C. elegans* oocytes which dissolve the majority of Nup foci (condensates and annulate lamellae) during the oocyte-to-embryo transition.
4. Expanded the Discussion to include the possibility that the Nup foci could become essential under conditions not yet tested (see line 526).

After addressing this important point and the points below (most of them do not request new experiemnts), I feel the paper would be in good shape to publish.

Major comments

1. In Figure 7A, S8 etc, the authors use C-terminally tagged Nup98. At which position in Nup98 did the authors insert the fluorescent protein? Could they provide a reference to show its functionality, or data to support this? See for example PMC2150585, Nup98 is produced either

as a fusion with Nup96 or with a shorter C-terminal region which is proteolytically cleaved. This could be important for Nup98's localization to the NPC. In Figure 7A, the rab-3p::Nup98-mNeon does not seem to localize to the NE. Is it because of the image brightness settings, or did the authors not observe any localization to the NE at all? Ectopically expressed Nup98 should also localize to the NPC. Usually, N-terminally tagged Nup98 is preferred. Moreover, is the effect of depleting other Nups from the NE/NPC only for Nup98 or is it general? For example, did the authors also observe a partial depletion in Nup62 (or other Nups) from the NE following Nup214 overexpression (mentioned in Figure 4)?

The reviewer is correct that the mNeonGreen tag was inserted at the C-terminus of Nup98: position 919 in NPP-10 (the *C. elegans* Nup98 homolog), which is the predicted cleavage site for the Nup98/Nup96 fusion precursor (Voronina & Seydoux, 2010).

To address the reviewer's concern, we have repeated our experiments with a new N-terminally tagged Nup98 fusion and obtained the same results (see Appendix Figures S8E-G). Both the N-terminal and C-terminal Nup98 fusions localize to the nuclear envelope when overexpressed, although at reduced levels compared to endogenous Nup98 (see Appendix Figures S8B and E).

In addition, as the reviewer predicted, we did find that overexpression of Nup214 in oocytes also leads to endogenous Nup depletion from the nuclear envelope (in this case Nup358, Figure 3D).

2. I expand here on the topic on Nup foci in C. elegans vs a role in NPC biogenesis or annulate lamellae (AL). It would be good in terms of readability if the authors expand on this by giving specific examples from literature or more detailed reasoning on how AL change from their foci. For example, here are the premises from the Discussion, as to why their foci are probably not AL's:
a. "1) lack Nups essential for pore assembly including transmembrane Nups" - authors could shortly explain that AL in (conditions, organisms, systems, etc) contain also transmembrane Nups b. "2) account for less than 3% of total Nup molecules," - How does this premise make it unlikely that the foci are AL? How much Nup should the AL contain? c. "3) display heterogeneous Nup stoichiometry," - what kind of stoichiometry do AL's show? d. "4) do not colocalize with membranes" - again, some short explanation on findings regarding AL's could be good.

To address these points, we have reorganized the Results section and the Discussion to acknowledge the possibility that, as shown in *Drosophila*, some Nup foci may mature into annulate lamellae. In particular, see the Discussion starting at line 497.

3. On page 8: the authors write: ""In vitro, FG-Nups readily condense into hydrogels (Labokha et al, 2012) raising the possibility that cytoplasmic Nup foci might form by spontaneous condensation of FG-Nups in the saturated environment of the oocyte." I feel strongly that this gives the wrong impression. FG Nups where shown to phase separate after pH shift or from

lyophilized powder or by diluting from highly concnetraetd denaturant. None of this I feel fit the description of "readily condense".

We have removed "readily" from line 205, which now states: "*In vitro*, FG-Nups condense into hydrogels". We have also added additional references (Schmidt & Görlich, 2015; Frey *et al*, 2006) to support that FG-Nups form condensates *in vitro.*

4. On page 9: ""FG-Nup hydrogels assembled in vitro are readily dissolved by the aliphatic alcohol 1,6-hexanediol (Schmidt & Görlich, 2015), which disrupts hydrophobic interactions and has been reported to dissolve Nup foci in yeast, Drosophila, and HeLa cells (Hampoelz et al, 2019b; Patel et al, 2007; Agote-Aran et al, 2020)." This statement also gives a wrong impression. The cited paper (Schmidt & Görlich, 2015) studied many Nups, but in fact shows only hexandiol solubilization for two selected Nups from specific species (S. cerevisiae Nup116 and Tetrahymena Mac98A FG). For other hydrogels it is has not been experimentally reported, and in fact, many FG Nup hdyrogels are probably not hexanediol sensitive.

We have clarified this point in the text line 245, stating: "*In vitro,* Nup98 FG-domain hydrogels have been shown to be dissolved by the aliphatic alcohol 1,6-hexanediol (Schmidt & Görlich, 2015)".

Minor comments

1. Figure 2 - % Nup in foci, how is that calculated? I did not see this.

Thank you for pointing this out. We have rearranged the Results section so the original Figure 2 is now Figure 4. With this new organization, the first time we measure the percent of Nup in foci (Figure 3A), we refer the reader to a description of this calculation, writing in line 211: "we used Imaris software to quantify Nup fluorescence in nuclei, the cytoplasm, and cytoplasmic foci (Appendix Figure S3A and see materials and methods)."

2. Figure S4C - maybe lower the x-axis so that the data for Nup35 depletion is more visible (it looks like it is zero but not seen)

The reviewer is correct that the Nup35 depletion is very close to zero. We have adjusted the y-axis so that that data is more visible (note that this panel is now Appendix Figure S3C).

3. Figure 5A - Caption says "Nup foci accumulate progressively throughout oocyte growth and peak in the - 3 and -4 oocytes" but figure doesn't show that. No need to be in the figure caption

We have removed the sentence "Nup foci accumulate progressively throughout oocyte growth and peak in the -3 and -4 oocytes" from the legend (note that this panel is now Appendix Figure S5A).

4. Figure 5C has quantification, 5D, S5C and S5D could also benefit from quantification.

As requested, we have added quantification as follows:

1. Quantification of the distribution of mNeonGreen::Nup358 in embryos to Figure 5D.
2. Quantification of the distribution of RanGAP::wrmScarlet to Appendix Figure S5D (originally Figure S5C).
3. Quantification of the number of embryos with the representative phenotype out of the total number of embryos imaged to Appendix Figure S5E (originally Figure S5D).

5. Most figures which claim co-localization lack quantification. Sometimes it is obvious from the positioning of the puncta/foci, however an analysis could make it better.

The reviewer is correct that we only quantified colocalization in Figure 2C, where we wanted to measure the stoichiometry of individual Nups in foci versus the nuclear envelope.

Elsewhere, we did not quantify colocalization as in those experiments the purpose was simply to document presence in foci. For those experiments in the text and figure legends, we have replaced "colocalization" with "enrichment" in foci to denote the qualitative observation that those proteins are present at higher concentrations in foci relative to the cytoplasm.

6. Figure S7E, it would be good to show the nuclei with an outline, especially in the control image.

We have added outlines to nuclei in Appendix Figure S7E for clarity and the following sentence to the legend "Red dashed lines denote nuclei.".

7. Similarly in Figure S8A, it is unclear from the figure that those are cytoplasmic foci, the cell and nuclei outline in an inset would make it clearer.

We have included an inset of a single cell with the nucleus denoted by Hoechst staining in Figure 7A.

8. Some figures show the bar graph with cytoplasmic, NE/nucleoplasm and foci % of total Nup (ex, Figure 4C). Some figures show Fold change of Nup in foci onl;y (ex, Figure 6A). I assume also Figure 6 is calculated from similar data, i.e. the nuclear, cytoplasmic and foci Nup was calculated. In that case, also showing the cytoplasmic and NE/nucleoplasm Nup amounts for the other figures could be beneficial. Especially in the parts where the authors make claims about Nup solubility, it would be good to confirm that cytoplasmic Nup amounts have increased while foci Nup amounts decreased.

Thank you for this suggestion. We have updated the quantification for the following figures:

1. Appendix Figure S4A: We have added raw quantification of the distribution of Nup between foci, the NE/nucleoplasm, and the cytoplasm corresponding to the normalized quantification presented in Figure 4A.
2. Appendix Figure S6B: We have added raw quantification of the distribution of Nup between foci, the NE/nucleoplasm, and the cytoplasm corresponding to the normalized quantification presented in Figure 6A.
3. Appendix Figure S7D: We have added quantification of the distribution of Nup358 between foci, the NE/nucleoplasm, and the cytoplasm.

10. Rephrase "Nup foci in oocytes only contain FG-Nups and their direct binding partners". It reads as if no other protein might be present in the foci. "... contain (...)-Nups and their partners, but not (...)-Nups" could read more accurately

As suggested, we have amended the text in lines 155 and 156 to state "Nup foci in growing oocytes contain FG-Nups and their direct binding partners, but not transmembrane, inner ring complex, or nucleoplasmic Nups"

11. Rephrase "As expected, we found that hexanediol treatment dissolved Nup foci in C. elegans, although it had no effect on Nups at the nuclear envelope (Figure S4H)" - as "NE stain remained", because from the images it cannot be seen that there is "no effect"; no quantification is provided.

As suggested, we have removed the statement regarding Nup localization to the NE, and stated in line 248: "As expected, we found that hexanediol treatment reduced the intensity of Nup foci in embryos (Appendix Figure S3H)."

12. The authors could rewrite the abstract to put more focus on their findings and less on the interpretations. "Our results suggest that Nup foci are non-essential, "accidental", and potentially toxic condensates whose assembly is actively suppressed in healthy cells" - this sentence is highly speculative. Over-expressed Nup98::mNeon was toxic but its functionality in the cell is not clear, (see major comment 1). Over-expression is non-physiological anyway. "Non-essential" is too generalizing, (see major comment 2). Active suppression of foci in healthy cells has also not been shown. The authors show that there are mechanisms which reduce the foci formation, potentially by increasing their solubility in the cytoplasm (although this has been deduced due to the apparent decrease in foci). However, if the authors claim that this is an "active suppression" they should provide more evidence for this.

We have addressed these concerns with the following:

1. New overexpression experiments using an N-terminally tagged Nup98 which we know is functional when not overexpressed (Appendix Figures S8E-G).
2. We have reworded the title: "Nucleoporin foci are stress-sensitive condensates dispensable for *C. elegans* nuclear pore assembly"

3. We now include a new experiment showing that *ogt* mutants accumulate Nup foci in somatic cells at Day 4 of adulthood (Figure 6D). Wild-type animals accumulate significantly fewer foci, providing additional evidence that cells suppress condensation in an OGT-dependent manner.
4. We rephrased the last sentence of the abstract to make it clear that, although based on our findings, that statement remains speculative: “We speculate that Nup foci are non-essential and potentially toxic condensates whose assembly is actively suppressed in healthy cells.”

Referee #2:

This study by Thomas et al. provides a phenotypic description of Nucleoporin (Nup) foci in C. elegans cells. Combining genetic perturbations and light microcopy, the authors observe that Nups form condensates in tissues that express high levels of Nups, including oocytes and embryos. The study furthermore shows that Nup foci consist mainly of FG-Nups and that reduction of certain Nups leads to reduction in Nup foci. Moreover, reduction of Nup214 results in viable embryos, while overexpression of Nup98 results in neuronal toxicity. Overall, this is an interesting study in an organismal context that takes a closer look at NUP condensation in relation to various stress conditions. However, the impact of this study is presently somewhat limited as only a few and variable NUPs are scrutinized in some of the key experiments. Here, a broader experimental footing is necessary to support the author’s key conclusions. Specifically, the inclusion of a consistent set of Nups both as readouts for condensates and their more widespread depletion throughout various assays is recommended to allow meaningful comparisons. In addition, the reliance on fluorescence microscopy as singlular readout seems insufficient and additional techniques (especially EM which is the standard in the field) seem essential for clarifying the relationship (or the lack thereof) between the ER membranes, condensates, and NPCs/annulate lamellae (Als). Lastly, (too) firm conclusions are made connecting NUP solubility and PTMs, but without monitoring NUP modifications directly. Overall, a significant number of additional experiments are required to support the present conclusions and more moderate, less generalizing statements are asked for.

Major points:

1) The authors tend to overinterpret their conclusions on several occasions (on a side note, page/line numbers would have been helpful to point these out). For example, the authors cannot rule out that NUP foci are intermediates of assembly in other organisms or in a distinct physiological context in the same organism. This limitation should be specifically stated. Note also that Hampoelz et al. showed recently in Drosophila that certain NPC components incorporate only later when the condensates transition into full NPCs. Therefore, a more time-resolved analysis monitoring several representative NUPs would be useful. Lastly, NUP accumulation in foci as part of a normal physiological function/assembly intermediates vs. foci

representing stress-induced structures is not mutually exclusive. Statements to this effect should be removed or scaled down.

To address these comments:

1. We now include new figures (Appendix Figures S2C and S4B) where we examine the localization of an inner ring complex Nup and a nucleoplasmic Nup in growing oocytes and arrested oocytes. We find that these Nups incorporate in foci only in the arrested oocytes. These data are consistent with the observations in *Drosophila* oocytes which suggest that Nup foci can mature into annulate lamellae. We now acknowledge this possibility in the Discussion starting in line 497.
2. In new figures (Figure 5A and Appendix Figures S5L and M), we demonstrate that Nup foci assembled in arrested oocytes disassemble during the oocyte-to-embryo transition, and a mutant (*nup214Δ*) with significantly reduced foci still produces viable embryos from arrested oocytes. We conclude that, even though Nup foci may mature into annulate lamellae in the context of long-term oocyte arrest, these structures are not maintained during the oocyte-to-embryo transition and are not essential for embryogenesis at least under the conditions tested.
3. We have modified our discussion of the Hampoelz *et al*, 2019 results to make it clear that we do not dispute the finding that, in *Drosophila*, the Nup foci are used to concentrate nucleoporins in the oocyte (via transport from nurse cells). This leads to the formation of annulate lamellae in oocytes, which in that system may contribute to nuclear pore assembly in embryos, as suggested by an earlier study from the same group (Hampoelz *et al*, 2016). However, as described above, our data do not support an essential role for Nup foci and annulate lamellae in nuclear pore assembly in *C. elegans*, even in context of long-term arrested oocytes.
4. We have expanded the Discussion to include the possibility that the Nup foci could become essential under conditions not yet tested (see line 526).

2) Reliance on only one ER/IF marker is insufficient for deriving firm conclusions regarding ALs. EM is essential here and would allow the authors to discern condensates from aggregates or ALs.

EM analyses have already been performed by the Priess and Schisa labs who found that annulate lamellae are not present in wild-type growing oocytes or embryos and are observed in a minority of arrested oocytes (Langerak *et al*, 2019; Patterson *et al*, 2011; Pitt *et al*, 2000). Consistent with their findings, we find that only a minority of Nup foci colocalize with the ER using three independent ER markers: the KDEL reporter (Figure 2D and Appendix Figures S4C; Fan *et al*, 2020; Lee *et al*, 2016), the transmembrane Nup gp210 (Figure 2C; Galy *et al*, 2008), and the transmembrane Nup NDC1 (Appendix Figure S2A; Mauro *et al*, 2022).

3) Figure 5: here a broader experimental basis is needed and the data should be recapitulated with additional depletions. Also, imaging should be performed with additional Nups independent of NUP358 as condensation of other NUPs might be completely missed. EM

analysis and use of additional NUP markers (e.g. Mb414) is recommended. How would a channel NUP (e.g. NUP62) behave here as this NUP does not interact with NUP358? Lastly, would a NUP98 knockdown be lethal?

As suggested by the reviewer, we extended our analyses bringing the total number of Nups analyzed in the *nup214Δ* mutant to four:

1. Nup85 (new data in Appendix Figure S5C)
2. Nup358 (Figures 5C and D)
3. RanGAP (Appendix Figure S5D)
4. mAb414 (Appendix Figure S5E), as suggested by the reviewer

We considered extending our analyses to Nup62 as suggested by the reviewer but decided against it as Nup62 interacts with Nup214 (von Appen *et al*, 2015).

Unfortunately, we were not able to use knockdowns of Nup358, Nup98, or Nup62 as loss of each of these Nups causes high embryonic lethality (Galy *et al*, 2003). We were able, however, in new experiments to analyze *nup88Δ* mutants which have significantly reduced foci (Appendix Figures S5C and I), yet are viable Appendix (Figure S5J) and have normal partitioning of the IBB domain reporter (Appendix Figure S5K), confirming our findings with the *nup214Δ* mutant.

4) Related to the previous point, the rationale for cherry-picking specific NUPs for some but not all experiments (readouts+depletions) is unclear to this reviewer. While not all NUPs need to be compared throughout each and every experiment, It is recommended to use Nup98/62/214 throughout the ms both for depletions and as readouts to allow for more meaningful/direct comparisons.

We have performed all key experiments using two consistent Nups as standard reporters: Nup88 and Nup358 (see table below). These Nups were chosen as they were identified in our survey as having the highest degree of enrichment in cytoplasmic foci (see Figure 3A). Additionally, both Nup88 and Nup358 localize to the cytoplasmic face of nuclear pores, allowing us to use the surface tool in Imaris to accurately quantify the percent of Nup88 and Nup358 at the nuclear envelope. Other Nups, including Nup98 and Nup62, partially localize to the nucleoplasm, complicating measurement of the percent of Nup localized specifically to pores at the nuclear envelope. Finally, as noted in lines 131-135, both Nup88 and Nup358 have been shown to localize to cytoplasmic Nup foci in other organisms and cell types.

In several figures we were unable to use Nup88 or Nup358 as reporters, or felt that alternative Nups would serve as more suitable reporters. The rationale for choice of Nup reporters in all experiments is detailed in the following table:

Figure	Brief description of	Nup(s) used	Quantitative	Rationale for choice

	experiment	as reporters	vs. qualitative analysis	of Nup reporter(s)
1B, S1A	Survey of Nup foci across tissues	Nup358, Nup88	Qualitative	Standard reporter Nups
1C, S1C	Characterization of Nup foci in embryos	Nup358, Nup88	Qualitative	Standard reporter Nups
1D	Quantification of cytoplasmic Nup levels in different tissues	Nup358	Quantitative	Standard reporter Nup
S1B	Characterization of Nup foci in the male germline	Nup88	Qualitative	Standard reporter Nup
S1D	Characterization of Nup foci in Day 1 versus Day 2 adults	Nup358, Nup88, Nup98, Nup62	Quantitative	Standard reporter Nups, plus two additional FG-Nups
2B, S2B	Survey of Nups and Nup-binding proteins in growing oocytes	Nup358, Nup88, Nup214, Nup98, Nup62, Nup54, Nup96, Nup85, Nup107, Nup35, gp210, NDC1, Nup153, ELYS, TPR, Nup50, RanGAP, NXF1	Qualitative	These Nups are representative members of all NPC sub-complexes
S2A, S2E	Immunostaining to test whether Nup foci contain multiple Nups	Nup358, Nup88, Nup214, Nup96, NDC1, mAb414	Qualitative	Standard reporter Nups; mAb414 recognizes multiple Nups (and is thus a useful marker to test whether foci contain multiple Nups
S2C	Time-resolved characterization of Nups in growing oocytes	Nup88, TPR, Nup35	Qualitative	Standard reporter Nup with two representative Nups not present in foci

S2D	Survey of Nups in early embryos	Nup358, Nup88, Nup98, Nup85, Nup35, Nup62, TPR, ELYS, NDC1, gp210	Qualitative	These Nups represent all endogenous CRISPR-tagged Nups from the survey in Figure 2B
2C	Colocalization analysis of GFP-tagged Nups versus Nup62::wrmScarlet	Nup358, Nup88, Nup98, Nup85, gp210, ELYS	Quantitative	These are all endogenous CRISPR GFP-tagged Nups that localize to foci, with two representative Nups not present in foci
2D	Characterization of the overlap of Nup foci and membranes	Nup88	Quantitative	Standard reporter Nup
3A	Quantification of the distribution of Nups in growing oocytes	Nup358, Nup88, Nup98, Nup62, Nup85, Nup35, gp210, NDC1, ELYS, TPR	Quantitative	These Nups are all endogenous CRISPR-tagged Nups that are representative members of all NPC sub-complexes
3B	RNAi screen to test the role of individual Nups in foci formation	Nup85	Quantitative	We wanted to test the role of both Nup88 and Nup358 in foci formation, and thus could not use either standard reporter
3C	Characterization of Nup foci in the *gp210Δ* mutant	Nup358	Quantitative	Standard reporter Nup
S3E, S3F	Test of the role of Nup85, Nup35, and NDC1 in foci formation	Nup88	Quantitative	Standard reporter Nup
3D, S3G	Test of the effect of Nup214 overexpression on foci formation	Nup358	Quantitative	Standard reporter Nup
S3H	Test of the effect of 1,6-hexandiol on foci	Nup98	Qualitative	Our Nup98 strain had a PGL-

	formation			3::mCherry marker which was used as a positive control for hexanediol treatment
4A, S4B	Characterization of Nup foci in arrested oocytes	Nup88, ELYS, Nup35	Quantitative	Standard reporter Nup, plus two representative Nups that do not enrich in foci in growing oocytes
4A, 4B, S4D	Characterization of Nup foci following heat stress	Nup358, Nup88	Quantitative	Standard reporter Nups
4C, 4D	Characterization of somatic Nup foci in aged adults	Nup88	Quantitative	Standard reporter Nup
S4C	Characterization of the overlap of Nup foci and membranes in arrested oocytes	Nup88	Quantitative	Standard reporter Nup
5A, S5A, S5B	Characterization of Nup dynamics in maturing oocytes	Nup358, Nup88	Quantitative	Standard reporter Nups
5B, S5B	Characterization of Nup dynamics in early embryos	Nup88	Quantitative	Standard reporter Nup
5C, 5D, S5C, S5D, S5E, S5F	Characterization of Nup foci in the *nup214Δ* mutant	Nup358, Nup88, Nup85, RanGAP, mAb414	Quantitative	Standard reporter Nups, plus additional reporters as suggested by reviewer. This includes mAb414, which recognizes multiple Nups and is thus a useful marker to test depletion of foci
S5H	Quantification of Nup density at the nuclear envelope in the *nup214Δ* mutant	Nup35	Quantitative	Nup35 is a component of the inner ring complex that scaffolds nuclear pores, and thus an appropriate choice for measuring

				nuclear pore density
S5C and I	Characterization of Nup foci in the *nup88Δ* mutant	Nup85	Quantitative	We could not use the standard reporter Nup88 to test the *nup88Δ* mutant
S5L	Characterization of Nup foci in arrested oocytes of the *nup214Δ* mutant	Nup358	Quantitative	Standard reporter Nup
6A, 6B, S6B, S6C	Characterization of Nup foci following kinase and phosphatase depletion	Nup88	Quantitative	Standard reporter Nup
S6A	Localization of CDK1 versus Nup foci	Nup62	Qualitative	Nup62 is tagged with wrmScarlet and thus compatible with CDK1::GFP
6A, 6C, S6B, S6G	Characterization of Nup foci in *ogt* and *oga* mutants	Nup88	Quantitative	Standard reporter Nup
6D	Characterization of Nup foci in Day 4 adult *ogt* mutants	Nup88	Quantitative	Standard reporter Nup
S6D	Localization of OGT versus Nup foci	mAb414	Qualitative	The GFP secondary (rabbit) is compatible with the mAb414 secondary (mouse). mAb414 also recognizes multiple Nups, and is thus an appropriate marker for foci
S6E, S6F	GlcNAc staining in *ogt* and *oga* mutants	Nup358	Qualitative	Standard reporter Nup
6A, 6E, S6B, S7D	Characterization of Nup foci following CRM1 depletion and LMB treatment	Nup358, Nup85	Quantitative	Standard reporter Nup, plus one additional Nup reporter
S7B	Localization of NTRs versus Nup foci	Nup96	Qualitative	The mNeonGreen secondary (mouse) is compatible with the Nup96 secondary (rabbit).
S7F	Characterization of Nup	Nup358,	Qualitative	Standard reporter

	foci following transportin depletion	Nup85		Nup, plus one additional Nup reporter
7A, S8B	Localization of endogenous Nups with neuronal Nup98::mNeonGreen overexpression	Nup62	Quantitative	Nup62 is tagged with wrmScarlet and thus compatible with the Nup98::mNeonGreen transgene
7B, S8A	Localization of endogenous Nups in neurons	Nup62, Nup98	Qualitative	These panels are direct comparisons of endogenous Nup62 versus the Nup98::mNeonGreen transgene (Figure 7A)
S8F	Localization of endogenous Nups with neuronal mNeonGreen::Nup98 overexpression	Nup62	Qualitative	Nup62 is tagged with wrmScarlet and thus compatible with the mNeonGreen::Nup98 transgene

5) In the context of depletions of enzymatic activities, the authors cannot exclude indirect effects, and no data are provided to monitor the phosphorylation or glycosylation state of any Nup. It is suggested to tone down conclusions and formally acknowledge the possibility of indirect effects or to remove this section altogether.

We agree and we now acknowledge this point in the Discussion – see text starting at line 465.

Although we did not directly assay GlcNAcylation of specific Nups, we monitored the effect of the *ogt* mutation by immunofluorescence using the anti-GlcNAc RL2 antibody and observed, as expected, loss of GlcNAc signal at the nuclear envelope and Nup foci (Appendix Figures S6E and F).

Minor points:

1) Fig 1C: can the authors comment of the extremely large extranuclear, red foci? Is this an artifact?

These structures are polar bodies (meiotic products). We have clarified this by adding arrowheads and a note to the legend.

2) In Figure 2, why does G3BP not localize to stress granules at some basal level or upon heat stress?

G3BP condensation requires harsher stress conditions as reported by Abbatemarco *et al*, 2021. We have clarified this in the text starting in line 279.

3) In the abstract and introduction, the authors mention that FG-Nups are maintained by chaperones. The term chaperone might not be ideal as it implies that proteins of the protein quality control system are involved. However, only two NTRs are investigated here. Even though NTRs serve as "chaperones" for NPC components, it is suggested to rephrase to "chaperone activity of NTRs" or something similar.

We agree and have changed the text in the Abstract (line 40) and Introduction (line 114) as recommended. We also added quotations to “chaperones” in line 369.

4) Specific statements are made for the solubility limit of NUPs, but this is not directly shown anywhere. What is the solubility limit of these Nups, at which saturation point do they start to form foci, and are they really at their limit in these cells? Presumably this depends not only on absolute concentrations/state diagram considerations but also on chaperone capacity, availability of interactions partners anchoring certain NUPs to the NPC, etc. These considerations could be discussed more comprehensively e.g. in the discussion.

We agree that the FG-Nup solubility limit is a moving target dependent on several activities – we have indicated this in the Discussion starting in line 461.

5) In aged cells the mislocalization of Nups could also have other causes. Perhaps in aged cells other (long-lived) NPC components get damaged or are not properly turned over, leading to a loss of FG-Nup incorporation.

We have modified our discussion to avoid specifying any specific cause for Nup condensation in aged cells – see text in the Discussion starting in line 563.

6) Related to major point #5: Figure 7: what would be the effect of overexpression of Nup214 (as used in Fig. 4) on toxicity? Is toxicity a NUP98-specific property or do several NUPs exhibit toxicity in this context?

We have not tested whether other Nups overexpressed in neurons could also lead to toxicity.

Referee #3:

Major findings

The manuscript 'Cytoplasmic nucleoporin foci are stress-sensitive, non-essential condensates in C. elegans' characterizes the composition and distribution of cytoplasmic Nup foci, and explores their regulation and role in nuclear pore assembly at the nuclear envelope. The main claims of

the manuscript are that cytoplasmic Nup foci condense only in the cytoplasm of cells with high levels of Nups; a combination of phosphorylation, GlcNAcylation, and CRM-1 inhibit the formation of ectopic cytoplasmic Nup foci which are toxic in neurons, and the cytoplasmic foci do not act as intermediates to supply pre-made nuclear pores for use in embryogenesis, as has been proposed in Drosophila. The authors also validate and extend prior studies that show cytoplasmic Nup foci increase in oocytes during stress and extended meiotic arrest.

Overall impressions

This study builds significantly on prior foundational work examining cytoplasmic nucleoporin foci in the C. elegans germline. The experiments are well-designed with appropriate controls. A strength of the study is the systematic approach to cataloging 16 endogenous Nups which revealed a subset of Nups that localize to cytoplasmic foci. In addition, the paper is clearly written and has a logical, overall flow. Another strength of this manuscript is the inclusion of somatic tissues with the germline, in particular the novel finding that while cytoplasmic Nup foci do not have any deleterious roles in oocytes, ectopic Nup foci in neurons are toxic at a cellular and physiological level. This result is particularly intriguing alongside the finding of ectopic Nup foci in old-aged somatic cells. As the paper is currently framed, the emphasis is more on what cytoplasmic Nup foci are not doing, rather than a mechanism or function, and therefore it seems to be of moderately high significance.

Major concerns

1. To bolster the conclusion that cytoplasmic Nup foci are non-essential, additional experiments with arrested or heat-stressed oocytes would be very helpful to better understand how similar or different the Nup foci in those contexts are to those in growing oocytes. Because in arrested and heat-stressed oocytes, there are increased numbers of Nup foci, a few straightforward experiments would address if Nup foci are more generally non-essential, or if that is specific to growing oocytes: 1) Deplete cytoplasm-facing FG-Nups in arrested or heat-stressed oocytes; is this sufficient to abolish cytoplasmic Nup foci as it is growing oocytes? 2) Does hexanediol treatment dissolve the larger Nup foci in arrested or heat-stressed foci? 3) are there are any effects of the nup214 deletion strain on arrested oocytes, heat-stressed oocytes, or after fertilization of those oocytes/ during embryogenesis? Is it possible that cytoplasmic Nup foci have a role independent from nuclear pore assembly at the nuclear envelope? Given the current experiments, it seems more accurate to specifically state the Nup foci are non-essential in growing oocytes in regards to nuclear pore assembly, rather than the more general statement that Nup foci are non-essential structures.

We have modified the text as suggested and added new experiments to demonstrate that robust foci are also not required for viability in the context of arrested oocytes:

1. The percent of Nup in foci of arrested oocytes is decreased by ~40% in the *nup214Δ* mutant (Appendix Figure S5L). However, the embryonic viability of *nup214Δ* arrested oocytes following mating is the same as wild-type (Appendix Figure S5M).

2. The large foci assembled in arrested oocytes are fully disassembled in maturing oocytes following mating, and remain disassembled in newly fertilized zygotes (Figure 5A). This indicates that the large Nup foci in arrested oocytes are not maintained during the oocyte-to-embryo transition.
3. We agree with the reviewer that Nup foci may become essential under conditions not yet tested in this study, and have expanded this idea in the Discussion starting in line 526.

2. In three sub-sections, relevant findings from the earlier literature are not included as part of the background /rationale for experiments. A small number of experiments here are validating prior work, and it seems important to include that context.
a. Fig. 1B. Nups were reported in cytoplasmic foci in C. elegans oocytes and P2 blastomeres of 4-cell embryos in 2000 (Pitt et al); therefore, Fig. 1B validates prior findings. It is worth noting a key advance of this study is the use of individually tagged Nups, in contrast to the sole use of the mAb414 antibody in prior studies. The thorough and careful quantitation of the distribution of Nups within oocytes is also a significant advance.

We have referenced this study in line 129: "Cytoplasmic Nup foci have been observed in *C. elegans* oocytes and early embryos using the mAb414 antibody (Davis & Blobel, 1986; Pitt *et al*, 2000; Jud *et al*, 2007)."

We have also referenced these studies in line 107 of the introduction: "Cytoplasmic Nup foci were reported previously in *C. elegans* oocytes and embryos (Pitt *et al*, 2000; Sheth *et al*, 2010; Patterson *et al*, 2011; Jud *et al*, 2007)."

b. Fig. 2A. An increase in the number and size of cytoplasmic Nup foci were reported in the meiotically-arrested oocytes of C. elegans and three related nematodes in 2007, which seems to have been overlooked (Jud et al., 2007). A heat stress-induced increase in nuclear blebbing, resulting in more cytoplasmic Nup foci, was shown using mAb414 and TEM in Patterson et al., 2011 (as well as increases in nuclear blebbing in arrested oocytes). Therefore the Nup88 and Nup358 panels in Fig 2A validate prior findings with the mAb414 Ab.

We have now referenced these studies starting in line 270: "These findings are consistent with prior studies which found that the abundance of cytoplasmic Nup foci and nuclear blebs were significantly increased in arrested oocytes of *C. elegans* and related nematodes (Jud *et al*, 2007; Patterson *et al*, 2011).

c. Given the prior work that demonstrate Nup foci are stress-sensitive, I recommend modifying the title of the manuscript to instead emphasize the novel findings of this work, and replace that part of the title, perhaps with "germ cell-specific" or 'FG-specific Nups localize to cytoplasmic foci', or the novel finding that 'ectopic Nup foci are toxic in neurons'.

We have changed the title to: "Nucleoporin foci are stress-sensitive condensates dispensable for *C. elegans* nuclear pore assembly"

d. Fig 5B. Cytoplasmic Nup foci (stained by mAb414) were shown to disassemble in a cell ccyle dependent manner, in early embryos at mitosis in Pitt et al., 2000.

We have referred to this and an additional study starting in line 301: “These observations suggest that FG-Nup solubility oscillates with the cell cycle, peaking during M phase, consistent with prior studies (Pitt *et al*, 2000; Onischenko *et al*, 2005).”

3. To assist readers, where you describe co-staining experiments for Figs S3A and D, I suggest including the information that mAb414 recognizes Nup 358, 214, 153, and 62. Or this information could be included in the description of Fig. 1.

We have added this information in the description of Appendix Figures S2A and E (originally Figures S3A and D) starting in line 186: “Co-staining experiments using the mAb414 antibody, which in vertebrates recognizes Nup62, Nup153, Nup214, and Nup358, suggested that Nup foci contain multiple Nups (Appendix Figures S2A and E)”.

4. Fig. 3. Annulate lamellae (AL) are unambiguously detected using TEM; however, due to the limitations of examining random thin sections, any determination of the % of oocytes containing AL using this approach will be an undercount. In prior analyses of random thin sections by Patterson et al, it is true that unambiguous AL were found in only ~10% of arrested oocytes in C. elegans, but they were also detected in 42% of arrested oocytes in the closely related nematode C. remanei. They were also detected in 20% of heat-stressed C. elegans oocytes. To be clear, I do not disagree with the authors' conclusion that the majority of Nup foci are very unlikely to be AL. Rather, I think it's important to clarify that AL have been detected in a fairly significant % of arrested/stressed oocytes (with the additional caveat that the sample sizes in TEM experiments the Patterson study was very low). Moreover, in the legend for Fig. S3, 42% of Nup foci overlap with ER/HDEL in arrested oocytes, which aligns quite well with the finding of 42% using TEM in C. remanei arrested oocytes and should be discussed. In the text, instead of stating the % of Nup foci that did not fully overlap with ER, it would seem more straightforward to state the % that do overlap. I am unsure what is meant by 'partial overlap' or how to interpret partial overlap. It seems the authors interpret it as inconsistent with AL, but I am not sure why? In the Discussion of these data, it would seem important to also interpret the 42% of Nup foci that do localize to ER membranes in arrested oocytes; do the authors consider this to be validation of AL in arrested oocytes? The focus on the majority of foci seems reasonable, but there appear to be two pools of cytoplasmic Nup foci, and possible differences in growing vs. arrested oocytes.

We have changed the wording starting in line 195 to state the percent of Nup foci that do overlap with ER membranes: “We also found that only 20% of GFP::Nup88 foci in growing oocytes fully overlapped with a marker for endoplasmic reticulum membranes (Figure 2D).” We also changed the wording starting in line 266: “Furthermore, 42% of Nup foci in arrested oocytes overlapped with a marker for endoplasmic reticulum membranes (Appendix Figure

S4C), raising the possibility that a subset of Nup foci in arrested oocytes could correspond to annulate lamellae."

In the Discussion starting in line 497, we state the possibility that, in arrested oocytes, Nup condensates may have the potential to mature into annulate lamellae.

We have also described the overlap quantification in the Materials and methods starting in line 759: "To quantify the overlap of GFP::Nup88 with membranes (Figure 2D and Appendix Figure S4C), Z stacks of oocytes expressing GFP::Nup88 and the HaloTag::HDEL reporter were manually scored into 3 categories: 1. Complete overlap (the entire Nup88 focus overlapped with HaloTag::HDEL); 2. Partial overlap (the Nup88 focus partially overlapped or was directly adjacent to HaloTag::HDEL); 3. No overlap (the Nup88 focus did not directly contract membranes marked by HaloTag::HDEL)." We have added notes to the legends for Figure 2D and Appendix Figure S4C directing the reader to this section.

5. The data in Fig. 4 in support of the idea that cytoplasmic Nup foci form solely due to high concentration appear compelling at first glance. However, there is no discussion of the equally compelling data showing increased nuclear blebbing in arrested and heat-stressed oocytes (Patterson et al, 2011; Hetzer et al., 2005). Is it possible that in Day 1 adults with growing oocytes, Nup foci condense largely due to high concentrations (although there are low levels of blebbing in growing oocytes), and in arrested/ heat-stressed oocytes a combination of: 1) Nup trafficking via increased nuclear blebbing, and 2) high concentrations promoting condensation, contribute to additional Nup foci in arrested/stressed oocytes?

We point out that we do see enhanced nuclear blebbing in arrested oocytes as described by Patterson *et al*, starting in line 269. We do not know whether the nuclear blebbing could contribute to condensation and therefore prefer not to speculate.

6. I was surprised the current discussion currently lacks any mention of a robust connection between cytoplasmic Nup foci and RNP granules. In growing oocytes, cytoplasmic mAb414 foci are closely associated with PGL-1 germ granules (Pitt et al., 2000). In arrested oocytes, cytoplasmic mAb414 foci are adjacent to MEX-3 granules, and assembly of MEX-3 granules requires Nup 358 (Patterson et al., 2011). Given these reports of close protein associations in the cytoplasm, how do the authors reconcile the idea that cytoplasmic Nup foci form spontaneously, accidentally, and have no function? Because it is difficult/impossible to prove a lack of any function, it seems important to consider alternative models and soften some conclusions (including the title of Fig. 5), and distinguish between possible functions (or lack thereof) of cytoplasmic Nup foci in growing oocytes compared to arrested/stressed oocytes.

In the title and text we have now specified that our conclusions refer simply to the observation that the foci are not essential for nuclear pore assembly.

We have also changed the title of Figure 5 to "Nup foci are transient condensates that are not required for nuclear pore biogenesis".

Finally, in the Discussion (starting line 526), we bring up the possibility that these foci may have some function unrelated to pore biogenesis, including the possibility of functional connections between Nup foci and RNP granules.

7. Fig 6. How many nuclear transport receptors were tested as regulators of cytoplasmic Nup foci? How many are there in C. elegans? If CRM1 and transportin were the only two tested, what was the rationale to select these two? In the Discussion of these data, it seems an overstatement to say Nup solubility depends primarily on CRM1 unless many other candidates were tested?

We tested one import receptor (transportin) and one export receptor (CRM1) as we found both NTRs to enrich in cytoplasmic Nup foci (see Appendix Figure S7B). We also focused on CRM1 as it has been shown to interact with the condensate nucleators Nup214 and Nup358 with uniquely high affinity. Because we only tested two NTRs, we have toned down our statement that chaperoning depends uniquely on CRM1 (lines 381-382 and 492-494).

8. As mentioned above, in the first paragraph of the Discussion, and in the section titled "Must Nup foci are unlikely to serve an essential biological role and are potentially toxic" it would be helpful to clarify that you mean aging-induced Nup foci can be toxic in somatic or post-mitotic cells. This is eventually clarified where you state the deleterious effects are likely context dependent.

Agreed and we have amended the text as suggested to specify post-mitotic cells (lines 406, 421, 536, and 538).

Minor concerns

1. I did not find any discussion as to why depletion of Nup 35, ndc-1, or gp210 might result in increased cytoplasmic Nup foci. This is an interesting finding, and some discussion seems warranted.

We have clarified the text describing this result starting in line 231: "As expected for structural Nups (Mansfeld *et al*, 2006; Mauro *et al*, 2022; Ródenas *et al*, 2009; Stavru *et al*, 2006; Onischenko *et al*, 2009), loss of Nup35 or the transmembrane Nups NDC1 or gp210 decreased Nup levels at the nuclear envelope (Figures 3B and C and Appendix Figures S3C and F), and enhanced foci formation, presumably because impaired pore assembly liberates FG-Nups to the cytoplasm."

This result has also been observed in yeast and HeLa cells, as described in the Discission starting in line 450 "Consistent with this hypothesis, depletion of scaffold nucleoporins that liberate FG-Nups enhance foci formation in *C. elegans* oocytes (Figure 3 and Appendix Figure S3), yeast (Makio *et al*, 2009), and HeLa cells (Raghunayakula *et al*, 2015)."

2. Could title of Fig. 7 include 'Ectopic Nup98 foci in neurons...' for clarity?

We agree and have changed the title to "Ectopic Nup98 foci in neurons deplete an endogenous Nup from the nuclear envelope and cause paralysis."

3. Videos S1 and S2 seem to show the same point, that Nup foci disassemble at mitosis; one could be omitted.

We agree, but Reviewer 2 has asked for multiple examples of different Nups. Therefore, we have opted to include both videos.

4. The rationale or logic for linking low abundance as evidence against the possibility of Nup foci as AL or pore precursors would be helpful. In the Discussion, contrasts are made between fly oogenesis and worm oogenesis; what is the abundance of Nup foci in fly oocytes? If it's higher than 3%, that would be helpful to include.

Hampoelz *et al*, 2019 did not report the percent of FG-Nup in foci in oocytes, only the % volume occupied by Nup358 foci (<1% of total egg chamber volume). Onischenko *et al,* 2004 also reported that the majority Nups are soluble in *Drosophila* embryos.

We have completely reworked our discussion of the Hampoelz papers – see the Discussion starting in line 497. We do not dispute that Nup foci may mature into annulate lamellae in arrested oocytes, as proposed in *Drosophila*. The main difference is that 1) in the *C. elegans* system, we can directly visualize the oocyte-to-embryo transition and find that FG-Nup foci fully disassemble and 2) we were able to directly test a requirement for Nup foci using two independent mutants: *nup214Δ* and *nup88Δ*.

5. GlcNAcylation promotes SG and PB condensation which seems to argue against the idea that this modification plays a general solubilizing role for proteins (Ohn et al., 2008). Many post-translational modifications can either promote or inhibit condensation in a protein- and context-dependent manner, and GlcNAcylation appears to act similarly.

Thank you for this point – we have amended the text starting in line 478: "Numerous studies have reported a protective role for O-GlcNAcylation in neurodegenerative disease (reviewed in Lee *et al*, 2021), raising the possibility that this modification plays a key role in solubilizing certain aggregation-prone proteins. A separate study found that O-GlcNAcylation promotes condensation of stress granules and P-bodies (Ohn *et al*, 2008), indicating that the solubilizing effect of O-GlcNAcylation is likely protein- and context-dependent."

We have made the following changes to figures:

1. We have replaced the oocyte image in Figure 1B with a unique image.
2. We have replaced the images in Figure S3B with unique images.
3. We have replaced the Day 2 image in Figure S1D with a unique image.

1st Revision - Editorial Decision 20th Apr 2023

Dear Geraldine,

Thank you for submitting a revised version of your manuscript. Your study has now been seen all original referees, who find that most of their previous concerns have been addressed and now recommend publication of the manuscript after a minor final revision. There remain only a couple of editorial points that have to be addressed before I can extend formal acceptance of the manuscript:
1. Please address the final minor comments by the referees.
2. Our publisher has done their pre-publication check on your manuscript. I have attached the file here. Please take a look at the word file and the comments regarding the figure legends and respond to the issues. Please also use this version when you resubmit the revised version.
3. We are missing the ORCID iD for the corresponding author. In order to link the ORCID iD to the account in our manuscript tracking system, the author in question has to do the following:
- Click the 'Modify Profile' link at the bottom of your homepage in our system.
- On the next page you will see a box halfway down the page titled ORCID*. Below this box is red text reading 'To Register/Link to ORCID, click here'. Please follow that link: you will be taken to ORCID where you can log in to your account (or create an account if you don't have one)
- You will then be asked to authorise Wiley to access your ORCID information. Once you have approved the linking, you will be brought back to our manuscript system.
We regret that we cannot do this linking on your behalf for security reasons.
4. Please fill in the "Sample definition and in-laboratory replication" section in the Author checklist (rows 87-88).
5. In the Data Availability section, please add a resolvable link to the dataset. More information about the format of this section can be found here: https://www.embopress.org/page/journal/14602075/authorguide#dataavailability

Please let me know if you have any questions regarding any of these points. You can use the link below to upload the revised files.

Thank you again for giving us the chance to consider your manuscript for The EMBO Journal. I look forward to receiving the final version.

With best wishes,

Ieva

Ieva Gailite, PhD
Senior Scientific Editor
The EMBO Journal
Meyerhofstrasse 1
D-69117 Heidelberg
Tel: +4962218891309
i.gailite@embojournal.org

Further information is available in our Guide For Authors: https://www.embopress.org/page/journal/14602075/authorguide

We realize that it is difficult to revise to a specific deadline. In the interest of protecting the conceptual advance provided by the work, we recommend a revision within 3 months (19th Jul 2023). Please discuss the revision progress ahead of this time with the editor if you require more time to complete the revisions.

Referee #1:

The authors have largely addressed my criticism. I would recommend the following minor changes.

Line 74: "Cohesive interactions among FG-Nups are critical for the formation of the permeability barrier and FG-Nup hydrogels recapitulate nuclear pore selectivity in vitro (Frey & Görlich, 2007; Hülsmann et al, 2012; Ng et al, 2021; Schmidt & Görlich, 2015; Strawn et al, 2004). These findings have led to the "selective phase" model in which the permeability barrier is established

by interactions among FG-Nups that form a phase separated network (Schmidt & Görlich, 2016; Ribbeck & Görlich, 2001)."
The logic of the two sentences and the actual publication dates are inverted.

Line 245: "In vitro, Nup98 FG-domain hydrogels have been shown to be dissolved by the aliphatic alcohol 1,6-hexanediol (Schmidt & Görlich, 2015)"
Some, not all all Nup98 FG-domain hydrogels have been shown to be hexanediol sensitive

Line 2368: Recent studies have suggested that nuclear transport receptors (NTRs) function as "chaperones" to prevent aggregation of intrinsically disordered proteins, including Nup62 (Guo et al, 2018; Hutten et al, 2020; Hofweber et al, 2018; Khalil et al, 2022). We found that two endogenously tagged NTRs (CRM1 and transportin) are enriched in cytoplasmic Nup foci in C. elegans oocytes (Appendix Figures S7A and B).
That NTRs can prevent aggregation of Nups was first shown in "EMBO Rep. 2013 Feb;14(2):178-83." doi: 10.1038/embor.2012.204.

Line 413: " In this study, we report the first systematic examination of the incidence of Nup foci across all tissues in an intact animal."
Reconsider "First time statement".

Referee #2:

Overall, the authors addressed several of our concerns. While some the conclusions were overstated in the original version, these were appropriately scaled down in the revised manuscript and generalizations were removed. While not all of our suggestions were addressed experimentally (e.g. EM analysis), it seems unlikely that mechanistic advances could be derived from a detailed EM analysis or a broader testing of NUPs which we primarily suggested to advance the study mechanistically. As it is stands, the manuscript reports on cytosolic NUP condensation, and the data are of adequate quality for EMBO J. That said, the study is mostly descriptive and offers only limited mechanistic insight into the biology of NUP condensates. Regardless, the data may well be of interest to the field due to the physiological/organismal context in which the phenomenon is observed.

Referee #3:

This revised manuscript from the Seydoux lab includes significant changes to the manuscript that strengthen the original manuscript. The new experiments, reorganization of a few sections, and revised interpretations/modified discussion thoroughly address all of my concerns and suggestions, with one minor exception that can be easily remedied. Regarding Fig. 4A/line 270, in Patterson et al., 2011, the nuclear blebs induced by arrest and heat stress were defined as regions of multiple bilayer membranes, with a bubble-like appearance along, and near the nuclear envelope. The mAb414 staining detected blebs that appear as ring-like structures (see their Figs. 3,4). I don't see any evidence of similar blebs with any of the Nup markers in Figure 4, or in any other images of arrested or stressed oocytes. However, analyses of single confocal slices, instead of projections, can reveal the blebs as I can easily see them in several single slices of both arrested and stressed oocytes in your source files. Images of single slices could either be added to Figure 4 or in the supplement.

This intriguing and well-designed study will be an important contribution to the nucleoporin and condensate fields.

1. Please address the final minor comments by the referees.

We have addressed the final comments from the referees (see below).

2. Our publisher has done their pre-publication check on your manuscript. I have attached the file here. Please take a look at the word file and the comments regarding the figure legends and respond to the issues. Please also use this version when you resubmit the revised version.

We have responded to the comments regarding the figure legends.

3. We are missing the ORCID iD for the corresponding author. In order to link the ORCID iD to the account in our manuscript tracking system, the author in question has to do the following:
- Click the 'Modify Profile' link at the bottom of your homepage in our system.
- On the next page you will see a box halfway down the page titled ORCID*. Below this box is red text reading 'To Register/Link to ORCID, click here'. Please follow that link: you will be taken to ORCID where you can log in to your account (or create an account if you don't have one)
- You will then be asked to authorise Wiley to access your ORCID information. Once you have approved the linking, you will be brought back to our manuscript system.
We regret that we cannot do this linking on your behalf for security reasons.

We have followed the above instructions and received the message "Your ORCID was successfully linked to your account". Please let us know if we need to do anything else.

4. Please fill in the "Sample definition and in-laboratory replication" section in the Author checklist (rows 87-88).

We have responded to this section in the Author checklist.

5. In the Data Availability section, please add a resolvable link to the dataset. More information about the format of this section can be found here: https://www.embopress.org/page/journal/14602075/authorguide#dataavailability

We have added a link to the imaging data in the Data Availability section.

Referee #1:

The authors have largely addressed my criticism. I would recommend the following

minor changes.

Line 74: "Cohesive interactions among FG-Nups are critical for the formation of the permeability barrier and FG-Nup hydrogels recapitulate nuclear pore selectivity in vitro (Frey & Görlich, 2007; Hülsmann et al, 2012; Ng et al, 2021; Schmidt & Görlich, 2015; Strawn et al, 2004). These findings have led to the "selective phase" model in which the permeability barrier is established by interactions among FG-Nups that form a phase separated network (Schmidt & Görlich, 2016; Ribbeck & Görlich, 2001)."
The logic of the two sentences and the actual publication dates are inverted.

We have inverted these sentences and the publication dates as follows: "In the "selective phase" model of transport selectivity, the permeability barrier is established by cohesive interactions among FG-Nups that form a phase separated network (Ribbeck & Görlich, 2001; Schmidt & Görlich, 2016). In support of this model, interactions among FG-Nups are critical for the formation of the permeability barrier and FG-Nup hydrogels recapitulate nuclear pore selectivity *in vitro* (Frey & Görlich, 2007; Hülsmann *et al*, 2012; Ng *et al*, 2021; Schmidt & Görlich, 2015; Strawn *et al*, 2004)."

Line 245: "In vitro, Nup98 FG-domain hydrogels have been shown to be dissolved by the aliphatic alcohol 1,6-hexanediol (Schmidt & Görlich, 2015)"
Some, not all all Nup98 FG-domain hydrogels have been shown to be hexanediol sensitive

We have clarified this point by writing: "*In vitro*, some Nup98 FG-domain hydrogels have been shown to be dissolved by the aliphatic alcohol 1,6-hexanediol (Schmidt & Görlich, 2015)"

Line 2368: Recent studies have suggested that nuclear transport receptors (NTRs) function as "chaperones" to prevent aggregation of intrinsically disordered proteins, including Nup62 (Guo et al, 2018; Hutten et al, 2020; Hofweber et al, 2018; Khalil et al, 2022). We found that two endogenously tagged NTRs (CRM1 and transportin) are enriched in cytoplasmic Nup foci in C. elegans oocytes (Appendix Figures S7A and B). That NTRs can prevent aggregation of Nups was first shown in "EMBO Rep. 2013 Feb;14(2):178-83." doi: 10.1038/embor.2012.204.

Thank you for pointing this out, we have added the reference.

Line 413: " In this study, we report the first systematic examination of the incidence of Nup foci across all tissues in an intact animal."

Reconsider "First time statement".

We have re-written this sentence as: “In this study, we report the systematic examination of the incidence of Nup foci across all tissues in an intact animal.”

Referee #2:

Overall, the authors addressed several of our concerns. While some the conclusions were overstated in the original version, these were appropriately scaled down in the revised manuscript and generalizations were removed. While not all of our suggestions were addressed experimentally (e.g. EM analysis), it seems unlikely that mechanistic advances could be derived from a detailed EM analysis or a broader testing of NUPs which we primarily suggested to advance the study mechanistically.
As it is stands, the manuscript reports on cytosolic NUP condensation, and the data are of adequate quality for EMBO J. That said, the study is mostly descriptive and offers only limited mechanistic insight into the biology of NUP condensates. Regardless, the data may well be of interest to the field due to the physiological/organismal context in which the phenomenon is observed.

Referee #3:

This revised manuscript from the Seydoux lab includes significant changes to the manuscript that strengthen the original manuscript. The new experiments, reorganization of a few sections, and revised interpretations/modified discussion thoroughly address all of my concerns and suggestions, with one minor exception that can be easily remedied. Regarding Fig. 4A/line 270, in Patterson et al., 2011, the nuclear blebs induced by arrest and heat stress were defined as regions of multiple bilayer membranes, with a bubble-like appearance along, and near the nuclear envelope. The mAb414 staining detected blebs that appear as ring-like structures (see their Figs. 3,4). I don't see any evidence of similar blebs with any of the Nup markers in Figure 4, or in any other images of arrested or stressed oocytes. However, analyses of single confocal slices, instead of projections, can reveal the blebs as I can easily see them in several single slices of both arrested and stressed oocytes in your source files. Images of single slices could either be added to Figure 4 or in the supplement.

As suggested we have added representative single confocal slices of GFP::Nup88 in growing versus arrested oocytes to Figure S4A.

This intriguing and well-designed study will be an important contribution to the nucleoporin and condensate fields.

2nd Revision - Editorial Decision **10th May 2023**

Dear Geraldine,

Thank you for addressing the final editorial issues. I am now pleased to inform you that your manuscript has been accepted for publication.

Before we forward your manuscript to our publishers, I would like to propose a couple of minor changes in the article synopsis and abstract. I have also written a short blurb that will accompany the title of your manuscript in our online table of contents. Please take a look at the text below and in the attached manuscript text file and let me know if any corrections are necessary.

Blurb:
FG repeat-containing nucleoporins form non-essential, age- and stress-enhanced cytoplasmic condensates in germ cells and developing embryos.

Synopsis:
Highly cohesive, phenylalanine/glycine repeat-containing nucleoporins (FG-Nups) form the central channel of nuclear pores and also concentrate in cytoplasmic foci proposed to function as pore pre-assembly intermediates. This study in the C. elegans model shows that nucleoporin (Nup) foci are transient condensates that are not essential for pore assembly.

• Cellular Nup foci arise when FG-Nups accumulate at high levels in the cytoplasm exceeding their solubility limit, such as in oocytes and developing embryos.
• FG-Nup solubility is enhanced by posttranslational modifications, including GlcNAcylation and phosphorylation, as well as by chaperone activity.
• FG-Nup foci are transient structures that dissolve during M phase, when FG-Nup solubility increases.

Please note that it is EMBO Journal policy for the transcript of the editorial process (containing referee reports and your response letter) to be published as an online supplement to each paper. If you do NOT want this, you will need to inform the Editorial Office via email immediately. More information is available here:
https://www.embopress.org/page/journal/14602075/authorguide#transparentprocess

Your manuscript will be processed for publication in the journal by EMBO Press. Manuscripts in the PDF and electronic editions of The EMBO Journal will be copy edited, and you will be provided with page proofs prior to publication. Please note that supplementary information is not included in the proofs.

You will be contacted by Wiley Author Services to complete licensing and payment information. The required 'Page Charges Authorization Form' is available here: https://www.embopress.org/pb-assets/embo-site/tej_apc.pdf - please download and complete the form and return to embopressproduction@wiley.com

EMBO Press participates in many Publish and Read agreements that allow authors to publish Open Access with reduced/no publication charges. Check your eligibility: https://authorservices.wiley.com/author-resources/Journal-Authors/open-access/affiliation-policies-payments/index.html

Should you be planning a Press Release on your article, please get in contact with embojournal@wiley.com as early as possible, in order to coordinate publication and release dates.

If you have any questions, please do not hesitate to call or email the Editorial Office. Thank you for this contribution to The EMBO Journal and congratulations on a successful publication!

Best regards,

Ieva

Ieva Gailite, PhD
Senior Scientific Editor
The EMBO Journal
Meyerhofstrasse 1
D-69117 Heidelberg
Tel: +4962218891309
i.gailite@embojournal.org

EMBO Press Author Checklist

Corresponding Author Name: Geraldine Seydoux	
Journal Submitted to: The EMBO Journal	
Manuscript Number: EMBOJ-2022-112987R	

USEFUL LINKS FOR COMPLETING THIS FORM
The EMBO Journal - Author Guidelines
EMBO Reports - Author Guidelines
Molecular Systems Biology - Author Guidelines
EMBO Molecular Medicine - Author Guidelines

Reporting Checklist for Life Science Articles (updated January
This checklist is adapted from Materials Design Analysis Reporting (MDAR) Checklist for Authors. MDAR establishes a minimum set of requirements in transparent reporting in the life sciences (see Statement of Task: 10.31222/osf.io/9sm4x). Please follow the journal's guidelines in preparing your
Please note that a copy of this checklist will be published alongside your article.

Abridged guidelines for figures
1. Data
The data shown in figures should satisfy the following conditions:
- the data were obtained and processed according to the field's best practice and are presented to reflect the results of the experiments in an accurate and unbiased manner.
- ideally, figure panels should include only measurements that are directly comparable to each other and obtained with the same assay.
- plots include clearly labeled error bars for independent experiments and sample sizes. Unless justified, error bars should not be shown for technical
- if n<5, the individual data points from each experiment should be plotted. Any statistical test employed should be justified.
- Source Data should be included to report the data underlying figures according to the guidelines set out in the authorship guidelines on Data

2. Captions
Each figure caption should contain the following information, for each panel where they are relevant:
- a specification of the experimental system investigated (eg cell line, species name).
- the assay(s) and method(s) used to carry out the reported observations and measurements.
- an explicit mention of the biological and chemical entity(ies) that are being measured.
- an explicit mention of the biological and chemical entity(ies) that are altered/varied/perturbed in a controlled manner.
- the exact sample size (n) for each experimental group/condition, given as a number, not a range;
- a description of the sample collection allowing the reader to understand whether the samples represent technical or biological replicates (including how many animals, litters, cultures, etc.).
- a statement of how many times the experiment shown was independently replicated in the laboratory.
- definitions of statistical methods and measures:
  - common tests, such as t-test (please specify whether paired vs. unpaired), simple χ2 tests, Wilcoxon and Mann-Whitney tests, can be unambiguously identified by name only, but more complex techniques should be described in the methods section;
  - are tests one-sided or two-sided?
  - are there adjustments for multiple comparisons?
  - exact statistical test results, e.g., P values = x but not P values < x;
  - definition of 'center values' as median or average;
  - definition of error bars as s.d. or s.e.m.

Please complete ALL of the questions below.
Select "Not Applicable" only when the requested information is not relevant for your study.

Materials

Newly Created Materials	Information included in the manuscript?	In which section is the information available? (Reagents and Tools Table, Materials and Methods, Figures, Data Availability Section)
New materials and reagents need to be available; do any restrictions apply?	Yes	Materials and methods, Appendix Table S1

Antibodies	Information included in the manuscript?	In which section is the information available? (Reagents and Tools Table, Materials and Methods, Figures, Data Availability Section)
For **antibodies** provide the following information:<br>- Commercial antibodies: RRID (if possible) or supplier name, catalogue number and or/clone number<br>- Non-commercial: RRID or citation	Yes	Materials and methods

DNA and RNA sequences	Information included in the manuscript?	In which section is the information available? (Reagents and Tools Table, Materials and Methods, Figures, Data Availability Section)
**Short novel DNA or RNA including primers, probes**: provide the sequences.	Not Applicable	

Cell materials	Information included in the manuscript?	In which section is the information available? (Reagents and Tools Table, Materials and Methods, Figures, Data Availability Section)
**Cell lines:** Provide species information, strain. Provide accession number in repository **OR** supplier name, catalog number, clone number, and/**OR** RRID.	Not Applicable	
**Primary cultures:** Provide species, strain, sex of origin, genetic modification status.	Not Applicable	
Report if the cell lines were recently **authenticated** (e.g., by STR profiling) and tested for mycoplasma contamination.	Not Applicable	

Experimental animals	Information included in the manuscript?	In which section is the information available? (Reagents and Tools Table, Materials and Methods, Figures, Data Availability Section)
**Laboratory animals or Model organisms:** Provide species, strain, sex, age, genetic modification status. Provide accession number in repository **OR** supplier name, catalog number, clone number, **OR** RRID.	Yes	Materials and methods, Appendix Table S1
**Animal observed in or captured from the field:** Provide species, sex, and age where possible.	Not Applicable	
Please detail **housing and husbandry conditions**.	Yes	Materials and methods

Plants and microbes	Information included in the manuscript?	In which section is the information available? (Reagents and Tools Table, Materials and Methods, Figures, Data Availability Section)
**Plants:** provide species and strain, ecotype and cultivar where relevant, unique accession number if available, and source (including location for collected wild specimens).	Not Applicable	
**Microbes:** provide species and strain, unique accession number if available, and source.	Not Applicable	

Human research participants	Information included in the manuscript?	In which section is the information available? (Reagents and Tools Table, Materials and Methods, Figures, Data Availability Section)
If collected and within the bounds of privacy constraints report on age, sex and gender or ethnicity for all study participants.	Not Applicable	

Core facilities	Information included in the manuscript?	In which section is the information available? (Reagents and Tools Table, Materials and Methods, Figures, Data Availability Section)
If your work benefited from core facilities, was their service mentioned in the acknowledgments section?	Not Applicable	

Design

Study protocol	Information included in the manuscript?	In which section is the information available? (Reagents and Tools Table, Materials and Methods, Figures, Data Availability Section)

If study protocol has been **pre-registered, provide DOI in the manuscript**. For clinical trials, provide the trial registration number **OR** cite DOI.	Not Applicable	
Report the **clinical trial registration number** (at ClinicalTrials.gov or equivalent), where applicable.	Not Applicable	

Laboratory protocol	Information included in the manuscript?	In which section is the information available? (Reagents and Tools Table, Materials and Methods, Figures, Data Availability Section)
Provide DOI OR other citation details if **external detailed step-by-step protocols** are available.	Yes	Materials and methods

Experimental study design and statistics	Information included in the manuscript?	In which section is the information available? (Reagents and Tools Table, Materials and Methods, Figures, Data Availability Section)
Include a statement about **sample size** estimate even if no statistical methods were used.	Yes	Legends, materials and methods
Were any steps taken to minimize the effects of subjective bias when allocating animals/samples to treatment (e.g. **randomization procedure**)? If yes, have they been described?	Not Applicable	
Include a statement about **blinding** even if no blinding was done.	Not Applicable	
Describe **inclusion/exclusion criteria** if samples or animals were excluded from the analysis. Were the criteria pre-established?<br><br>If sample or data points were omitted from analysis, report if this was due to attrition or intentional exclusion and provide justification.	Yes	Materials and methods
For every figure, are **statistical tests** justified as appropriate? Do the data meet the assumptions of the tests (e.g., normal distribution)? Describe any methods used to assess it. Is there an estimate of variation within each group of data? Is the variance similar between the groups that are being statistically compared?	Yes	Legends, materials and methods

Sample definition and in-laboratory replication	Information included in the manuscript?	In which section is the information available? (Reagents and Tools Table, Materials and Methods, Figures, Data Availability Section)
In the figure legends: state number of times the experiment was **replicated** in laboratory.	Yes	Figure legends
In the figure legends: define whether data describe **technical or biological replicates**.	Yes	Figure legends

Ethics

Ethics	Information included in the manuscript?	In which section is the information available? (Reagents and Tools Table, Materials and Methods, Figures, Data Availability Section)
Studies involving **human participants**: State details of **authority granting ethics approval** (IRB or equivalent committee(s), provide reference number for approval.	Not Applicable	
Studies involving **human participants**: Include a statement confirming that **informed consent** was obtained from all subjects and that the experiments conformed to the principles set out in the WMA Declaration of Helsinki and the Department of Health and Human Services Belmont Report.	Not Applicable	
Studies involving **human participants:** For publication of **patient photos**, include a statement confirming that consent to publish was obtained.	Not Applicable	
Studies involving experimental **animals**: State details of **authority granting ethics approval** (IRB or equivalent committee(s), provide reference number for approval. Include a statement of compliance with ethical regulations.	Not Applicable	
Studies involving **specimen and field samples:** State if relevant **permits** obtained, provide details of authority approving study; if none were required, explain why.	Not Applicable	

Dual Use Research of Concern (DURC)	Information included in the manuscript?	In which section is the information available? (Reagents and Tools Table, Materials and Methods, Figures, Data Availability Section)
Could your study fall under dual use research restrictions? Please check biosecurity documents and list of **select agents and toxins** (CDC): https://www.selectagents.gov/sat/list.htm	Not Applicable	
If you used a select agent, is the security level of the lab appropriate and reported in the manuscript?	Not Applicable	
If a study is subject to dual use research of concern regulations, is the name of the **authority granting approval and reference number** for the regulatory approval provided in the manuscript?	Not Applicable	

Reporting

The MDAR framework recommends adoption of discipline-specific guidelines, established and endorsed through community initiatives. Journals have their own policy about requiring specific guidelines and recommendations to complement MDAR.

Adherence to community standards	Information included in the manuscript?	In which section is the information available? (Reagents and Tools Table, Materials and Methods, Figures, Data Availability Section)
State if relevant guidelines or checklists (e.g., **ICMJE, MIBBI, ARRIVE, PRISMA**) have been followed or provided.	Not Applicable	
For **tumor marker prognostic studies,** we recommend that you follow the **REMARK** reporting guidelines (see link list at top right). See author guidelines, under 'Reporting Guidelines'. Please confirm you have followed these guidelines.	Not Applicable	
For **phase II and III randomized controlled trials**, please refer to the **CONSORT** flow diagram (see link list at top right) and submit the CONSORT checklist (see link list at top right) with your submission. See author guidelines, under 'Reporting Guidelines'. Please confirm you have submitted this list.	Not Applicable	

Data Availability

Data availability	Information included in the manuscript?	In which section is the information available? (Reagents and Tools Table, Materials and Methods, Figures, Data Availability Section)
Have **primary datasets** been deposited according to the journal's guidelines (see 'Data Deposition' section) and the respective accession numbers provided in the Data Availability Section?	Yes	Data availability
Were **human clinical and genomic datasets** deposited in a public access-controlled repository in accordance to ethical obligations to the patients and to the applicable consent agreement?	Not Applicable	
Are **computational models** that are central and integral to a study available without restrictions in a machine-readable form? Were the relevant accession numbers or links provided?	Not Applicable	
If publicly available data were reused, provide the respective **data citations in the reference list**.	Not Applicable	
